# Integrated application of transcriptomics and metabolomics provides insights into acute hepatopancreatic necrosis disease resistance of Pacific white shrimp *Litopenaeus vannamei*

**DOI:** 10.1128/msystems.00067-23

**Published:** 2023-06-26

**Authors:** Mingzhe Sun, Yang Yu, Shihao Li, Yuan Liu, Xiaojun Zhang, Fuhua Li

**Affiliations:** 1 Chinese Academy of Sciences (CAS) and Shandong Province Key Laboratory of Experimental Marine Biology, Institute of Oceanology, Chinese Academy of Sciences, Qingdao, China; 2 Center for Ocean Mega-Science, Chinese Academy of Sciences, Qingdao, China; 3 Laboratory for Marine Biology and Biotechnology, Qingdao National Laboratory for Marine Science and Technology, Qingdao, China; 4 The Innovation of Seed Design, Chinese Academy of Sciences, Wuhan, China; The University of Maine, Orono, Maine, USA

**Keywords:** *Litopenaeus vannamei*, acute hepatopancreatic necrosis disease, disease resistance, transcriptome, metabolome

## Abstract

Acute hepatopancreatic necrosis disease (AHPND) has caused a huge economic loss to shrimp aquaculture. *Vibrio parahaemolyticus* (Vp_AHPND_) is regarded as a major causative agent of AHPND in the Pacific white shrimp *Litopenaeus vannamei*. However, knowledge about how shrimp resist to AHPND is very limited. In order to learn the molecular mechanisms underlying AHPND resistance of shrimp, comparison between disease-resistant family and susceptible family of *L. vannamei* were performed at transcriptional and metabolic levels. Integrated analysis of transcriptomics and metabolomics on hepatopancreas of shrimp, the target tissue of Vp_AHPND_, showed that significant differences existed between resistant family and susceptible family of shrimp. The susceptible family showed higher level of glycolysis, serine-glycine metabolism, purine and pyrimidine metabolism, but lower level of betaine-homocysteine metabolism in the hepatopancreas in comparison with the resistant family without Vp_AHPND_ infection. Curiously, Vp_AHPND_ infection induced up-regulation of glycolysis, serine-glycine metabolism, purine metabolism, pyrimidine metabolism, and pentose phosphate pathway, and down-regulation of betaine-homocysteine metabolism in resistant family. In addition, arachidonic acid metabolism and some immune pathways, like NF-κB and cAMP pathways, were up-regulated in the resistant family after Vp_AHPND_ infection. In contrast, amino acid catabolism boosted via PEPCK-mediated TCA cycle flux was activated in the susceptible family after Vp_AHPND_ infection. These differences in transcriptome and metabolome between resistant family and susceptible family might contribute to the resistance of shrimp to bacteria.

**IMPORTANCE:**

*Vibrio parahaemolyticus* (Vp_AHPND_) is a major aquatic pathogen causing acute hepatopancreatic necrosis disease (AHPND) and leads to a huge economic loss to shrimp aquaculture. Despite the recent development of controlling culture environment, disease resistant broodstock breeding is still a sustainable approach for aquatic disease control. Metabolic changes occurred during Vp_AHPND_ infection, but knowledge about the metabolism in resistance to AHPND is very limited. Integrated analysis of transcriptome and metabolome revealed the basal metabolic differences exhibited between disease-resistant and susceptible shrimp. Amino acid catabolism might contribute to the pathogenesis of Vp_AHPND_ and arachidonic acid metabolism might be responsible for the resistance phenotype. This study will help to enlighten the metabolic and molecular mechanisms underlying shrimp resistance to AHPND. Also, the key genes and metabolites of amino acid and arachidonic acid pathway identified in this study will be applied for disease resistance improvement in the shrimp culture industry.

## INTRODUCTION

Shrimp products provide an excellent source of aquatic animal protein and Pacific white shrimp *Litopenaeus vannamei* accounts for 51.7% of the global crustaceans’ production in 2020 (FAO, 2022) (1, 2). Acute hepatopancreatic necrosis disease (AHPND) has led to a huge economic loss in shrimp aquaculture since its outbreak in 2010s (3 - 6). The main causative agent of AHPND is *Vibrio parahaemolyticus* (Vp_AHPND_) carrying binary pore-forming toxins, PirA and PirB in a unique plasmid (pVA1) (7, 8). Horizontal transfer of plasmid (pVA1) is the main reason for the occurrence of AHPND (9). Vp_AHPND_ caused AHPND in shrimp showed a clinic sign including empty gut, pale and aqueous hepatopancreas, soft shell, which can lead to almost 100% mortality of shrimp (10, 11). Therefore, an effective controlling method to AHPND is urgently needed.

The outbreak of infectious disease is the interplay among pathogens, environment, and host (12). Genetic selective breeding of disease-resistant broodstock is regarded as a sustainable approach for disease control in aquaculture species (13). Several disease-resistant lines of *L. vannamei* against AHPND were established, but the detailed mechanism related to the AHPND resistance phenotype is largely unclear. With the development of high-throughput sequencing techniques and decoding of the *L. vannamei* genome (14), transcriptome sequencing has become an effective approach to discover genes related to disease control. Comparative transcriptome analysis showed that metabolism-related genes exhibited different expression pattern between AHPND-resistant and susceptible families (15 - 17), which suggested that metabolism might contribute to AHPND resistance phenotype. However, the expression pattern cannot accurately match the metabolic profile because of the processes between them including translation, post-translation modification, etc. As an effective method to investigate metabolite profiles and determine the physiological and biochemical status of a biosystem, metabolome analysis indicated that metabolic processes were greatly affected during pathogen infections in shrimp. The viral pathogen white spot syndrome virus (WSSV) could reprogram the metabolic progress, and the changed metabolites consequently modulate the antiviral immunity in shrimp (18 - 21). Relatively speaking, study on the metabolic changes during bacterial infection in shrimp is at the beginning. It was reported that *Vibrio* spp. infections could alter amino acid and carbohydrate metabolism in shrimp (22, 23). The bile acid metabolite taurocholate could induce biofilm formation and PirA/B toxin release of Vp_AHPND_ (24). Previous studies are mainly focused on the metabolic changes of shrimp responsive to Vp_AHPND_ infection; however, the metabolic differences and related molecular mechanism of shrimp resistant to AHPND infection are rarely investigated since limited genetic materials have been established till present.

Systematic genetic selection for the resistance trait to Vp_AHPND_ in *L. vannamei* was carried out in our previous work, and Vp_AHPND_ challenge test showed that significant resistance variations existed among different shrimp families (15). In the present study, comparative transcriptome and untargeted metabolome analyses were conducted to detect the differences between a pair of Vp_AHPND_-resistant and susceptible families under unchallenged or challenged conditions by Vp_AHPND_. The present study will provide useful information to clarify the underlying mechanism for the shrimp resistance to *Vibrio*.

## RESULTS

### Summaries of transcriptomic and metabolomic data for Vp_AHPND_-resistant and susceptible shrimp

**FIG 1:**
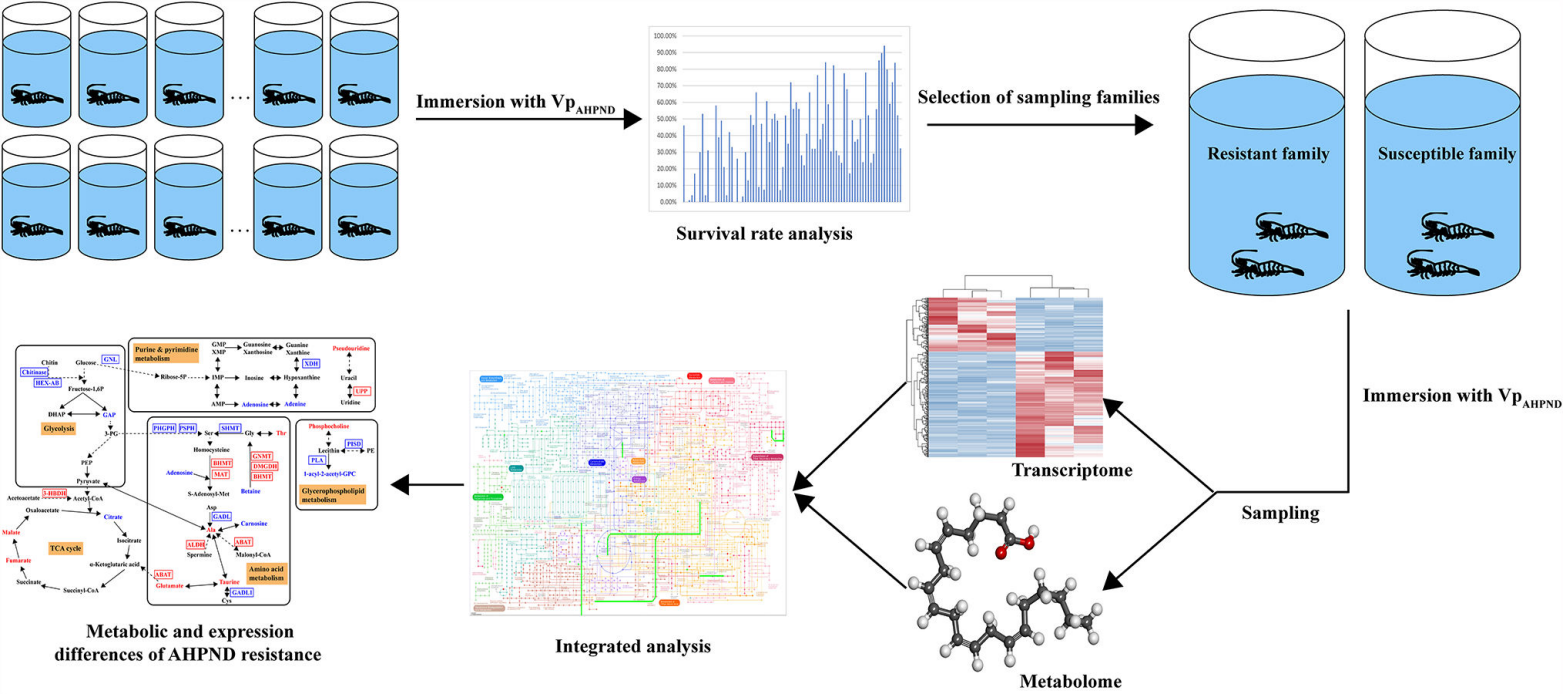
Schematic illustration about the experimental procedure. Different families were challenged by Vp_AHPND_ to evaluate their resistance trait to the pathogen. Considering the survival rate, growth stage, and pedigree information, a pair of Vp_AHPND_-resistant and susceptible families were selected and sampled for transcriptome sequencing and metabolome profiling. Integrated analysis of transcriptomic and metabolomic data identified the metabolic differences and related molecular mechanism between resistant and susceptible families.

A schematic illustration about the experimental procedure was shown in Fig. 1. Continuous immersion of Vp_AHPND_ was conducted previously to calculate the survival rates of multiple shrimp families. Considering the survival rate, growth stage, and pedigree information, a resistant family (R20523) and a susceptible family (S20507) were selected to conduct the comparative transcriptome and untargeted metabolome analyses. Integrated analysis of transcriptomic and metabolomic data uncovered the metabolic differences and related molecular mechanism between Vp_AHPND_-resistant and susceptible shrimp families. The survival rates of resistant family (R20523) and susceptible family (S20507) of shrimp were 84.31% and 3.85%, respectively (Fig. S1).

An overview of transcriptomic data was shown in Table S1. After filtering of low-quality reads, a total of 604,434,286 clean reads were obtained, including 287,643,206 reads from the susceptible family (S20507) and 316,791,080 reads from resistant family (R20523). Quality analysis showed that susceptible and resistant families had an average guanine-cytosine content of 45.32% and 44.63% of clean reads, a Q30 of 93.15% and 93.24%, and a map rate to the *L. vannamei* genome of 84.49% and 83.78%, respectively. Principal component analysis (PCA) was used to elucidate the expression patterns of different groups (Fig. S2). The data showed a high sequencing quality, indicating that the subsequent transcriptome analysis results were reliable. A total of 1,955 metabolites (1,561 in positive and 394 in negative ionization modes) were identified using the UHPLC-Q-TOF MS platform. The orthogonal partial least-squares discriminant analysis (OPLS-DA) model was used to elucidate different metabolic patterns for discrimination different groups (Fig. S3). The parameters (Table S2) indicated that the OPLS-DA model could appropriately describe the metabolomic data of Vp_AHPND_-resistant and susceptible shrimp, and the subsequent metabolome analysis results were reliable.

### Differentially expressed genes and metabolites between resistant and susceptible families under unchallenged condition

**FIG 2:**
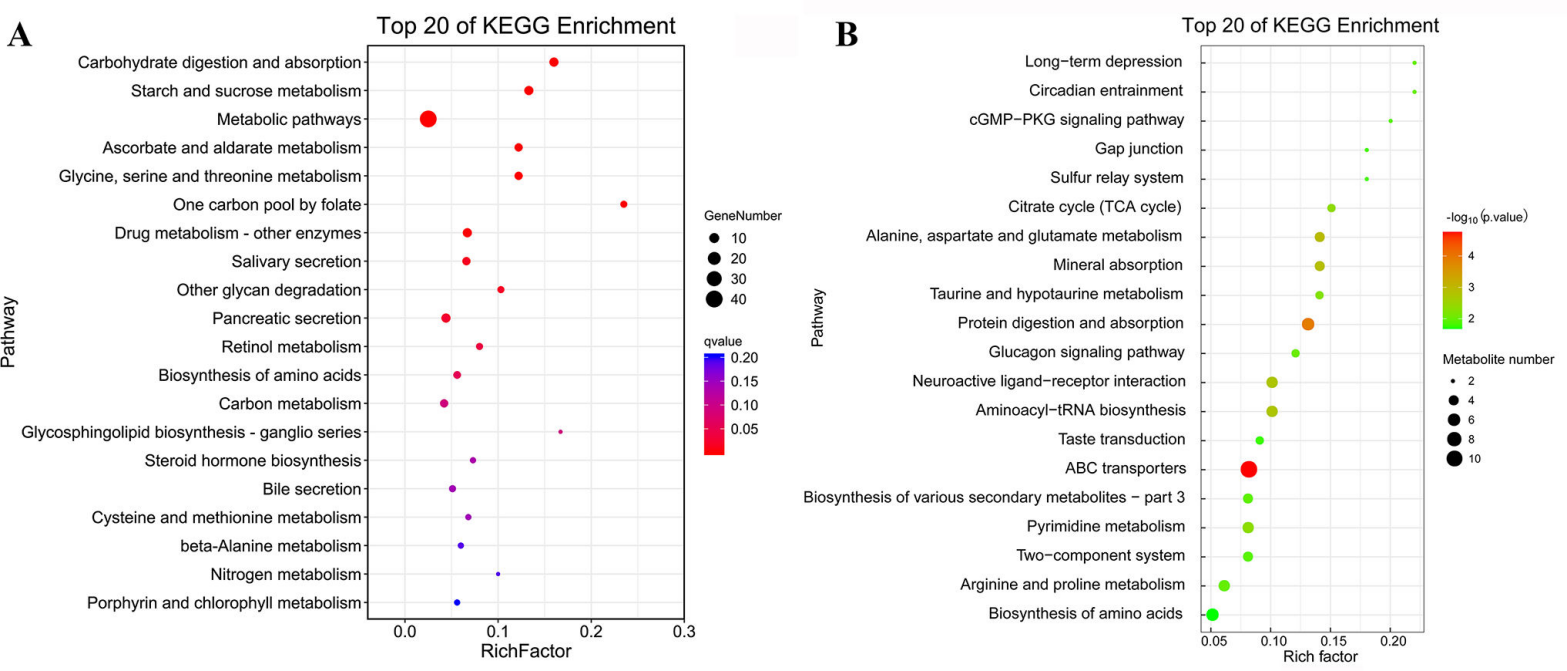
KEGG enrichment of DEGs and DMs between unchallenged R20523 and S20507 families (S20507-0h_vs_R20523-0h). (**A**) Scatterplot of the KEGG pathway enriched by DEGs between R20523 and S20507 families under unchallenged condition. (**B**) Scatterplot of the KEGG pathway enriched by DMs between R20523 and S20507 families under unchallenged condition. The vertical axis represents the name of the pathway and the horizontal axis shows the gene ratio. The size of the plot denotes the number of DEGs or DMs while the color corresponds to the Q-value or *P*-value. A deeper color represents a smaller Q-value or *P*-value and indicates a more significant pathway enrichment.

A total of 343 genes exhibited different expression profiles between R20523 and S20507 families at 0 hpi, in which 227 genes showed higher expressions in R20523 family, while 116 genes showed higher expressions in S20507 family (Table S3). Kyoto Encyclopedia of Genes and Genomes (KEGG) enrichment was performed to identify the function of differentially expressed genes (DEGs), and the significantly enriched pathways between these two families under unchallenged condition (S20507-0h_vs_R20523-0h) were listed in Fig. 2A. A total of 291 differential metabolites (DMs) (240 in positive and 51 in negative ionization modes) were identified between samples of unchallenged R20523 and S20507 families, and 58 metabolites showed higher content in R20523 family, and 233 metabolites showed higher content in S20507 family (Table S3). The enrichment analysis of DMs between these two families were listed in Fig. 2B. We surveyed the basal difference between susceptible and resistant families, and the shared KEGG pathways of transcriptome and metabolome between these two unchallenged families are shown in Fig. S4A.

**FIG 3:**
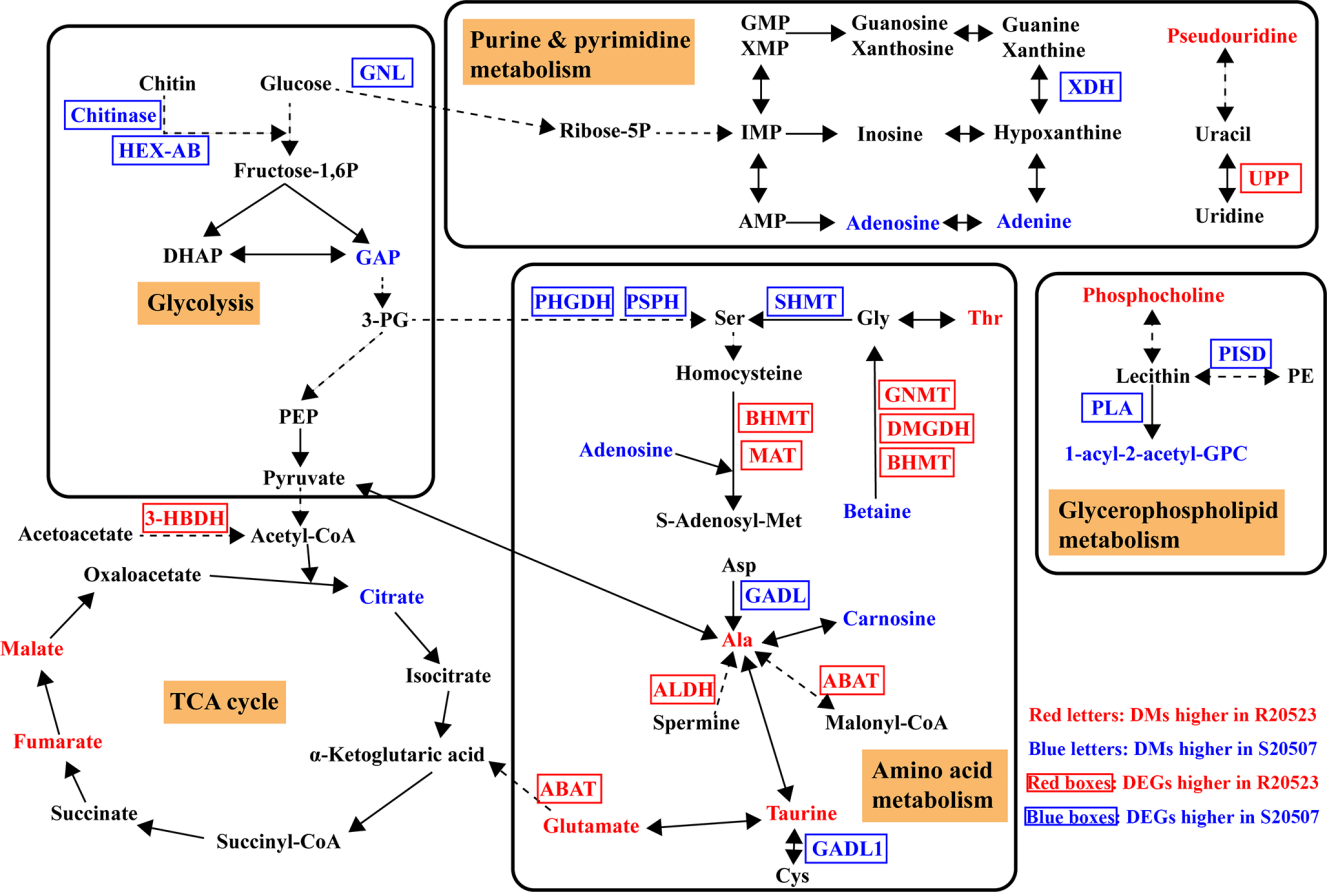
Overview of integrated analysis of DEGs and DMs between the unchallenged R20523 and S20507 families (S20507-0h_vs_R20523-0h). The differentially expressed gene abbreviations identified by the transcriptome analysis are shown in either blue (highly expressed in S20507 family) or red (highly expressed in R20523 family) boxes. Differentially abundant metabolites identified by the metabolome analysis are shown either in blue (highly expressed in S20507 family) or red (highly expressed in R20523 family) letters. Solid arrows represent a direct effect, and dotted arrows an indirect effect. HEX-AB, hexosaminidase; GNL, gluconolactonase; XDH, xanthine dehydrogenase; UPP, uridine phosphorylase; GAP, glyceraldehyde 3-phosphate; 3-HBDH, 3-hydroxybutyrate dehydrogenase; PHGDH, phosphoglycerate dehydrogenase; PSPH, phosphoserine phosphatase; SHMT, glycine hydroxymethyltransferase; BHMT, betaine-homocysteine S-methyltransferase; DMGDH, dimethylglycine dehydrogenase; GNMT, glycine N-methyltransferase; MAT, S-adenosylmethionine synthetase; ALDH, glutamate 5-kinase; ABAT, 4-aminobutyrate aminotransferase /(S)-3-amino-2-methylpropionate transaminase; GADL1, aspartate 1-decarboxylase; PISD, phosphatidylserine decarboxylase; PLA, secretory phospholipase A2.

The above identified DEGs and DMs were mapped to the KEGG pathway database and their links in metabolic pathways were obtained via the shared KEGG pathway. An overview of integrated analysis of DEGs and DMs identified between the Vp_AHPND_-resistant and susceptible families under unchallenged condition are shown in Fig. 3. Metabolic pathways including glycolysis, tricarboxylic acid (TCA) cycle, glycerophospholipid metabolism, purine metabolism, pyrimidine metabolism, and some amino acid metabolic pathways exhibited differences between them under unchallenged condition.

Integrated analysis showed that the intermediate product of glycolysis, glyceraldehyde 3-phosphate (GAP), and the intermediate metabolite of TCA cycle, citrate, showed higher levels in S20507 family than those in R20523 family. But the contents of malate and fumarate, the intermediate products of TCA cycle, were significantly lower in S20507 family. S20507 family also exhibited higher expression levels for chitinase and hexosaminidase (HEXA_B), which mediated the conversion of chitin and N-Acetylchitosamine. Moreover, the S20507 family had higher contents of adenosine and adenine, and higher expression levels of gluconolactonase (GNL) and xanthine dehydrogenase (XDH), which were involved in the purine and pyrimidine metabolism.

Moreover, many amino acid metabolic pathways were significantly enriched in the susceptible family S20507, including glycine, serine, and threonine metabolism; biosynthesis of amino acids; cysteine and methionine metabolism. In glycine, serine, and threonine metabolism, we observed higher gene expressions of phosphoglycerate dehydrogenase (PHGDH), phosphoserine phosphatase (PSPH), glycine hydroxymethyltransferase (SHMT), and lower content of threonine in S20507 family. Interestingly, S20507 family exhibited lower gene expressions of betaine-homocysteine S-methyltransferase (BHMT), dimethylglycine dehydrogenase (DMGDH), glycine N-methyltransferase (GNMT), and a higher content of betaine, which mediated the conversion of betaine and glycine. Similarly, the S20507 family had lower gene expressions of BHMT, S-adenosylmethionine synthetase (MAT), and higher content of adenosine, which mediated the conversion of homocysteine and S-adenosylmethionine in cysteine and methionine metabolism.

### Differentially expressed genes and metabolites between resistant and susceptible families after Vp_AHPND_ infection

**FIG 4:**
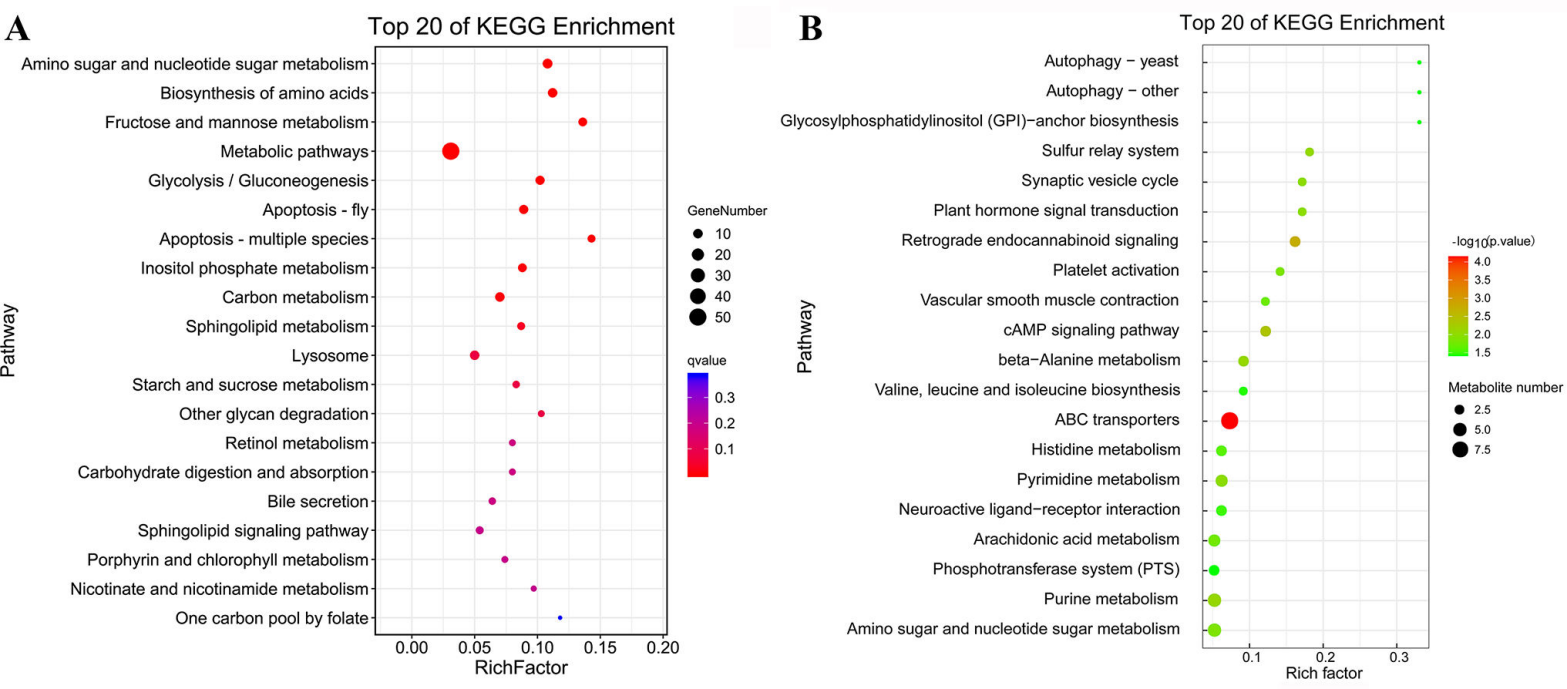
KEGG enrichment of DEGs and DMs between R20523 and S20507 families after Vp_AHPND_ infection (S20507-12h_vs_R20523-12h). (**A**) Scatterplot of the KEGG pathway enriched by DEGs between R20523 and S20507 families after Vp_AHPND_ infection. (**B**) Scatterplot of the KEGG pathway enriched by DMs between R20523 and S20507 families after Vp_AHPND_ infection. The vertical axis represents the name of the pathway and the horizontal axis shows the gene ratio. The size of the plot denotes the number of DEGs or DMs while the color corresponds to the Q-value or *P*-value. A deeper color represents a smaller Q-value or *P*-value and indicates a more significant pathway enrichment.

A total of 418 genes exhibited different expression profiles between R20523 and S20507 families at 12 hpi, in which 269 genes showed higher expressions in R20523 family, while 149 genes showed higher expressions in S20507 family after Vp_AHPND_ infection (Table S4). KEGG enrichment on these DEGs (S20507-12h_vs_R20523-12h) were listed in Fig. 4 A. On the other hand, a total of 195 DMs (158 in positive and 37 in negative ionization modes) were identified between the samples of Vp_AHPND_-challenged R20523 and S20507 families, and 48 metabolites showed higher content in R20523 family, and 147 metabolites showed higher content in S20507 family (Table S4). The enrichment analysis of DMs between these two families were shown in Fig. 4B.

**FIG 5:**
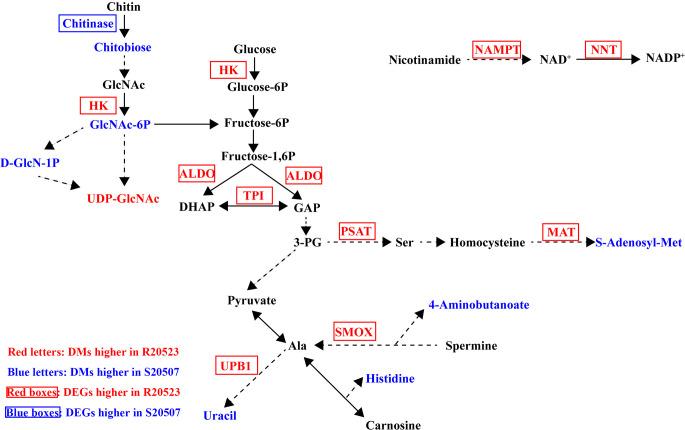
Overview of integrated analysis of DEGs and DMs between the R20523 and S20507 families after Vp_AHPND_ infection (S20507-12h_vs_R20523-12h). The differentially expressed gene abbreviations identified by the transcriptome analysis are shown in either blue (highly expressed in S20507 family) or red (highly expressed in R20523 family) boxes. Differentially abundant metabolites identified by the metabolome analysis are shown either in blue (highly expressed in S20507 family) or red (highly expressed in R20523 family) letters. Solid arrows represent a direct effect, and dotted arrows an indirect effect. HK, Hexokinase; TPI, triose-phosphate isomerase; ALDO, fructose-bisphosphate aldolase; PSAT, phosphoserine aminotransferase; MAT, S-adenosylmethionine synthetase; SMOX, spermine oxidase; UPB1, beta-ureidopropionase. NAMPT, nicotinamide phosphoribosyltransferase; NNT, nicotinamide nucleotide transhydrogenase; DHAP, dihydroxyacetone phosphate; GAP, glyceraldehyde 3-phosphate.

The shared KEGG pathways of transcriptomic and metabolomic data (Fig. S4B) are used to draw an overview of integrated analysis of DEGs and DMs identified between the Vp_AHPND_-resistant and susceptible families after Vp_AHPND_ infection (Fig. 5). After Vp_AHPND_ infection, genes in glycolysis, including HK, TPI, and ALDO, and some genes involved in amino acid metabolism, including PAST, MAT, SMOX, and UPB1, showed higher expressions in R20523 family. But the content of most DMs like S-Adenosyl-Met, 4-aminobutanoate, histidine, uracil, and chitobiose were higher in S20507 family.

### Differentially expressed genes and metabolites between resistant and susceptible families with or without infection

**FIG 6:**
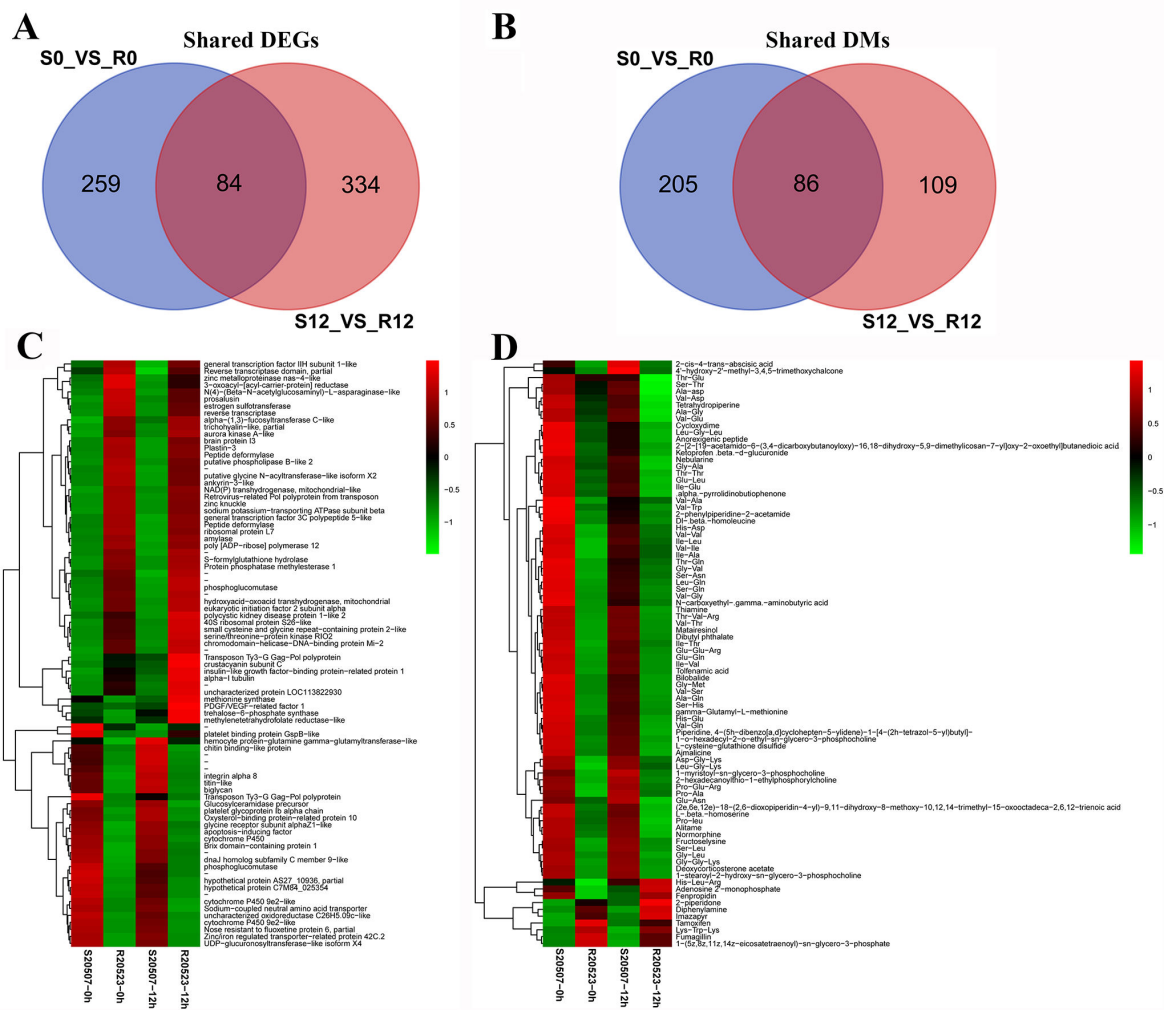
Common DEGs and DMs between resistant and susceptible families under both unchallenged and challenged conditions. (**A**) Venn map of DEGs between resistant and susceptible families under both unchallenged and challenged conditions. (**B**) Venn map of DMs between resistant and susceptible families under both unchallenged and challenged conditions. (**C**) Heatmap of DEGs between resistant and susceptible families under both unchallenged and challenged conditions. (**D**) Heatmap of DMs between resistant and susceptible families under both unchallenged and challenged conditions.

Venn diagram for DEGs and DMs between resistant and susceptible families under both normal and infected conditions were shown in Fig. 6A and B, respectively. Further analysis on 84 common DEGs showed that 30 DEGs had higher expression levels and 48 DEGs presented lower expression levels in susceptible family under both conditions. Six DEGs showed higher expression levels in susceptible family under normal condition but exhibited opposite expression profiles after Vp_AHPND_ infection (Fig. 6C). Among the 30 DEGs highly expressed in susceptible family, 24 genes had annotations, including cytochrome P450, apoptosis-inducing factor, chitin-binding protein, integrin alpha 8, platelet glycoprotein, phosphoglucomutase. The 48 DEGs with lower expressions in susceptible family included general transcription factor ⅡH, general transcription factor 3C, amylase, eukaryotic initiation factor two subunit alpha, prosalusin, peptide deformylase, poly [ADP-ribose] polymerase 12, crustacyanin subunit C, insulin-like growth factor-binding protein-related protein, serine/threonine-protein kinase RIO2. Six DEGs with opposite expression profiles before and after infection were platelet binding protein GspB, methylenetetrahydrofolate reductase, trehalose-6-phosphate synthase, methionine synthase, and PDGF/VEGF-related factor 1.

Analysis of 86 common DMs showed that 76 DMs had higher content and seven DMs had lower content in susceptible family under both conditions (Fig. 6D). Among the 76 DMs with higher content in S20507 family, most of them were dipeptides and tripeptides, and other DMs included 2-cis-4-trans-abscisic acid, tetrahydropiperine, anorexigenic peptide, nebularine, thiamine, L-beta-homoserine, fructoselysine. Seven DMs with lower content in susceptible family were diphenylamine, fumagillin, tamoxifen, 2-piperidone, imazapyr, Lys-Trp-Lys, and 1-stearoyl-2-hydroxy-sn-glycero-3-phosphocholine. While, three DMs including adenosine 2'-monophosphate (AMP), fenpropidin, and tripeptide His-Leu-Arg, had higher content in susceptible family under normal condition but lower content after Vp_AHPND_ infection.

### Different transcriptomic and metabolic responses to Vp_AHPND_ infection in resistant and susceptible families

**FIG 7:**
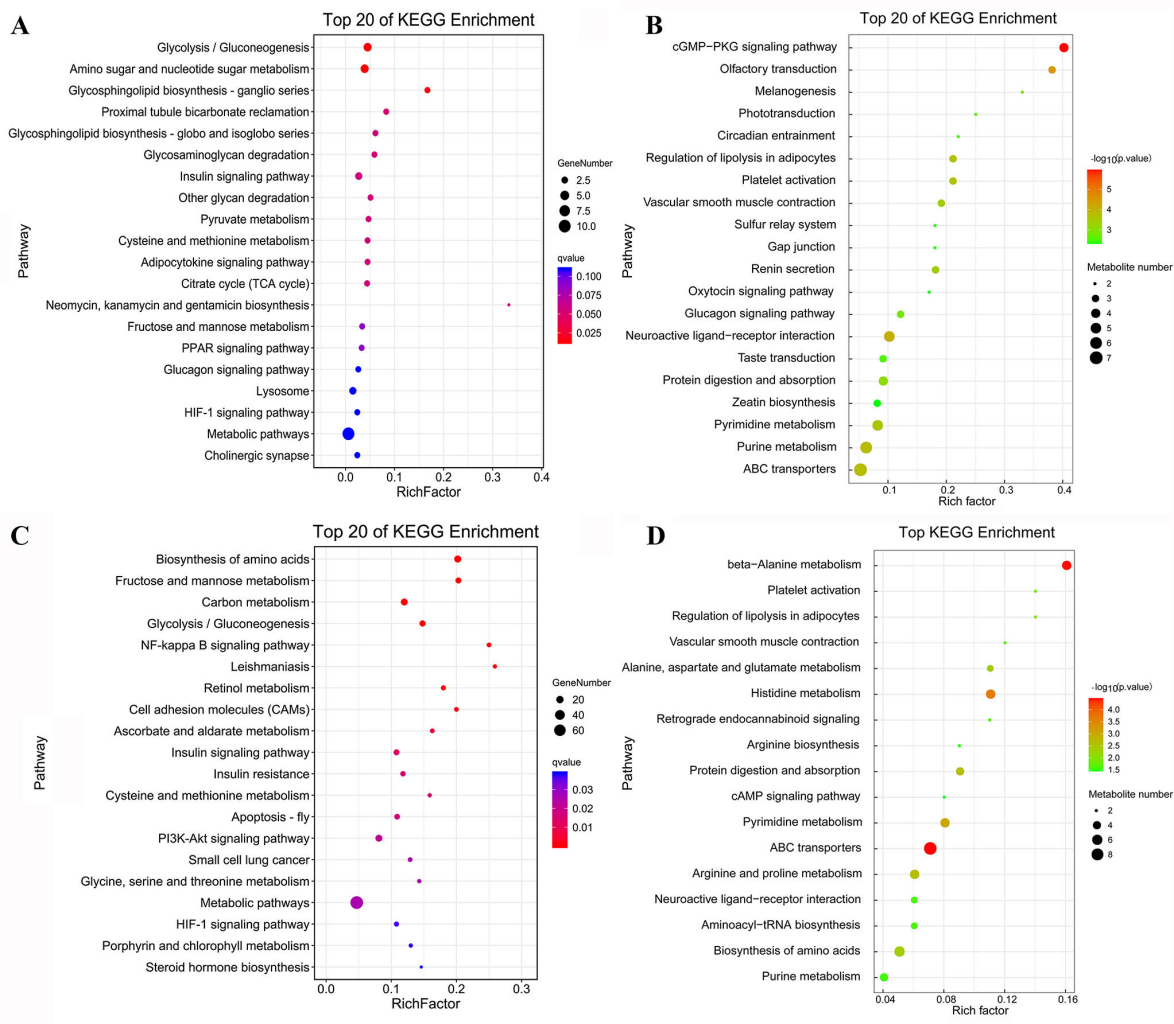
KEGG enrichment of DEGs and DMs responded to Vp_AHPND_ infection in R20523 and S20507 families. (**A**) Scatterplot of the KEGG pathway enriched by DEGs responded to Vp_AHPND_ infection in S20507 family. (**B**) Scatterplot of the KEGG pathway enriched by DMs responded to Vp_AHPND_ infection in S20507 family. (**C**) Scatterplot of the KEGG pathway enriched by DEGs responded to Vp_AHPND_ infection in R20523 family. (**D**) Scatterplot of the KEGG pathway enriched by DMs responded to Vp_AHPND_ infection in R20523 family. The vertical axis represents the name of the pathway and the horizontal axis shows the gene ratio. The size of the plot denotes the number of DEGs or DMs while the color corresponds to the Q-value or *P*-value. A deeper color represents a smaller Q-value or *P*-value and indicates a more significant pathway enrichment.

A total of 149 DEGs and 106 DMs responsive to Vp_AHPND_ infection were identified in S20507 family (Table S5), and the enriched KEGG pathways were presented in Fig. 7A and B. However, a total of 800 DEGs and 146 DMs were identified in R20523 family after Vp_AHPND_ infection (Table S6), and their enriched KEGG pathways were given in Fig. 7C and D. Integrated analysis of transcriptomic and metabolomic data were conducted based on the shared KEGG pathways of DEGs and DMs (Fig. S4C and D), and the result showed that the metabolic flow of carbohydrates and amino acids was reprogrammed in S20507 family during Vp_AHPND_ infection process, but arachidonic acid metabolism and cAMP signaling pathway were activated in the R20523 family after Vp_AHPND_ infection.

**FIG 8:**
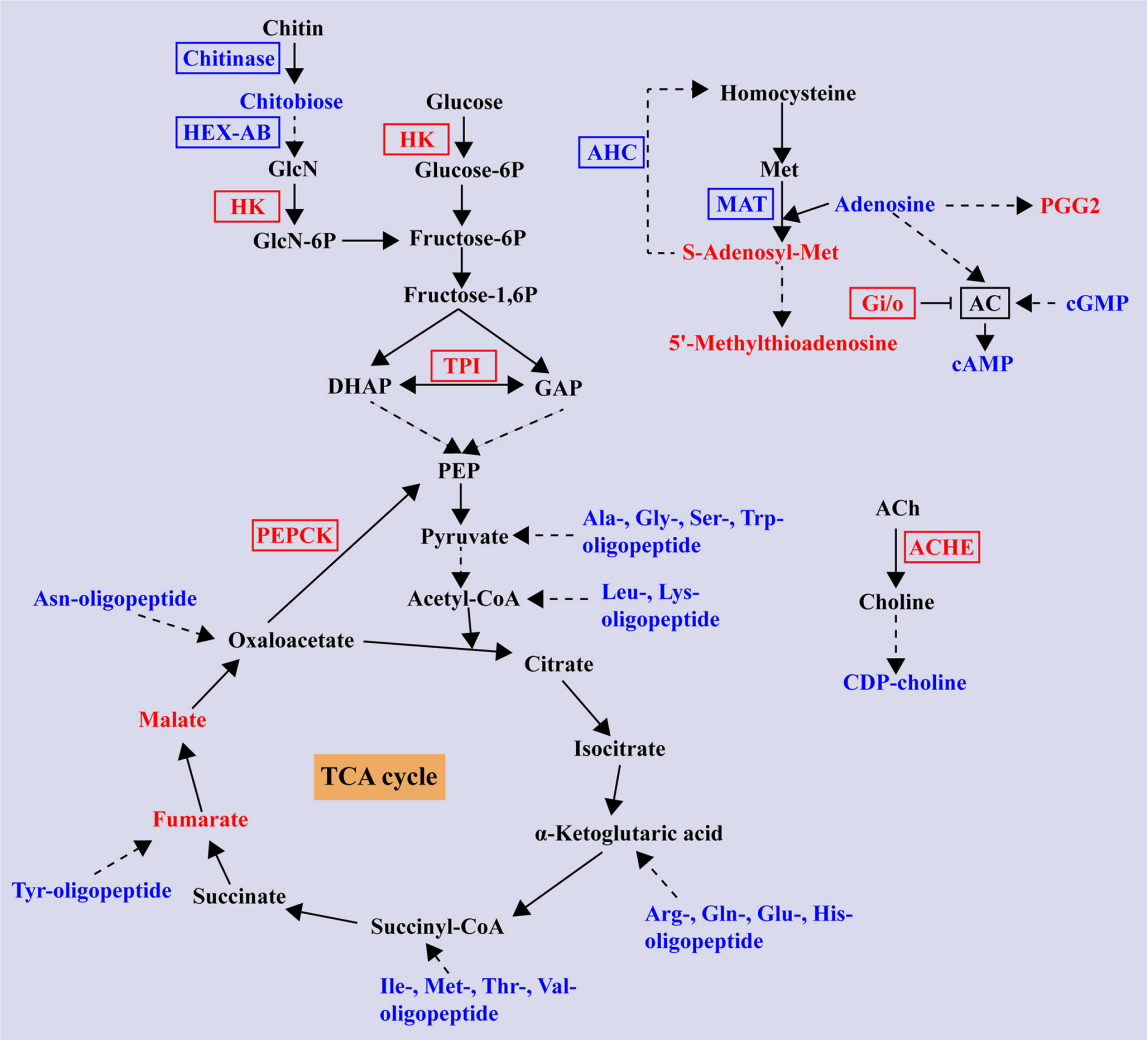
Overview of integrated analysis of DEGs and DMs responded to Vp_AHPND_ infection in S20507 family (S20507-0h_vs_S20507-12h). The differentially expressed gene abbreviations identified by the transcriptome analysis are shown in either blue (down-regulated DEGs) or red (up-regulated DEGs) boxes. Differentially abundant metabolites identified by the metabolome analysis are shown either in blue (decreased upon infection) or red (increased upon infection) letters. Solid arrows represent a direct effect, and dotted arrows an indirect effect. HEX-AB, hexosaminidase; HK, hexokinase; TPI, triose-phosphate isomerase; PEPCK, phosphoenolpyruvate carboxykinase; ACHE, acetylcholinesterase; G_i/o_, guanine nucleotide-binding protein subunit gamma; AHC, adenosylhomocysteinase; MAT, S-adenosylmethionine synthetase; GAP, glyceraldehyde 3-phosphate; AC, adenylate cyclase.

An overview of integrated analysis of DEGs and DMs identified in S20507 family after Vp_AHPND_ infection are shown in Fig. 8. In the S20507 family, the Vp_AHPND_ infection significantly up-regulated the expression of HK and TPI in glycolysis, and phosphoenolpyruvate carboxykinase (PEPCK) in gluconeogenesis. Meanwhile, the metabolomic data showed that malate and fumarate, which are intermediate products of TCA cycle, were boosted after Vp_AHPND_ infection. It is worthy to note that 33 dipeptides and tripeptides showed significant difference in S20507 family, and 31 of them were down-regulated after Vp_AHPND_ infection. These down-regulated oligopeptides were composed of the following amino acids, including Ala, Gly, Ser, Trp, Arg, Gln, Glu, His, Ile, etc., and most of them were glycogenic amino acids, which could be converted to carbohydrate via TCA cycle.

**Fig 9:**
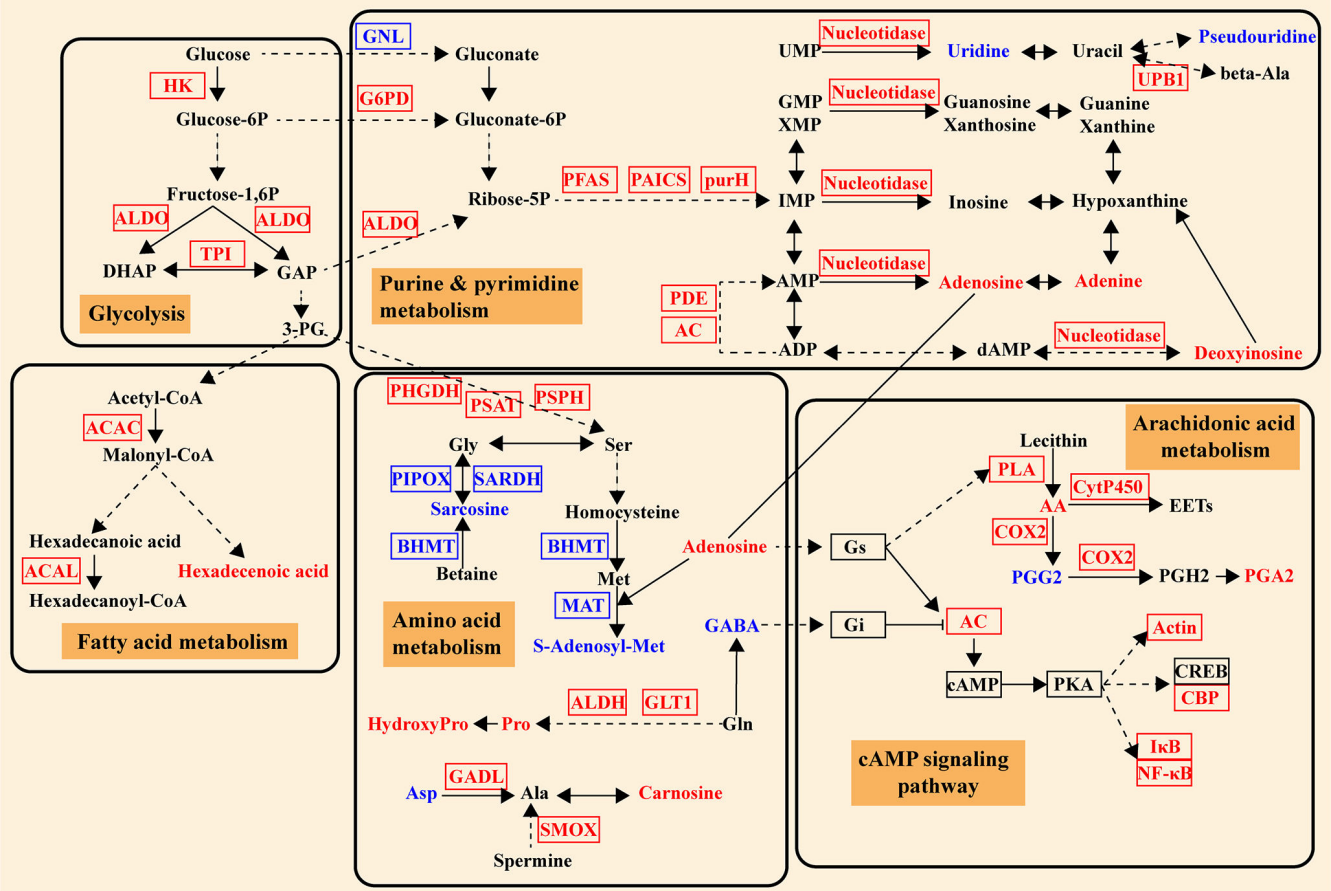
Overview of integrated analysis of DEGs and DMs responded to Vp_AHPND_ infection in R20523 family (R20523-0h_vs_R20523-12h). The differentially expressed gene abbreviations identified by the transcriptome analysis are shown in either blue (down-regulated DEGs) or red (up-regulated DEGs) boxes. Differentially abundant metabolites identified by the metabolome analysis are shown either in blue (decreased upon infection) or red (increased upon infection) letters. Solid arrows represent a direct effect, and dotted arrows an indirect effect. HK, Hexokinase; TPI, triose-phosphate isomerase; ALDO, fructose-bisphosphate aldolase; ACAC, acetyl-CoA carboxylase; ACAL, long-chain acyl-CoA synthetase; GNL, gluconolactonase; G6PD, glucose-6-phosphate 1-dehydrogenase; purH, IMP cyclohydrolase; PAICS, phosphoribosylaminoimidazole-succinocarboxamide synthase; AC, adenylate cyclase; PFAS, phosphoribosylformylglycinamidine synthase; UPB1, beta-ureidopropionase; PDE, cAMP-specific phosphodiesterase; 3-PG, 3-phosphoglycerate; PHGDH, phosphoglycerate dehydrogenase; PSAT, phosphoserine aminotransferase; PSPH, phosphoserine phosphatase; PIPOX, sarcosine oxidase; SARDH, sarcosine dehydrogenase; BHMT, betaine-homocysteine S-methyltransferase; MAT, S-adenosylmethionine synthetase; ALDH, glutamate 5-kinase; GADL1, aspartate 1-decarboxylase; GLT1, glutamate synthase; SMOX, spermine oxidase; GABA, 4-aminobutanoic acid; PLA, phospholipase A; COX2, cyclooxygenase-2; CytP450, cytochrome P450; AA, arachidonic acid; PGA2, prostaglandin a2; PGG2, prostaglandin g2; IκB, cactus; NF-κB, relish; CREB, cAMP-response element binding protein; CBP, CREB-binding protein.

Different from the S20507 family, integrated analysis of DEGs and DMs showed that distinct metabolic responses happened in R20523 family after Vp_AHPND_ infection (Fig. 9) . Although Vp_AHPND_ infection up-regulated the expression of genes involved in glycolysis, including HK, TPI, and ALDO, the metabolic flow seemed to be turned into metabolism of purine, pyrimidine, and amino acid in R20523 family, rather than into TCA cycle. In R20523 family, Vp_AHPND_ infection up-regulated the expression of glucose-6-phosphate 1-dehydrogenase (G6PD), phosphoribosylformylglycinamidine synthase (PFAS), ALDO, phosphoribosylaminoimidazole-succinocarboxamide synthase (PAICS), IMP cyclohydrolase (purH), adenylate cyclase (AC), 5'-nucleotidase, beta-ureidopropionase (UPB1), and cAMP-specific phosphodiesterase (PDE), and increased the content of adenosine, adenine, deoxyinosine for purine and pyrimidine metabolism. In glycine, serine, and threonine metabolism, the expression of PHGDH, phosphoserine aminotransferase (PSAT) and PSPH, which converted the glycolytic intermediate 3-phosphoglycerate (3-PG) to serine, were activated after infection. While, the expression of sarcosine oxidase (PIPOX), sarcosine dehydrogenase (SARDH), S-adenosylmethionine synthetase (MAT) and BHMT, which mediated the conversion between betaine and glycine, as well as its intermediate metabolites, sarcosine and S-adenosylmethionine showed decrease in R20523 family after infection. These changes showed certain similarity with the differences between S20507 and R20523 families without Vp_AHPND_ infection.

In addition, some innate immunity related pathways including arachidonic acid metabolism and cAMP signaling pathway were also activated in R20523 family (Fig. 9). As for the metabolites of purine metabolism and amino acid metabolism, the content of adenosine was induced and the content of gamma-aminobutyric acid (GABA) was reduced after Vp_AHPND_ infection, which could consequently modulate the arachidonic acid metabolism and cAMP signaling pathway. The expression of phospholipase A (PLA), cyclooxygenase-2 (COX2), cytochrome P450 (CytP450) in arachidonic acid metabolism were up-regulated after infection. In this pathway, the content of arachidonic acid (AA), and prostaglandin a2 (PGA2) increased, while the content of prostaglandin g2 (PGG2) decreased after infection. In the cAMP signaling pathway, the expression of AC, beta-actin, cactus (IκB), relish (NF-κB), and CREB-binding protein (CBP) were activated after Vp_AHPND_ infection.

### Confirmation of transcriptome sequencing data via quantitative real-time PCR

**FIG 10:**
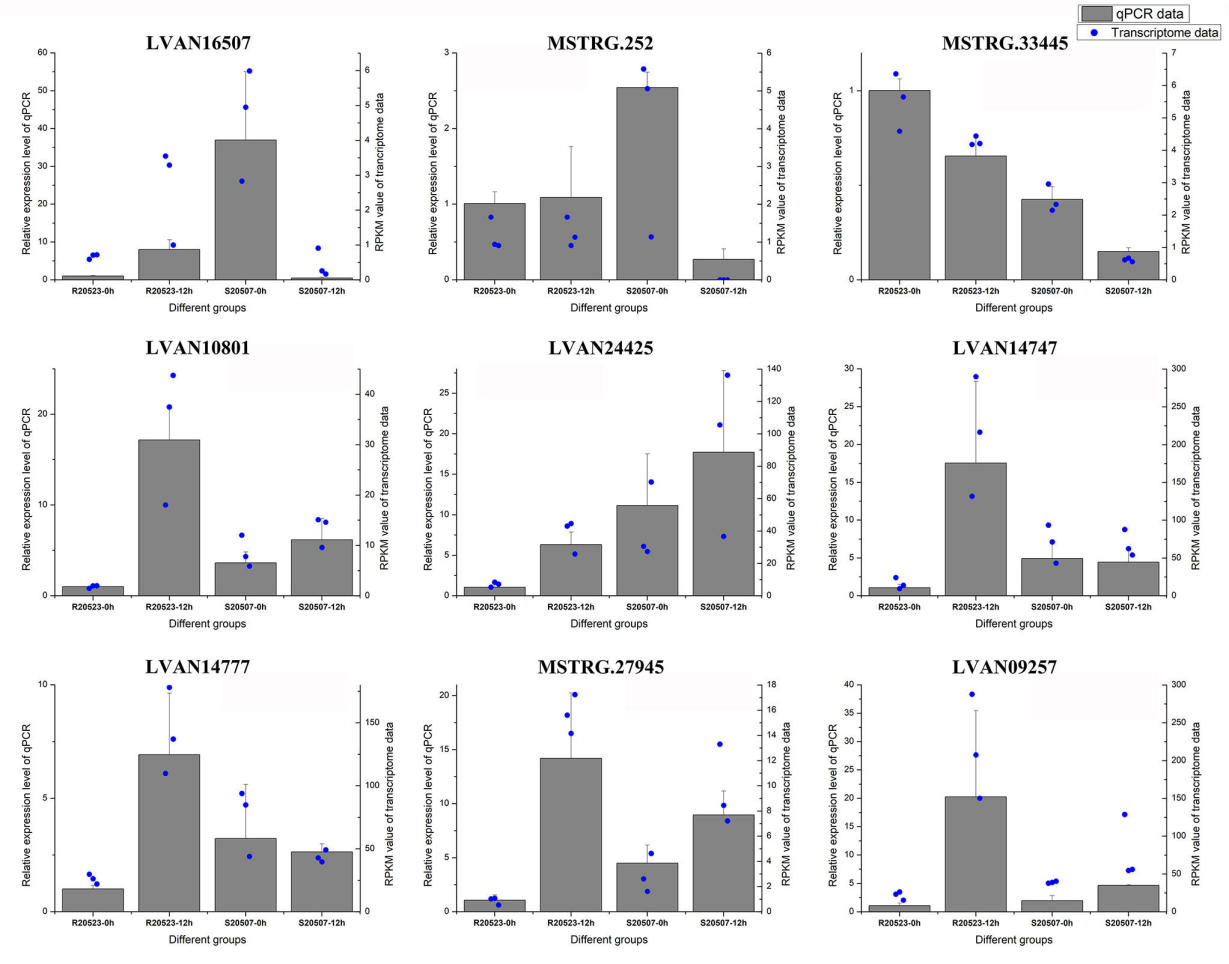
Evaluation of the transcriptome results by quantitative real-time PCR. The expression levels of these genes were presented as mean ± SD and shown in the black bar chart. The gene 18S rRNA was used as the reference gene. The expression levels were all obtained from three biological replicates. The RPKM values of these genes from transcriptomic data were shown in the blue scatter plots, and each plot represented the value of one biological replicate. LVAN16507, platelet binding protein GspB; MSTRG.252, no functional annotation; MSTRG.33445, reverse transcriptase; LVAN10801, trehalose-6-phosphate synthase; LVAN24425, hemocyte protein-glutamine gamma-glutamyltransferase; LVAN14747, methylenetetrahydrofolate reductase; LVAN14777, methionine synthase; MSTRG.27945, monocarboxylate transporter 12; LVAN09257, hexokinase.

The quantitative real-time PCR (qPCR) was conducted to verify the transcriptomic data and the results of nine selected genes were shown in Fig. 10. The expression profiles of these DEGs were consistent with the transcriptomic sequencing results, suggesting that the transcriptome analysis data were reliable.

## DISCUSSION

Vp_AHPND_ is a severe bacterial pathogen of cultured shrimp, and its infection induced dramatic metabolic changes (22, 24). Disease resistance is closely related to the host physiology and health state, and previous studies indicated that resistance against Vp_AHPND_ was a hereditable trait in shrimp (25). Integrated analysis of transcriptomic and metabolomic data showed that several metabolic pathways including serine-glycine metabolism, betaine-homocysteine metabolism, purine metabolism, pyrimidine metabolism, arachidonic acid metabolism, TCA cycle, and gluconeogenesis exhibited significant difference between Vp_AHPND_-resistant and susceptible families under or without Vp_AHPND_ infection conditions. These data showed that the differences in these metabolism pathways might contribute to the disease resistance of shrimp to bacteria.

Serine is a proteinogenic amino acid and it can be *de novo* synthesized from glycolytic or gluconeogenic intermediate 3-phosphoglycerate through a series of enzymes including PHGDH, PAST, and PSPH (26). Along with producing glycine via SHMT, serine donates one-carbon units to the folate cycle, which is essential for *de novo* synthesis of adenosine, guanosine and thymidylate, and glycine can not only provide one-carbon units to the folate cycle, but also function as a precursor for the synthesis of purine and glutathione (27). Besides providing one-carbon units and glycine for purine synthesis via serine catabolism, ATP synthesized in this way also contributes adenosine for the production of SAM from methionine, which participates in a methylation reaction through the SAM, S-adenosylhomocysteine (SAH), homocysteine and methionine cycle (28). A methylation of DNA and RNA can regulate gene expression or translation (29). Proteins themselves can be post-translationally modified by methylation, which can alter function and protein-protein interactions. Metabolite SAM is required for methylation of DNA and histones, and dietary reduction of methionine decreases SAM levels, leading to diminished histone methylation with significant effects upon gene expression (30). In the present study, Vp_AHPND_ infection significantly induced serine and glycine synthesis, pentose phosphate pathway, as well as purine and pyrimidine metabolism, but reduced betaine-homocysteine metabolism, which might be used as source for bacterial proliferation. Similarly, the susceptible family without infection also exhibited a higher level of serine and glycine synthesis, as well as purine and pyrimidine metabolism, while a lower level of SAM-homocysteine-methionine cycle mediated methylation reaction, suggesting susceptible family exhibited a relative prosperous state of expression, which might be hijacked by the pathogens for its propagation.

Arachidonic acid is an integral constituent of biological cell membrane for all cells, and its metabolites have a considerable role in maintaining the healthy state of animals (31). In mammals, arachidonic acid could activate NF-κB through inhibition of NO release at early stages of the inflammatory process (32). Adenosine induced cyclooxygenase (COX-2) could respond to inflammation stimuli and then catalyze arachidonic acid into prostaglandins (PGs) to modulate the intestinal barriers (33, 34). In *L. vannamei*, dietary arachidonic acid could modulate the expressions of immune genes, such as caspase-3, cytochrome C, and increase the relative abundances of intestinal beneficial bacteria for resistance to microcystin-LR stress (35). In the present study, metabolites and related enzymes in the arachidonic acid metabolism were boosted after Vp_AHPND_ infection in the resistant family. In *Apostichopus japonicus*, arachidonic acid metabolism could be increased by *V. splendens* infection and elevated the immune responses of sea cucumber (36). In addition to arachidonic acid metabolism, several immune regulatory genes including AC, beta-actin, CBP and relish, were also activated in the resistant family after Vp_AHPND_ infection. CBP could act as a coactivator and augment the activity of phosphorylated CREB to activate transcription of cAMP-responsive genes (37). Relish, an essential NF-κB transcription factor in shrimp, can be activated via upstream IMD signaling to regulate the expression of various antimicrobial peptides (AMPs) that directly kill foreign pathogens (38, 39). Similarly, the expressions of several antilipopolysaccharide factors were also up-regulated after Vp_AHPND_ infection in resistant family (data not shown). *Vibrio cholerae* infection to human increased the synthesis of cAMP, and cAMP with its receptor protein negatively regulated the coordinate expression of cholera toxin and toxin-coregulated pilus in *V. cholerae* (40, 41). These results suggested that the activated arachidonic acid metabolism and some immune pathways might contribute to the resistance of shrimp to AHPND.

TCA cycle is a central hub of carbon metabolism coordinating the metabolism of glucose, amino acids, and biosynthetic pathways such as lipogenesis and nucleic acid synthesis (42). Phosphoenolpyruvate carboxykinase (PEPCK) is the rate-limiting enzyme of gluconeogenesis and a key regulator of TCA cycle flux (43). Following the conversion of amino acids and other noncarbohydrate sources to oxaloacetate (OAA) in the TCA cycle, PEPCK catalyzes the conversion of OAA into phosphoenolpyruvate (PEP), and then PEP is converted to glucose or other molecules depending cellular needs (44). Under glucose starvation condition, cancer cells increase their utilization of glutamine or other nutrient source for the synthesis of ribose (42, 45). During Vp_AHPND_ infection, the expression of PEPCK and the content of its product PEP were up-regulated in *L. vannamei* (22, 46). Previous study also revealed the increase of intermediates in TCA cycle (cis-aconitate, citrate, fumarate, isocitrate, and succinate) and decrease of several amino acids in Vp_AHPND_ challenged shrimp compared to controls (22). Similarly, in the present study, we also observed the increase of intermediates in TCA cycle (fumarate and malate) and up-regulated expression of PEPCK in the susceptible family. Moreover, 31 of the 33 differential dipeptides and tripeptides showed significant decrease after infection in S20507 family. Our previous results showed that nearly four-fifths of differential metabolites at 24 hpi were dipeptides and most of them were down-regulated compared with those at 0 hpi (data not shown). These results suggested that the susceptible family reprogrammed the metabolism flux, and extensively utilized amino acids or other noncarbohydrate sources via TCA cycle after Vp_AHPND_ infection, which might provide materials for the proliferation and growth of Vp_AHPND_.

WSSV infection enhanced aerobic glycolysis and nucleotide biosynthesis to achieve successful replication by altering the host metabolism (18). Metabolites induced by WSSV infection could modulate the antiviral immunity in shrimp. The metabolite linoleic acid could inhibit WSSV proliferation through direct binding to virions and then activating shrimp ERK-NF-κB signaling pathway mediated production of antimicrobial peptide (21). Although the pentose phosphate pathway and nucleotide biosynthesis were also boosted by Vp_AHPND_ infection (47), the Vp_AHPND_ induced metabolic changes were different from those influenced by WSSV infection. For example, glycine, serine and threonine metabolism, and gluconeogenesis were activated by Vp_AHPND_ but not WSSV infection (22, 47). Metabolites induced by Vp_AHPND_ infection could also affect the infectious process. It was reported that the metabolite taurine, which was up-regulated by Vp_AHPND_ infection, enhanced penaeid shrimp’s antibacterial response and survival to this pathogenic *Vibrio* infection (48). Curiously, resistant family showed higher content of taurine in comparison with that in susceptible family in the present study. In addition, the intestinal microbiome is a pivotal and direct regulator of animal physiology, immunity, and health (49). Intestinal microbiome-mediated resistance against vibriosis was reported in fish aquaculture (50). Between the resistant and susceptible shrimp, several common DMs showed both different content with or without Vp_AHPND_ infection, but they seemed to be not synthesized by host. The relevance of the microbiome and their metabolites to host might be an interesting issue for further investigation.

In summary, comparative transcriptome and untargeted metabolome of hepatopancreas between genetically selected susceptible family and resistant family of shrimp against AHPND showed that metabolism difference in hepatopancreas might contribute greatly to the resistance differences between families. Susceptible family showed a relative prosperous state of metabolism and Vp_AHPND_ might hijack them as source for bacterial proliferation. After Vp_AHPND_ infection, activation of NF-κB and cAMP pathways induced by arachidonic acid metabolism contributed to the resistance to AHPND in the resistant family. Conversely, the amino acid catabolism boosted via reprogrammed PEPCK-mediated TCA cycle flux increased and the homeostasis of hepatopancreas was more easily collapsed in susceptible family after Vp_AHPND_ infection. These data provided important information for clarifying the mechanism of shrimp resistance to AHPND, and these information could be used for accelerating the genetic breeding in shrimp aquaculture.

## MATERIALS AND METHODS

### Experimental animals, *V. parahaemolyticus* challenge, and sample collection

The full-sib families of shrimp were produced and stocked separately in Bohai Seafood Breeding (Hainan) Co., Ltd, Wenchang, China. Multiple families were challenged by Vp_AHPND_ each year to evaluate their resistance trait to AHPND, and the families with high survival rate were mated to generate the next-generation families. The Vp_AHPND_ (AG01) were isolated from diseased shrimp in our lab as described by Liu et al. (51), and identified as *V. parahaemolyticus*. Specific primers of PirA and PirB toxins were used to detect the pVA1 plasmid which mainly causes the occurrence of AHPND. Vp_AHPND_ was prepared according to the method described by Zhang et al. (52), and different families were challenged with the cultured Vp_AHPND_ via immersion infection under a final concentration of 5 × 10^6^ CFU/mL. Dead shrimp was collected every 4 h, and the survival rate was calculated after 48 h continuous immersion infection.

Considering the survival rate, growth stage, and pedigree information, a pair of resistant and susceptible families were selected for transcriptome sequencing and metabolome profiling. Healthy shrimp from resistant family (R20523), with the average body length of 2.65 cm, and susceptible family (S20507), with the average body length of 2.73 cm, were immersed with Vp_AHPND_ at the final concentration of 2.5 × 10^6^ CFU/mL. For each family, the hepatopancreas of 27 individuals were collected at 0 and 12 hpi, respectively. Four-fifths of each hepatopancreas from three individuals were mixed together as one metabolome sample, and the rest part of the hepatopancreas collected from nine individuals were mixed together as one transcriptome sample, so each family contained nine biological replicates in metabolome profiling and three biological replicates in transcriptome sequencing. All samples were rapidly frozen in liquid nitrogen and stored at −80°C for further transcriptome sequencing and metabolome profiling.

### RNA extraction, sequencing, and bioinformatics analysis

Total RNA of the hepatopancreas was extracted using RNAiso Plus (Takara, Japan) for RNA sequencing by Gene Denovo Biotechnology Co. Ltd., Guangzhou, China. RNA quality was assessed on an Agilent 2100 Bioanalyzer (Agilent Technologies, Palo Alto, CA, USA) and checked using RNase free agarose gel electrophoresis. The mRNA was enriched using Oligo (dT) beads from extracted total RNA, and the enriched RNA was fragmented into short fragments using fragmentation buffer and reverse transcripted into cDNA with random primers. Then the second-strand cDNA were synthesized, and these fragments were purified with QiaQuick PCR extraction kit. After end repair and the addition of poly (A), the short fragments were ligated with sequencing adapters, size selected by agarose gel electrophoresis, enriched by PCR amplification, and sequenced using Illumina Novaseq6000.

The paired-end clean reads were mapped to the shrimp genome (https://www.shrimpbase.net/vannamei.html) using HISAT v2.2.4 with “-rna-strandness RF” and other parameters set as a default. The mapped reads of each sample were assembled by using StringTie v1.3.1 in a reference-based approach. RSEM software was used to calculate FPKM (fragment per kilobase of transcript per million mapped reads) value to quantify its expression abundance and variations.

### Analysis of differentially expressed genes

PCA was performed with R package gmodels (http://www.r-project.org/) to reveal the relationship of the samples. Differential expression analysis was performed by DESeq2 software between two different groups. Genes with false discovery rate (FDR) <0.01 and absolute fold change |FC| ≥2 were identified as differentially expressed genes (DEGs). The DEGs were enriched by Gene Ontology (GO) and KEGG. GO is an international standardized gene function classification system consisting of terms that use controlled vocabulary to provide a comprehensive representation of gene function. KEGG is a major public pathway-related database that can identify significantly enriched metabolic pathways in DEGs from the background of the whole genome.

### Evaluation of the transcriptome results by qPCR

qPCR was conducted to verify the accuracy of the expression profiles obtained from the transcriptome analysis. Briefly, total RNA was used to synthesize the cDNA template using the PrimeScript RT reagent kit (Takara, Japan) with random primers, and qPCR was performed using Thunderbird SYBR qPCR mix (Toyobo, Osaka, Japan) to quantify the mRNA expression levels. Nine DEGs whose expression levels showed significant differences among at least three comparisons were selected for verification. The primers used for qPCR analysis are listed in Table 1. The 18S rRNA (GenBank No. EU920969) was employed as an internal control for cDNA normalization. The PCR product was denatured to produce a melting curve to check the specificity of the PCR product.

**TABLE 1:** Primers for evaluation of the transcriptome results by quantitative real-time PCR

Name	Sequence (5’−3’)
18S-qF	TATACGCTAGTGGAGCTGGAA
18S-qR	GGGGAGGTAGTGACGAAAAAT
LVAN16507-qF	GCCACGGACAGCAACG
LVAN16507-qR	GCCAGGGTGTTGTTACTCTTC
MSTRG.252-qF	TTCTTCGGGTCTTGCTTG
MSTRG.252-qR	GAAAATCCCCCCCCAAAA
MSTRG.33445-qF	TCAAATCCGAAGATGAAGT
MSTRG.33445-qR	TTAGTTGTTAGACCCGTGT
LVAN10801-qF	CGGGAGATGACAGAACAGAT
LVAN10801-qR	TGGGGAGACGGTAGGTAG
LVAN24425-qF	GGCGAGTACGGAGATGGTGT
LVAN24425-qR	GAGTTCGTGTCGTGGGCAGA
LVAN14747-qF	TGGAGAGTGAGCAAGATGTT
LVAN14747-qR	ACTGGGTCCTCACTGCTTG
LVAN14777-qF	GCCTCCTGACACAATACAT
LVAN14777-qR	GCTTAGCTTCTGCTCCTAC
MSTRG.27945-qF	AAATCAACTAAACCTTTTCACC
MSTRG.27945-qR	AAACTTTTTCTCCTCTCTCTCA
LVAN09257-qF	TTGGTTTCACCTTCAGCTT
LVAN09257-qR	TCTTCCTTCCACTCCTTCG

### Metabolite extraction and detection

Metabolites were extracted according to the protocol described by Shanghai Applied Protein Technology Co, Ltd., Shanghai, China. Briefly, the hepatopancreas samples were cut on dry ice (~80 mg) into an Eppendorf tube (2 mL). The tissue samples with 200 µL of H_2_O and five ceramic beads were homogenized using the homogenizer. A total of 800 µL methanol/acetonitrile (1:1, vol/vol) were added to the homogenized solution for metabolite extraction. The mixture was centrifuged at 14,000 g, 4°C for 15 min. The supernatant was dried in a vacuum centrifuge. For LC-MS analysis, the samples were re-dissolved in 100 µL acetonitrile/water (1:1, vol/vol) solvent. To monitor the stability and repeatability of instrument analysis, quality control (QC) samples were prepared by pooling 10 μL of each sample and analyzed together with the other samples. The QC samples were inserted regularly and analyzed in every five samples.

Analysis was performed using an UHPLC (1290 Infinity LC, Agilent Technologies) coupled to a quadrupole time of flight (AB Sciex TripleTOF 6600). A 2.1 mm × 100 mm ACQUIY UPLC BEH 1.7 µM column (Waters, Ireland) was used for HILIC separation. The mobile phase (A: 25 mM ammonium acetate, 25 mM ammonium hydroxide; B: acetonitrile) used in both ESI positive and negative modes: 85% B for 1 min, then linearly reduced to 65% in 11 min, then reduced to 40% in 0.1 min and kept for 4 min, then increased to 85% in 0.1 min, with a 5 min re-equilibration. The ESI source conditions: Gas1 60, Gas2 60, CUR 30, source temperature: 600°C, Ion Spray Voltage Floating (ISVF) ± 5500 V. In MS only acquisition, *m*/z range 60–1000 Da, accumulation time for TOF MS scan 0.20 s/spectra. In auto MS/MS acquisition, *m*/z range 25–1000 Da, accumulation time 0.05 s/spectra. The ion scan is acquired using information dependent acquisition (IDA) with high sensitivity mode and parameters: the collision energy (CE) 35 V with ±15 eV; declustering potential (DP) 60 V (+) and −60 V (−); exclude isotopes within 4 Da, candidate ions to monitor per cycle: 10.

### Analysis of differential metabolites

The raw MS data were converted to MzXML files using ProteoWizard MSConvert before importing into freely available XCMS software. CAMERA (Collection of Algorithms of MEtabolite pRofile Annotation) was sued for annotation of isotopes and adducts. In the extracted ion features, only the variables having more than 50% of the nonzero measurement values in at least one group were kept. Compound identification of metabolites was performed by comparing of accuracy *m*/z value (<25 ppm), and MS/MS spectra with an in-house database established with available authentic standards.

After sum-normalization, the processed data were analyzed by R package (ropls), where it was subjected to multivariate data analysis, including PCA and OPLS-DA. The sevenfold cross-validation and response permutation testing were used to evaluate the robustness of the model. The variable importance in the projection (VIP) value of each variable in the OPLS-DA model was calculated to indicate its contribution to the classification. Student’s *t*-test was applied to determine the significance of differences between two groups of independent samples. VIP > 1 and *P* value < 0.05 were used to screen significant changed metabolites. Pearson’s correlation analysis was performed to determine the correlation between two variables.

### Integrative analysis of transcriptomes and metabolomes

KEGG pathway maps are the linking of genomic or transcriptomic contents of genes to chemical structures of endogenous molecules; thus providing a method to perform integration analysis of genes and metabolites. Pearson correlation coefficients and shared KEGG pathways were used to display the correlation between the transcriptomic and the metabolomic data. All differentially expressed genes and metabolites in this study were mapped to the KEGG pathway database to obtain their links in metabolic pathways.

### Statistical analysis

The relative transcription levels of different genes were obtained using 2^−ΔΔCt^ method (53). The numerical data from each experiment were analyzed to calculate the mean and standard deviation of triplicate assays. The significant differences among groups were subjected to one-way analysis of variance (ANOVA) and multiple comparisons using SPSS 19.0 program (54).

## Data Availability

The raw reads of the transcriptomic data are available at the Sequence Read Archive (SRA) under accession number SRR23117480-SRR23117491. The raw reads of the metabolomic data are available from the corresponding author upon request.

## AUTHOR NOTE

Author order was determined by the corresponding author after negotiation.

## SUPPLEMENTAL MATERIAL

Fig. S1 msystems.00067-23-s0001.tif Survival rate analysis of the resistant family (R20523) and susceptible family (S20507).

Fig. S2 msystems.00067-23-s0002.tif PCA analysis of all transcriptomic samples.

Fig. S3 msystems.00067-23-s0003.tif OPLS-DA models for analyzing the metabolomic data.

Fig. S4 msystems.00067-23-s0004.tif The shared KEGG pathways of DEGs and DMs. (A) The shared KEGG pathways of DEGs and DMs between resistant and susceptible families under unchallenged condition. (B) The shared KEGG pathways of DEGs and DMs between resistant and susceptible families after VpAHPND infection. (C) The shared KEGG pathways of DEGs and DMs responded to VpAHPND infection in susceptible family. (D) The shared KEGG pathways of DEGs and DMs responded to VpAHPND infection in resistant family.

TABLE S1 msystems.00067-23-s0005.pdf Statistics of the transcriptome sequencing data.

TABLE S2 msystems.00067-23-s0006.pdf Parameters of OPLS-DA model for analyzing the metabolome data.

TABLE S3 msystems.00067-23-s0007.pdf DEGs and DMs between resistant and susceptible families under unchallenged condition.

TABLE S4 msystems.00067-23-s0008.pdf DEGs and DMs between resistant and susceptible families after AHPND infection.

TABLE S5 msystems.00067-23-s0009.pdf DEGs and DMs responded to AHPND infection in susceptible family.

TABLE S6 msystems.00067-23-s0010.pdf DEGs and DMs responded to AHPND infection in resistant family.

ASM does not own the copyrights to Supplemental Material that may be linked to, or accessed through, an article. The authors have granted ASM a non-exclusive, world-wide license to publish the Supplemental Material files. Please contact the corresponding author directly for reuse.
