# Supplementary figures and images for: Integrated application of transcriptomics and metabolomics provides insights into acute hepatopancreatic necrosis disease resistance of Pacific white shrimp Litopenaeus vannamei
Source: mSystems. 2023 Jun 26;8(4):e00067-23. doi: 10.1128/msystems.00067-23 (PMC10469596; doi:10.1128/msystems.00067-23)

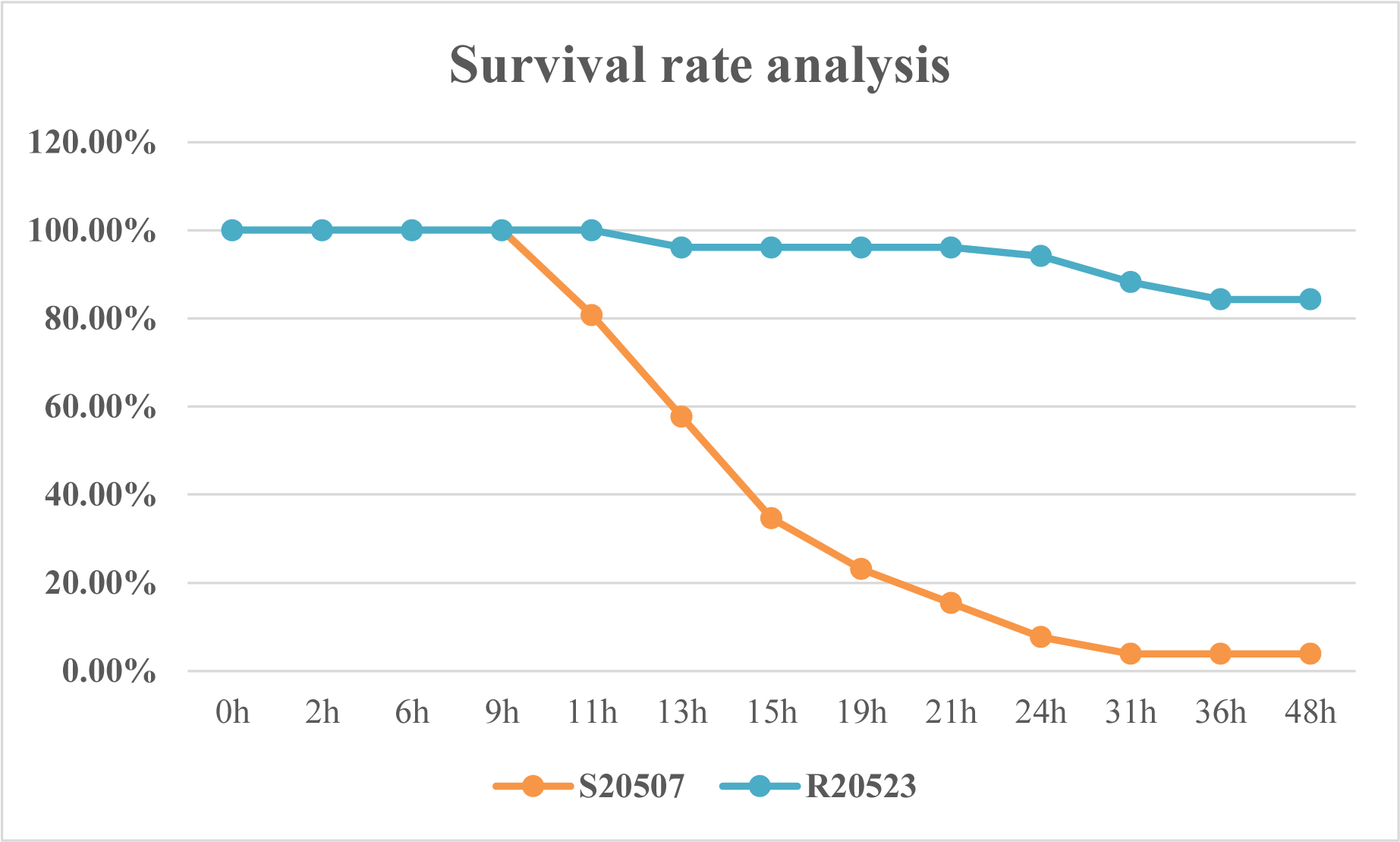

Supplement: Fig. S1 — Survival rate analysis of the resistant family (R20523) and susceptible family (S20507). [file msystems.00067-23-s0001.tif]

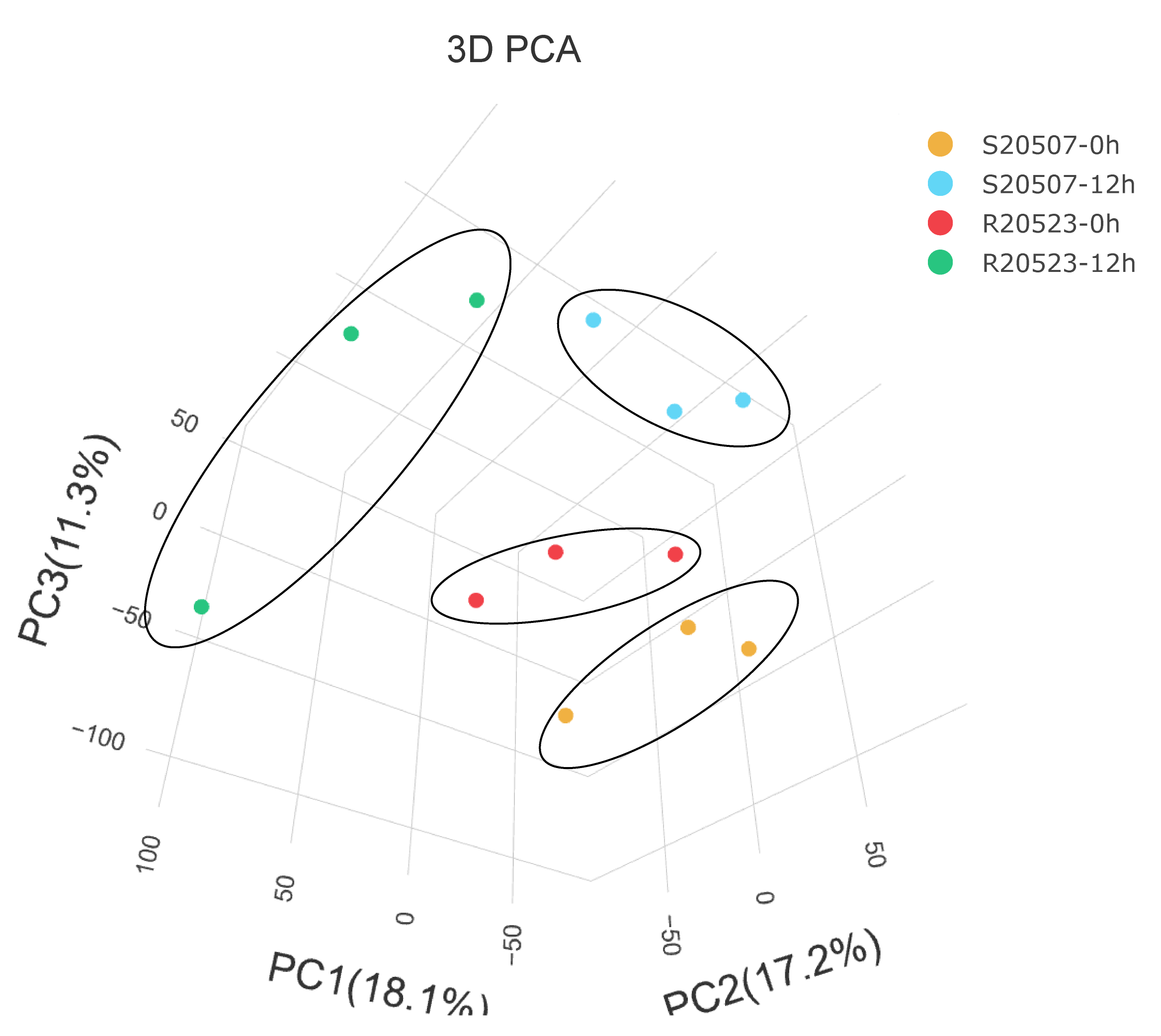

Supplement: Fig. S2 — PCA analysis of all transcriptomic samples. [file msystems.00067-23-s0002.tif]

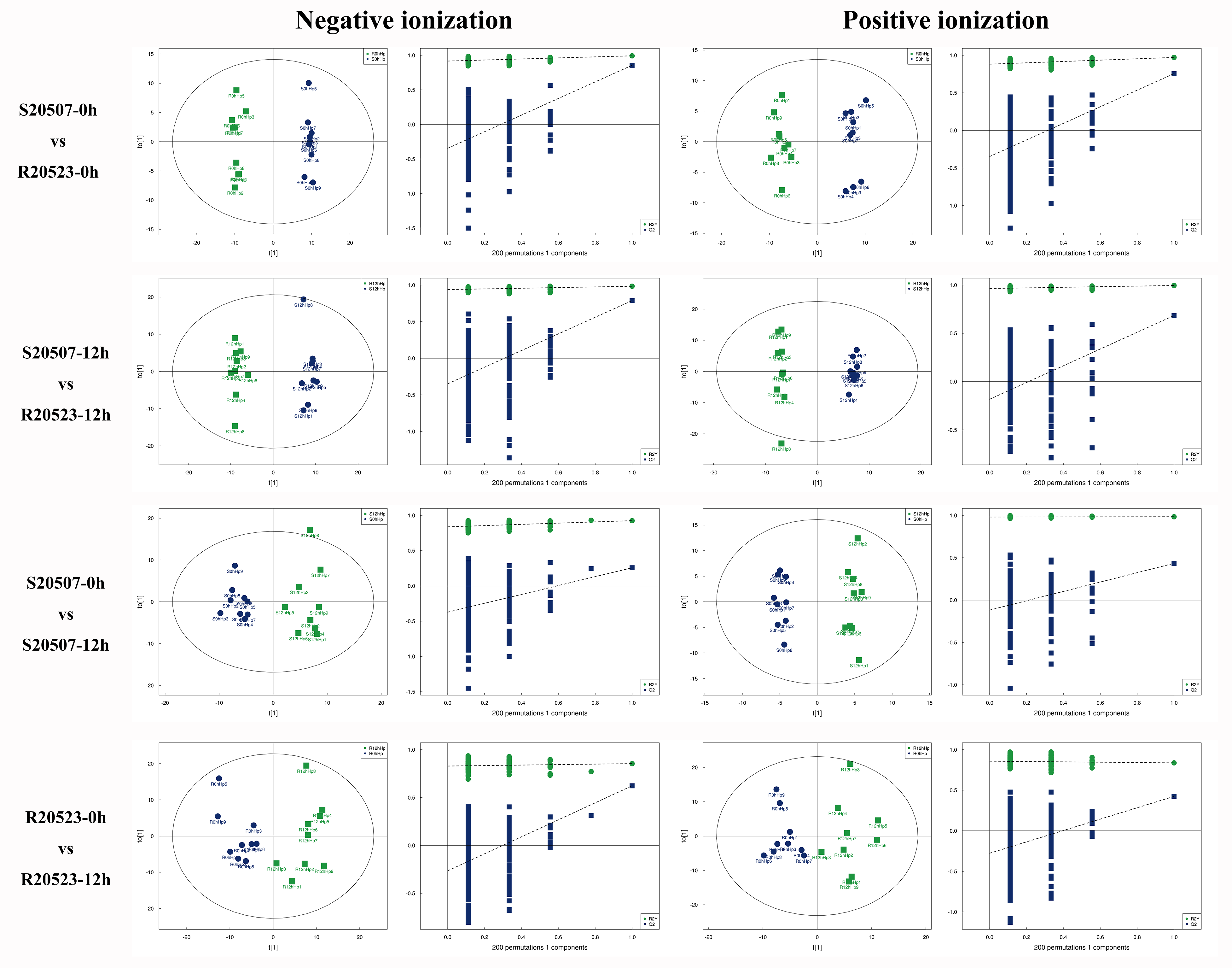

Supplement: Fig. S3 — OPLS-DA models for analyzing the metabolomic data. [file msystems.00067-23-s0003.tif]

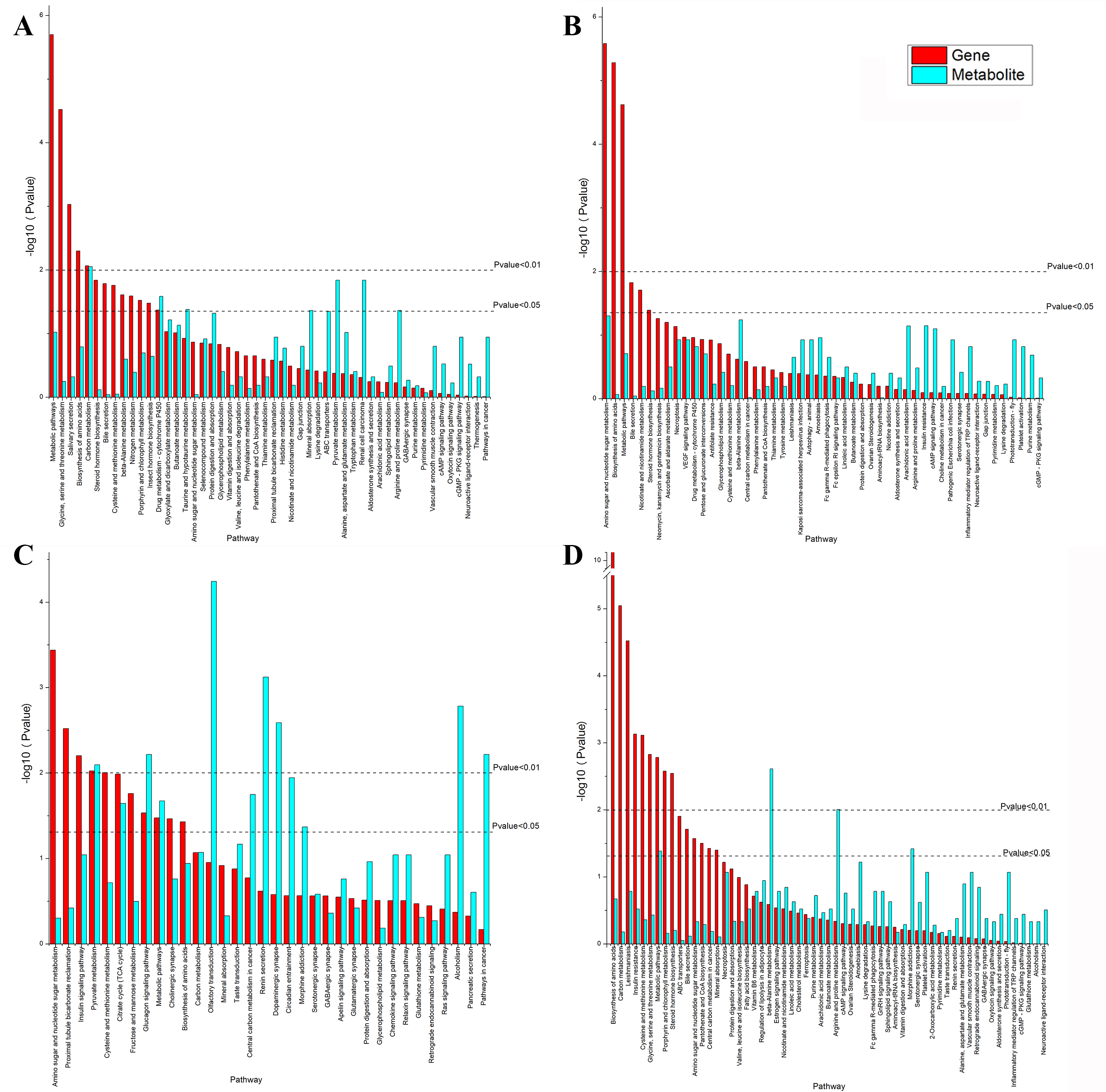

Supplement: Fig. S4 — The shared KEGG pathways of DEGs and DMs. (A) The shared KEGG pathways of DEGs and DMs between resistant and susceptible families under unchallenged condition. (B) The shared KEGG pathways of DEGs and DMs between resistant and susceptible families after VpAHPND infection. (C) The shared KEGG pathways of DEGs and DMs responded to VpAHPND infection in susceptible family. (D) The shared KEGG pathways of DEGs and DMs responded to VpAHPND infection in resistant family. [file msystems.00067-23-s0004.tif]
