# Supplementary material for: Integrated application of transcriptomics and metabolomics provides insights into acute hepatopancreatic necrosis disease resistance of Pacific white shrimp Litopenaeus vannamei
Source: mSystems. 2023 Jun 26;8(4):e00067-23. doi: 10.1128/msystems.00067-23 (PMC10469596; doi:10.1128/msystems.00067-23)
Supplement: TABLE S1 — Statistics of the transcriptome sequencing data. [file msystems.00067-23-s0005.pdf]

Table S1. Statistics of the transcriptome sequencing data

Group	Sample	Clean reads	Q30 (%)	GC content (%)	Mapped reads (%)
S20507-0h	1	43021886	92.87	44.73	83.99
	2	45222898	93.24	44.37	84.91
	3	41573108	93.23	45.13	83.95
S20507-12h	1	48981682	93.23	45.65	84.16
	2	56799356	93.09	46.42	84.88
	3	52044276	93.22	45.60	85.06
R20523-0h	1	55932948	93.33	44.77	83.41
	2	50272288	93.69	44.11	81.96
	3	52602678	93.08	44.78	84.23
R20523-12h	1	56151306	93.06	44.86	84.41
	2	53287068	93.13	43.75	83.91
	3	48544792	93.16	45.53	84.78
