# Supplementary material for: Integrated application of transcriptomics and metabolomics provides insights into acute hepatopancreatic necrosis disease resistance of Pacific white shrimp Litopenaeus vannamei
Source: mSystems. 2023 Jun 26;8(4):e00067-23. doi: 10.1128/msystems.00067-23 (PMC10469596; doi:10.1128/msystems.00067-23)
Supplement: TABLE S2 — Parameters of OPLS-DA model for analyzing the metabolome data. [file msystems.00067-23-s0006.pdf]

Table S2. Parameters of OPLS-DA model for analyzing the metabolome data

	Negative ionization			Positive ionization		
	R2X(cum)	R2Y(cum)	Q2(cum)	R2X(cum)	R2Y(cum)	Q2(cum)
R20523-0h_vs_S20507-0h	0.404	0.992	0.855	0.29	0.971	0.756
R20523-12h_vs_S20507-12h	0.457	0.983	0.787	0.428	0.995	0.684
S20507-12h_vs_S20507-0h	0.525	0.995	0.694	0.335	0.984	0.434
R20523-12h_vs_R20523-0h	0.393	0.854	0.622	0.402	0.837	0.424
