# Supplementary material for: Integrated application of transcriptomics and metabolomics provides insights into acute hepatopancreatic necrosis disease resistance of Pacific white shrimp Litopenaeus vannamei
Source: mSystems. 2023 Jun 26;8(4):e00067-23. doi: 10.1128/msystems.00067-23 (PMC10469596; doi:10.1128/msystems.00067-23)
Supplement: TABLE S3 — DEGs and DMs between resistant and susceptible families under unchallenged condition. [file msystems.00067-23-s0007.pdf]

Table S3. DEGs and DMs between resistant and susceptible families under unchallenged condition

Gene_ID	S20507-0h	R20523-0h	log2(fc)	FDR	Description
MSTRG.11151	42.37333	0.001	-15.3709	3.99E-26	phosphoglucomutase [Penaeus vannamei]
MSTRG.25653	8.336667	0.001	-13.0253	1.38E-05	-
MSTRG.40876	6.1	0.001	-12.5746	7.15E-07	endocuticle structural glycoprotein SgAbd-1-like [Penaeus vannamei]
MSTRG.21289	3.8	0.001	-11.8918	0.040717	-
MSTRG.13337	2	0.001	-10.9658	0.015362	chymotrypsin BI-like [Penaeus vannamei]
MSTRG.30331	1.896667	0.001	-10.8893	9.62E-10	-
MSTRG.33516	1.873333	0.001	-10.8714	5.79E-07	-
MSTRG.15688	1.23	0.001	-10.2644	0.016956	cuticle protein AMP13.4 [Penaeus vannamei]
LVAN16587	0.983333	0.001	-9.94154	0.000398	Oxysterol-binding protein-related protein 10 [Columba livia]
MSTRG.33461	0.853333	0.001	-9.73697	0.04709	LOW QUALITY PROTEIN: protein piccolo [Camelus ferus]
MSTRG.18769	0.786667	0.001	-9.61961	0.011157	pro-resilin-like [Penaeus vannamei]
LVAN01901	0.313333	0.001	-8.29155	0.000597	Chitinase-3-like protein 3 [Zootermopsis nevadensis]
LVAN04686	0.29	0.001	-8.17991	0.045603	Cuticlin-1 [Bactrocera dorsalis]
MSTRG.40878	3.666667	0.023333	-7.29593	2.42E-05	endocuticle structural glycoprotein SgAbd-2-like [Penaeus vannamei]
LVAN09935	25.49	0.226667	-6.81322	3.01E-08	acid phosphatase [Litopenaeus vannamei]
MSTRG.40883	4.003333	0.043333	-6.52958	0.000381	cuticle protein AMP1A-like isoform X1 [Penaeus vannamei]
MSTRG.37237	1.846667	0.02	-6.52878	2.73E-19	hypothetical protein AS27_10936, partial [Aptenodytes forsteri]
MSTRG.31336	2.186667	0.03	-6.18763	0.001073	neurotrophin 1-like [Penaeus vannamei]
MSTRG.1915	2.236667	0.04	-5.80521	0.008378	-
MSTRG.29883	2.226667	0.046667	-5.57635	0.001686	prolow-density lipoprotein receptor-related protein 1-like [Penaeus vannamei]
LVAN18582	0.883333	0.02	-5.46489	0.029283	PREDICTED: proton-coupled folate transporter-like [Cimex lectularius]
MSTRG.33343	4.733333	0.113333	-5.38421	0.004001	cuticle protein 18.6-like [Penaeus vannamei]
MSTRG.25782	2.096667	0.053333	-5.29692	0.017964	uncharacterized protein LOC113818759 [Penaeus vannamei]

MSTRG.41094	4.593333	0.12	-5.25844	2.07E-05	-
MSTRG.40879	1.723333	0.046667	-5.20667	0.034738	cuticle protein CP14.6-like [Penaeus vannamei]
MSTRG.39625	0.49	0.013333	-5.19967	0.047712	Chitin binding domain [Trinorchestia longiramus]
MSTRG.36351	0.616667	0.02	-4.94642	0.048594	cubilin-like [Penaeus vannamei]
MSTRG.7585	11.11	0.386667	-4.84462	1.93E-05	-
MSTRG.14451	0.756667	0.026667	-4.82655	0.037366	leucine-rich repeat extensin-like protein 3 [Penaeus vannamei]
LVAN21074	3.693333	0.14	-4.72142	2.42E-05	-
LVAN20363	1.17	0.046667	-4.64797	0.020403	-
LVAN21073	7.416667	0.3	-4.62774	4.87E-08	PREDICTED: agglutinin-like protein ARB_02240 isoform X2 [Hyalella azteca]
MSTRG.24253	10.97	0.47	-4.54476	1.82E-09	Transposon Ty3-G Gag-Pol polyprotein [Stylophora pistillata]
MSTRG.31773	8.693333	0.373333	-4.54137	1.06E-06	dnaJ homolog subfamily C member 9-like [Penaeus vannamei]
LVAN03083	8.53	0.38	-4.48847	0.000235	-
MSTRG.28687	0.646667	0.03	-4.42999	0.034887	coagulation factor XI-like [Penaeus vannamei]
LVAN04601	0.816667	0.04	-4.35168	0.045058	JHE-like carboxylesterase 1 [Pandalopsis japonica]
MSTRG.36350	0.796667	0.04	-4.3159	0.046568	cubilin-like [Penaeus vannamei]
MSTRG.4033	0.496667	0.026667	-4.21917	0.019798	GP63, leishmanolysin [Leishmania tarentolae]
MSTRG.40881	6.44	0.406667	-3.98514	0.000324	cuticle protein AM/CP1114-like [Penaeus vannamei]
LVAN06157	5.976667	0.393333	-3.92552	1.73E-08	clip domain serine protease [Eriocheir sinensis]
MSTRG.5322	2.123333	0.14	-3.92283	0.002005	hypothetical protein C7M84_014460 [Penaeus vannamei]
MSTRG.24254	3.89	0.256667	-3.9218	8.31E-16	zinc finger protein OZF-like [Penaeus vannamei]
MSTRG.38	3.69	0.25	-3.88362	0.000559	uncharacterized protein LOC113820024 [Penaeus vannamei]
LVAN11401	0.583333	0.04	-3.86625	0.012722	PREDICTED: serine/arginine repetitive matrix protein 2 isoform X1 [Bemisia tabaci]
MSTRG.25394	1.733333	0.12	-3.85244	0.03509	hypothetical protein C7M84_015229 [Penaeus vannamei]
MSTRG.28800	6.39	0.453333	-3.81717	8.51E-10	prolow-density lipoprotein receptor-related protein 1-like [Penaeus vannamei]
LVAN02957	2.96	0.213333	-3.79442	9.09E-06	PREDICTED: glycine receptor subunit alphaZ1-like [Parasteatoda tepidariorum]
MSTRG.36517	4.43	0.33	-3.74677	3.84E-07	hypothetical protein FHG87_012622 [Trinorchestia longiramus]

LVAN19227	1.356667	0.103333	-3.71469	3.50E-07	hypothetical protein g.8709 [Clastoptera arizonana]
MSTRG.29950	4.81	0.366667	-3.7135	1.38E-05	monocarboxylate transporter 8-like [Penaeus vannamei]
LVAN13142	57.26	4.57	-3.64726	0.031229	PREDICTED: pancreatic lipase-related protein 2-like isoform X3 [Aethina tumida]
LVAN12499	0.456667	0.036667	-3.6386	0.043894	-
MSTRG.33067	1.433333	0.116667	-3.61891	0.00765	uncharacterized protein LOC113824167 [Penaeus vannamei]
LVAN23464	1.293333	0.106667	-3.59991	0.012722	PREDICTED: MORC family CW-type zinc finger protein 3-like isoform X1 [Salmo salar]
MSTRG.28661	3.606667	0.333333	-3.43563	0.000315	uncharacterized protein LOC113820914 [Penaeus vannamei]
LVAN09590	3.413333	0.326667	-3.38529	2.51E-07	PREDICTED: protein SpAN-like isoform X2 [Hyalella azteca]
MSTRG.41646	3.876667	0.376667	-3.36346	0.005624	-
LVAN04947	5.916667	0.59	-3.326	1.65E-08	Chitooligosaccharidolytic beta-N-acetylglucosaminidase [Orchesella cincta]
LVAN04946	4.606667	0.463333	-3.3136	1.76E-07	Chitooligosaccharidolytic beta-N-acetylglucosaminidase [Orchesella cincta]
LVAN19111	1.536667	0.156667	-3.29403	0.000814	prophenoloxidase b [Marsupenaeus japonicus]
LVAN21698	40.05667	4.12	-3.28133	4.32E-14	PREDICTED: flavin-containing monooxygenase FMO GS-OX4-like [Lingula anatina]
LVAN07013	1.39	0.143333	-3.27764	0.006977	chitin deacetylase 1 [Penaeus monodon]
LVAN02078	1.48	0.153333	-3.27085	0.001478	-
LVAN20722	1.673333	0.176667	-3.24362	0.003554	PREDICTED: low-density lipoprotein receptor-related protein 2-like, partial [Parasteatoda tepidariorum]
LVAN01889	1.263333	0.136667	-3.2085	0.018646	-
MSTRG.2913	11.92333	1.343333	-3.1499	3.51E-11	uncharacterized protein LOC113808014 [Penaeus vannamei]
LVAN05074	43.34667	4.96	-3.12751	5.03E-13	cysteine sulfinic acid decarboxylase [Litopenaeus vannamei]
LVAN20370	6.373333	0.763333	-3.06166	1.32E-15	-
LVAN20738	0.716667	0.093333	-2.94084	2.77E-08	PREDICTED: fibrocystin-L [Strongylocentrotus purpuratus]
LVAN23678	1.896667	0.256667	-2.8855	0.035918	PREDICTED: facilitated trehalose transporter Tret1-2 homolog [Hyalella azteca]
LVAN18451	4.686667	0.646667	-2.85747	0.000488	glycosyl-phosphatidylinositol-linked carbonic anhydrase [Carcinus maenas]
LVAN05507	4.39	0.606667	-2.85524	2.44E-05	-
MSTRG.19941	2.313333	0.323333	-2.83888	0.000942	phosphopantothenoylcysteine decarboxylase subunit VHS3-like [Penaeus vannamei]

LVAN18616	1.016667	0.143333	-2.8264	0.046993	Variant Ionotropic Glutamate Receptor [Coenobita clypeatus]
LVAN21716	1.556667	0.22	-2.82288	0.016817	PREDICTED: monocarboxylate transporter 12 [Nicrophorus vespilloides]
LVAN16507	4.59	0.673333	-2.7691	2.15E-09	PREDICTED: platelet binding protein GspB-like [Xenopus tropicalis]
LVAN11204	8.386667	1.243333	-2.75388	7.62E-05	PREDICTED: b(0,+)-type amino acid transporter 1-like, partial [Hyalella azteca]
MSTRG.26606	2.646667	0.393333	-2.75035	0.006616	mucin-2-like [Penaeus vannamei]
LVAN04920	1.803333	0.27	-2.73963	0.000332	protease [Homarus americanus]
MSTRG.18203	0.96	0.146667	-2.71049	0.00016	-
LVAN19167	1.836667	0.286667	-2.67964	0.013527	gastrolith protein [Cherax quadricarinatus]
MSTRG.11139	5.363333	0.856667	-2.64632	0.015083	single VWC domain protein 1 [Penaeus vannamei]
LVAN24425	42.74667	6.973333	-2.61589	5.19E-12	PREDICTED: hemocyte protein-glutamine gamma-glutamyltransferase-like [Hyalella azteca]
MSTRG.35922	30.71667	5.03	-2.61039	2.18E-07	calcium-activated chloride channel regulator 4A-like isoform X2 [Penaeus vannamei]
MSTRG.35624	2.176667	0.356667	-2.60947	0.043894	venom protein 59.1-like [Penaeus vannamei]
LVAN02631	2.713333	0.446667	-2.6028	0.031537	PREDICTED: mitochondrial basic amino acids transporter-like isoform X1 [Aedes albopictus]
LVAN09956	7.39	1.236667	-2.57912	7.99E-08	Glucosylceramidase precursor [Daphnia magna]
MSTRG.31411	14.54	2.496667	-2.54195	1.35E-19	hypothetical protein C7M84_014460 [Penaeus vannamei]
MSTRG.8295	3.966667	0.69	-2.52326	0.000792	-
LVAN12468	3.75	0.663333	-2.49908	0.000247	juvenile hormone esterase-like carboxylesterase 1 [Eriocheir sinensis]
MSTRG.23575	6.37	1.18	-2.43251	0.02806	integrin alpha 8 [Penaeus chinensis]
LVAN06266	0.55	0.103333	-2.41213	0.000153	PREDICTED: sushi, von Willebrand factor type A, EGF and pentraxin domain-containing protein 1 [Ceratina calcarata]
LVAN18695	7.96	1.613333	-2.30272	0.001209	-
MSTRG.2622	7.283333	1.53	-2.25107	0.006423	cylicin-1-like [Penaeus vannamei]
LVAN10801	8.573333	1.823333	-2.23328	1.11E-07	trehalose-6-phosphate synthase [Fenneropenaeus chinensis]
MSTRG.10008	5.306667	1.156667	-2.19783	0.030945	-

LVAN16084	8.406667	1.856667	-2.17882	0.006027	antimicrobial peptide type 1 precursor Ic [Pandalopsis japonica]
LVAN14747	69.34667	15.83	-2.13117	8.08E-07	PREDICTED: methylenetetrahydrofolate reductase-like [Hyalella azteca]
MSTRG.802	6.25	1.443333	-2.11445	0.001408	neuropeptide-like protein 31 [Penaeus vannamei]
MSTRG.39649	238.9167	55.69333	-2.10093	1.11E-05	uncharacterized protein LOC113829253 [Penaeus vannamei]
MSTRG.25024	7.923333	1.85	-2.09858	0.004894	-
MSTRG.25077	20.88	4.886667	-2.0952	0.000955	-
MSTRG.10451	23.55333	5.526667	-2.09145	0.00038	-
MSTRG.23922	2.043333	0.49	-2.06007	0.009244	-
MSTRG.680	5.733333	1.4	-2.03395	0.002685	plectin-like [Penaeus vannamei]
LVAN03486	28.02667	6.883333	-2.02562	2.73E-05	PREDICTED: D-3-phosphoglycerate dehydrogenase-like [Hyalella azteca]
LVAN05801	0.866667	0.213333	-2.02237	0.00909	PREDICTED: probable chitinase 3 isoform X1 [Hyalella azteca]
LVAN03028	73.51333	18.21667	-2.01275	1.90E-05	peritrophic membrane chitin binding protein [Culex quinquefasciatus]
LVAN19903	9.616667	2.393333	-2.00652	1.38E-05	PREDICTED: uncharacterized protein LOC108668843, partial [Hyalella azteca]
LVAN15665	3.643333	0.913333	-1.99605	0.008635	PREDICTED: tubulin alpha-3 chain-like [Hyalella azteca]
MSTRG.36985	16.36	4.15	-1.97899	3.79E-05	PREDICTED: uncharacterized oxidoreductase C26H5.09c-like [Linepithema humile]
LVAN08495	9.886667	2.57	-1.94372	0.037801	PREDICTED: biglycan [Poecilia reticulata]
LVAN00802	3.533333	0.92	-1.94132	0.004001	cytochrome P450 [Panulirus argus]
LVAN16504	25.79333	6.8	-1.92339	0.000216	PREDICTED: receptor-type tyrosine-protein phosphatase alpha-like [Hyalella azteca]
LVAN08223	18.38667	4.89	-1.91075	1.55E-06	Brix domain-containing protein 1 [Zootermopsis nevadensis]
LVAN18894	16.41	4.416667	-1.89355	6.17E-05	PREDICTED: carboxypeptidase B-like [Hyalella azteca]
MSTRG.43249	15.44	4.156667	-1.89317	0.000522	-
LVAN01890	4.53	1.23	-1.88085	0.000135	200 kDa antigen p200, partial [Babesia bigemina]
MSTRG.23218	5.996667	1.646667	-1.86461	0.016611	hypothetical protein C7M84_008994 [Penaeus vannamei]
MSTRG.13636	8.896667	2.46	-1.85461	3.16E-07	-
MSTRG.32755	8.99	2.5	-1.84639	0.000952	hypothetical protein XELAEV_18003705mg [Xenopus laevis]
MSTRG.14726	21.09667	5.883333	-1.84231	2.18E-05	-

MSTRG.22397	27.12	7.646667	-1.82645	9.71E-05	-
LVAN00362	5.3	1.5	-1.82103	0.000789	juvenile hormone esterase-like carboxylesterase 1 [Eriocheir sinensis]
LVAN20408	3.403333	0.963333	-1.82084	4.84E-05	hemolymph clottable protein [Litopenaeus vannamei]
MSTRG.33778	12.21	3.463333	-1.81783	0.003053	zf-CCHC and Asp protease 2 and rve domain containing ing protein [Penaeus vannamei]
MSTRG.11174	19.44333	5.566667	-1.80439	3.23E-05	lethal(2) giant larvae protein homolog 2 isoform X1 [Gadus morhua]
LVAN00201	11.26	3.243333	-1.79566	0.007303	PDGF/VEGF-related factor 1 [Eriocheir sinensis]
MSTRG.31743	6.033333	1.74	-1.79387	0.000531	neuropilin-1-like isoform X1 [Penaeus vannamei]
LVAN00911	5.74	1.67	-1.7812	0.000463	Nose resistant to fluoxetine protein 6, partial [Stegodyphus mimosarum]
LVAN14202	37.29667	10.87667	-1.77781	8.14E-11	Zinc/iron regulated transporter-related protein 42C.2 [Daphnia magna]
MSTRG.22337	1.83	0.536667	-1.76975	0.000531	-
MSTRG.19804	3.586667	1.06	-1.75858	0.009504	hypothetical protein C7M84_025354 [Penaeus vannamei]
LVAN09584	2519.897	744.77	-1.7585	5.29E-11	C-type lectin [Litopenaeus vannamei]
LVAN08158	149.95	44.41667	-1.75531	0.000455	amylase I, partial [Litopenaeus vannamei]
MSTRG.27945	2.953333	0.876667	-1.75224	0.001478	Monocarboxylate transporter 12, partial [Armadillidium vulgare]
MSTRG.252	3.926667	1.17	-1.7468	0.04493	-
LVAN12822	10.34	3.09	-1.74256	0.002116	PREDICTED: UDP-glucuronosyltransferase-like isoform X4 [Hyalella azteca]
LVAN12472	4.28	1.286667	-1.73397	0.046906	juvenile hormone esterase-like carboxylesterase 1 [Eriocheir sinensis]
MSTRG.34128	5.346667	1.616667	-1.72562	0.006884	sterol-regulatory element binding protein [Penaeus vannamei]
LVAN00179	12.95	4.03	-1.6841	0.00012	PREDICTED: FK506-binding protein 4-like [Megachile rotundata]
LVAN01162	5.696667	1.776667	-1.68094	0.027304	PREDICTED: platelet glycoprotein Ib alpha chain [Aotus nancymaae]
MSTRG.35455	12.55	3.933333	-1.67386	0.000399	-
LVAN12304	7.993333	2.56	-1.64265	0.013649	-
LVAN20946	28.49333	9.126667	-1.64246	0.004519	PREDICTED: alkylglycerol monooxygenase-like [Hyalella azteca]
MSTRG.14731	131.2567	42.29	-1.634	3.28E-05	uncharacterized protein LOC113810182 [Penaeus vannamei]
LVAN16399	3.97	1.33	-1.57771	0.029407	PREDICTED: uncharacterized protein LOC106470123 [Limulus polyphemus]
LVAN06120	22.38	7.513333	-1.57469	0.007541	juvenile hormone esterase-like carboxylesterase 1 [Eriocheir sinensis]

MSTRG.35540	14.99667	5.056667	-1.56838	0.021312	titin-like [Penaeus vannamei]
LVAN13033	76.8	25.92333	-1.56686	0.003872	PREDICTED: beta,beta-carotene 15,15'-dioxygenase-like isoform X1 [Hyalella azteca]
LVAN05673	27.92667	9.456667	-1.56224	8.11E-05	PREDICTED: phosphatidylserine decarboxylase proenzyme, mitochondrial-like isoform X1 [Hyalella azteca]
LVAN16093	89.86333	30.98667	-1.53609	0.031173	chitin binding-like protein [Fenneropenaeus chinensis]
LVAN10749	13.53333	4.696667	-1.52681	0.013299	PREDICTED: cold shock-induced protein TIR1 [Drosophila ficusphila]
MSTRG.37624	22.59667	7.906667	-1.51497	6.96E-05	-
LVAN14777	74.21333	25.98333	-1.51409	4.91E-05	PREDICTED: methionine synthase [Apteryx australis mantelli]
LVAN24489	3.553333	1.25	-1.50724	0.040394	caspase 4 [Litopenaeus vannamei]
MSTRG.25076	23.03667	8.163333	-1.4967	0.001789	-
LVAN12098	47.98	17.00667	-1.49633	0.003314	alkaline phosphatase [Litopenaeus vannamei]
LVAN02470	5.996667	2.13	-1.49331	0.003829	ankyrin 2,3/unc44, partial [Riptortus pedestris]
LVAN14971	9.23	3.296667	-1.48532	0.009376	PREDICTED: diablo homolog, mitochondrial [Kryptolebias marmoratus]
LVAN08887	23.29	8.326667	-1.4839	0.004636	alpha-amylase [Marsupenaeus japonicus]
LVAN20143	15.26	5.53	-1.4644	3.73E-08	PREDICTED: NLR family CARD domain-containing protein 4-like [Hyalella azteca]
MSTRG.3093	21.03	7.723333	-1.44515	0.01594	-
MSTRG.28647	19.35333	7.116667	-1.44331	0.018015	group 3 secretory phospholipase A2-like [Penaeus vannamei]
MSTRG.41898	222.8	81.95333	-1.44287	4.91E-05	keratin-associated protein 16-1-like [Penaeus vannamei]
MSTRG.34214	22.71333	8.44	-1.42822	2.15E-09	neurotrophin 1-like [Penaeus vannamei]
MSTRG.9300	23.97333	8.993333	-1.4145	1.97E-09	-
LVAN17723	13.96	5.333333	-1.38819	0.03995	PREDICTED: regulator of G-protein signaling 2-like [Hyalella azteca]
LVAN18801	9.12	3.513333	-1.37619	0.034864	PREDICTED: SE-cephalotoxin-like [Callorhinchus milii]
MSTRG.31988	16.45333	6.443333	-1.3525	0.005823	prolow-density lipoprotein receptor-related protein 1-like [Penaeus vannamei]
MSTRG.21911	2.483333	0.973333	-1.35127	0.039317	-
LVAN05702	16.92667	6.646667	-1.34859	4.28E-06	juvenile hormone esterase-like carboxylesterase 1 [Eriocheir sinensis]
LVAN25149	8.87	3.513333	-1.33609	0.000315	apoptosis-inducing factor [Litopenaeus vannamei]

MSTRG.36295	112.6867	44.77	-1.33171	0.014839	endo-beta-1,4-glucanase [Penaeus vannamei]
MSTRG.33708	66.24333	26.42333	-1.32596	3.26E-08	poly [ADP-ribose] polymerase 12-like [Penaeus vannamei]
MSTRG.41674	254.1933	102.2333	-1.31406	0.000503	neurogenic locus notch homolog protein 1-like isoform X4 [Penaeus vannamei]
LVAN04619	93.38333	37.66667	-1.30988	0.010596	juvenile hormone esterase-like carboxylesterase 1 [Eriocheir sinensis]
LVAN09943	70.72667	28.72667	-1.29986	2.18E-07	PREDICTED: NACHT, LRR and PYD domains-containing protein 3-like [Strongylocentrotus purpuratus]
LVAN18110	37.79333	15.36667	-1.29833	0.01594	PREDICTED: phosphoserine phosphatase [Cimex lectularius]
LVAN19124	10.81333	4.403333	-1.29614	0.021662	PREDICTED: arylsulfatase B-like isoform X2 [Branchiostoma belcheri]
LVAN05432	29.27667	11.94667	-1.29314	0.001209	Lactosylceramide [Daphnia magna]
LVAN12789	90.73	37.17	-1.28744	0.008263	endo-beta-1,4-glucanase, partial [Coenobita brevimanus]
MSTRG.24093	4.153333	1.706667	-1.28309	0.007492	AT-rich interactive domain-containing protein 1A-like [Petromyzon marinus]
LVAN00797	11.31333	4.726667	-1.25913	0.015054	Sodium-coupled neutral amino acid transporter [Daphnia magna]
MSTRG.2219	20.02333	8.396667	-1.25379	0.000732	SICAvar Type I [Plasmodium knowlesi]
LVAN16233	13.46667	5.766667	-1.22358	0.004277	PREDICTED: glycine N-acyltransferase-like protein 3 isoform X1 [Ictalurus punctatus]
LVAN11905	29.01667	12.46667	-1.21881	0.013669	PREDICTED: 2-hydroxyacylsphingosine 1-beta-galactosyltransferase-like [Hyalella azteca]
LVAN11152	13.74	5.92	-1.21471	0.013669	PREDICTED: quinone oxidoreductase isoform X1 [Xenopus tropicalis]
LVAN15732	55.01	23.76	-1.21116	0.006554	EF-hand domain-containing protein D1 [Crassostrea gigas]
MSTRG.19801	12.71333	5.5	-1.20884	0.006393	FR47-like [Trinorchestia longiramus]
LVAN21222	51.61667	22.34667	-1.20778	0.00037	PREDICTED: cytochrome P450 9e2-like [Hyalella azteca]
LVAN00042	49.13333	21.38	-1.20044	0.018729	PREDICTED: UPF0462 protein C4orf33 homolog isoform X1 [Anolis carolinensis]
MSTRG.17954	6.666667	2.923333	-1.18935	0.00403	deleted in autism protein 1 homolog [Penaeus vannamei]
LVAN21223	106.5167	46.72	-1.18897	0.000536	PREDICTED: cytochrome P450 9e2-like [Hyalella azteca]
LVAN17855	18.73	8.28	-1.17765	0.000264	PREDICTED: xanthine dehydrogenase 1-like [Hyalella azteca]
LVAN03996	37.84333	16.83333	-1.16872	0.000147	PREDICTED: 26S proteasome non-ATPase regulatory subunit 10-like [Amphimedon queenslandica]
MSTRG.24861	10.47333	4.723333	-1.14884	0.036605	-

LVAN12007	33.10333	15.03333	-1.13881	0.039348	PREDICTED: vitelline membrane outer layer protein 1 homolog [Hyalella azteca]
MSTRG.38197	5.843333	2.656667	-1.13717	0.002948	protein sprouty-like, partial [Penaeus vannamei]
LVAN22142	57.56667	26.54333	-1.11688	0.003262	PREDICTED: cytochrome P450 9e2-like [Hyalella azteca]
LVAN11904	30.90333	14.26667	-1.11511	0.007485	PREDICTED: 2-hydroxyacylsphingosine 1-beta-galactosyltransferase-like [Hyalella azteca]
LVAN17856	15.34	7.083333	-1.1148	0.005624	PREDICTED: xanthine dehydrogenase 2-like [Hyalella azteca]
LVAN20647	12.14333	5.63	-1.10896	0.033408	PREDICTED: src kinase-associated phosphoprotein 1-like [Hyalella azteca]
LVAN24007	45.81667	21.37	-1.10029	0.017964	Abhydrolase domain-containing protein 3, partial [Zootermopsis nevadensis]
LVAN09517	143.6133	67.10667	-1.09766	0.000763	PREDICTED: probable cytochrome P450 49a1 [Hyalella azteca]
MSTRG.31046	3.113333	1.46	-1.09249	0.009207	serine/threonine-protein kinase fray2-like, partial [Cyclospora cayetanensis]
MSTRG.3094	128.1733	60.27	-1.08858	0.014677	-
LVAN00225	97.9	46.08333	-1.08706	0.001574	oxygenase [Oplophorus gracilirostris]
LVAN10270	100.5267	47.35667	-1.08594	0.007976	PREDICTED: von Willebrand factor A domain-containing protein 7-like isoform X1 [Branchiostoma belcheri]
LVAN12128	110.6567	52.93333	-1.06384	0.009624	oxygenase [Oplophorus gracilirostris]
MSTRG.24859	6.953333	3.336667	-1.0593	0.01594	-
MSTRG.33563	18.97333	9.166667	-1.0495	0.012399	-
MSTRG.29408	26.29667	12.70667	-1.04929	0.000435	Protein snakeskin [Amphibalanus amphitrite]
MSTRG.32674	23.86333	11.59	-1.04192	0.00154	-
MSTRG.13734	24.89667	12.09667	-1.04134	0.014677	coiled-coil domain-containing protein 167-like [Penaeus vannamei]
LVAN13328	251.87	122.5633	-1.03915	0.007773	PREDICTED: S-formylglutathione hydrolase [Crassostrea gigas]
LVAN00430	62.93667	30.76333	-1.03269	0.000167	PREDICTED: nose resistant to fluoxetine protein 6 [Drosophila biarmipes]
LVAN00688	1600.043	782.2733	-1.03237	0.001835	preamylase 1 [Litopenaeus vannamei]
LVAN22814	43.87	21.50667	-1.02845	0.014677	Putative proline-rich protein 21 [Myotis davidii]
MSTRG.3622	10.66333	5.23	-1.02778	0.018178	uncharacterized protein LOC113826500 [Penaeus vannamei]
LVAN24230	112.1267	55.16333	-1.02335	0.012722	amylase [Litopenaeus vannamei]
LVAN09820	101.39	49.94333	-1.02155	0.019041	PREDICTED: carboxypeptidase A1-like [Scleropages formosus]

LVAN20131	90.51333	44.64	-1.01979	0.009472	PREDICTED: serine hydroxymethyltransferase, cytosolic-like [Hyalella azteca]
MSTRG.39652	417.7333	206.85	-1.014	0.017552	uncharacterized protein LOC113829246 [Penaeus vannamei]
LVAN03638	26.67	13.28667	-1.00524	0.016956	PREDICTED: ATP-binding cassette sub-family A member 1-like [Hyalella azteca]
MSTRG.14536	561.7633	280.56	-1.00165	0.015654	peritrophin-44-like protein [Penaeus vannamei]
LVAN02911	34.92667	70.45667	1.012407	0.000531	C-type lectin-like protein [Fenneropenaeus chinensis]
MSTRG.25558	27.34	56.20667	1.039728	7.20E-07	-
MSTRG.15724	20.73667	42.88	1.048121	0.041869	uncharacterized protein LOC113824138 [Penaeus vannamei]
LVAN10950	621.2633	1286.01	1.049625	0.00036	PREDICTED: oplophorus-luciferin 2-monooxygenase non-catalytic subunit-like [Hyalella azteca]
LVAN03569	10.74333	22.56	1.070325	4.28E-06	PREDICTED: E3 ubiquitin-protein ligase TTC3 [Larimichthys crocea]
LVAN22769	345.78	732.98	1.083919	0.002008	hemocyanin subunit L2, partial [Litopenaeus vannamei]
LVAN22253	1317.703	2825.543	1.100503	1.00E-05	PREDICTED: glycine N-methyltransferase-like [Hyalella azteca]
LVAN13473	648.4633	1391.233	1.101267	7.39E-05	hemocyanin subunit L2, partial [Litopenaeus vannamei]
MSTRG.36571	33.69667	72.48667	1.10511	0.014677	regucalcin-like [Penaeus vannamei]
LVAN08416	54.66	118.23	1.113039	9.71E-05	PREDICTED: LOW QUALITY PROTEIN: uncharacterized oxidoreductase YjmC-like [Hyalella azteca]
LVAN08393	2113.317	4607.873	1.124592	5.65E-05	PREDICTED: S-adenosylmethionine synthase-like [Hipposideros armiger]
LVAN14197	144.0767	315.6433	1.131459	0.002634	PREDICTED: uncharacterized protein LOC108677413 [Hyalella azteca]
LVAN07379	12.15	26.83667	1.143249	0.001389	-
LVAN18668	74.41667	164.75	1.146581	0.000137	PREDICTED: dimethylglycine dehydrogenase, mitochondrial-like [Saccoglossus kowalevskii]
MSTRG.33445	2.48	5.533333	1.157809	0.021909	Reverse transcriptase domain, partial [Trinorchestia longiramus]
LVAN11298	21.00333	47.16667	1.167149	1.90E-05	-
LVAN15263	10.38	23.33667	1.168792	0.000692	PREDICTED: 3-hydroxybutyrate dehydrogenase type 2 [Ochotona princeps]
MSTRG.36763	23.81667	53.69333	1.172771	0.002236	uncharacterized protein LOC113827124 [Penaeus vannamei]
LVAN10731	729.7767	1651.6	1.178337	5.83E-08	Lutropin-choriogonadotropic hormone receptor, partial [Penaeus monodon]

LVAN10387	51.44667	116.81	1.183014	7.62E-05	C-type lectin 3 [Fenneropenaeus merguiensis]
MSTRG.19128	9.973333	22.85	1.196046	0.009307	insulin-like growth factor-binding protein-related protein 1 [Penaeus vannamei]
MSTRG.36206	24.16	55.62	1.202983	0.000942	-
MSTRG.5921	74.84	173.6833	1.214578	0.000158	-
LVAN17128	12.29333	29.47	1.261371	3.67E-05	PREDICTED: carotenoid isomerooxygenase-like [Hyalella azteca]
MSTRG.43827	5.54	13.45	1.279648	0.001478	stylicin 1 [Penaeus vannamei]
MSTRG.20197	392.06	953.11	1.281568	1.50E-05	-
MSTRG.10006	45.19667	110.46	1.289236	5.10E-10	poly [ADP-ribose] polymerase 12-like [Penaeus vannamei]
LVAN01543	54.16	134.1933	1.309013	1.03E-09	PREDICTED: putative hydroxypyruvate isomerase [Habropoda laboriosa]
MSTRG.41872	3.89	9.68	1.315237	0.001063	aurora kinase A-like [Penaeus vannamei]
MSTRG.11152	42.22	106.4	1.3335	3.32E-17	phosphoglucomutase [Penaeus vannamei]
LVAN25073	22.85667	57.72333	1.33654	0.008965	PREDICTED: LOW QUALITY PROTEIN: regucalcin-like [Aethina tumida]
MSTRG.31299	3.563333	9.003333	1.337232	0.004582	uncharacterized protein LOC113822930 [Penaeus vannamei]
LVAN05594	7.013333	17.73667	1.338563	0.035735	PREDICTED: sulfotransferase 1A3-like [Hyalella azteca]
MSTRG.249	7.336667	18.65333	1.346237	0.000138	-
LVAN07421	11.88	30.30333	1.350942	0.00783	vitelline membrane outer layer protein I-like protein [Pacifastacus leniusculus]
LVAN08873	25.06667	64.27	1.358375	8.61E-06	PREDICTED: 4-coumarate--CoA ligase 1-like isoform X1 [Branchiostoma belcheri]
LVAN00565	16.45667	42.22	1.359254	7.27E-07	PREDICTED: N(4)-(Beta-N-acetylglucosaminyl)-L-asparaginase-like [Hyalella azteca]
LVAN20568	23.95	61.93	1.370613	8.62E-09	PREDICTED: glycerol-3-phosphate dehydrogenase [NAD(+)], cytoplasmic-like [Limulus polyphemus]
MSTRG.31417	7.186667	18.75667	1.384009	1.91E-05	Plastin-3 [Clonorchis sinensis]
LVAN08303	5.553333	14.54333	1.388932	0.042483	Kazal-type proteinase inhibitor [Litopenaeus vannamei]
LVAN12525	76.82667	202.1233	1.395557	3.82E-08	PREDICTED: zinc metalloproteinase nas-4-like [Pogonomyrmex barbatus]
MSTRG.27819	2.89	7.613333	1.397459	0.04764	DNA ligase 1-like [Penaeus vannamei]
LVAN10725	7.203333	19.04	1.402297	0.01854	PREDICTED: sialin [Fopius arisanus]
MSTRG.14713	6.86	18.34667	1.419237	0.00037	-

MSTRG.43424	3.93	10.57	1.427374	0.021439	small cysteine and glycine repeat-containing protein 2-like [Penaeus vannamei]
LVAN08853	17.27667	46.52667	1.429233	0.007773	PREDICTED: glucose-6-phosphate translocase-like [Parasteatoda tepidariorum]
MSTRG.25896	2.456667	6.63	1.432307	0.002894	polycystic kidney disease protein 1-like 2 [Penaeus vannamei]
MSTRG.11311	39.37667	107.57	1.449863	3.72E-05	trichohyalin-like, partial [Penaeus vannamei]
LVAN15041	2.143333	5.89	1.458411	0.004363	AAEL014436-PA, partial [Aedes aegypti]
MSTRG.4836	205.7733	578.85	1.492134	3.20E-11	-
LVAN18760	1.536667	4.323333	1.49234	0.028543	-
LVAN19026	348.2	979.7033	1.492429	2.14E-09	PREDICTED: cytosolic 10-formyltetrahydrofolate dehydrogenase-like [Limulus polyphemus]
MSTRG.15525	4.52	12.77333	1.49874	3.32E-07	putative glycine N-acyltransferase-like isoform X2 [Penaeus vannamei]
MSTRG.4837	17.94667	51.59333	1.523469	7.45E-08	-
MSTRG.31263	7.34	21.13	1.525441	0.002935	general transcription factor IIH subunit 1-like [Penaeus vannamei]
LVAN05595	20.61333	60.08667	1.543467	0.001195	Sulfotransferase sult [Daphnia magna]
LVAN25452	52.77	154.44	1.549257	4.84E-05	4-aminobutyrate aminotransferase [Daphnia magna]
LVAN19799	28.18333	82.6	1.5513	9.60E-09	PREDICTED: vacuolar protein sorting-associated protein 4B-like [Branchiostoma belcheri]
LVAN12797	23.71667	70.15333	1.56461	5.67E-09	PREDICTED: uncharacterized protein LOC108675418 [Hyalella azteca]
LVAN10580	373.6367	1110.303	1.571246	1.97E-14	ribosomal protein L7 [Penaeus monodon]
LVAN17003	35.66	106.1567	1.573816	0.000528	alpha-I tubulin [Cherax quadricarinatus]
LVAN08767	18.07	55.06667	1.607583	2.83E-10	PREDICTED: monocarboxylate transporter 6-like [Hyalella azteca]
MSTRG.20198	230.08	703.09	1.611574	5.37E-07	-
MSTRG.2555	6.306667	19.27333	1.611657	0.004348	general transcription factor 3C polypeptide 5-like [Penaeus vannamei]
LVAN05181	23.88333	73.64	1.624485	7.12E-07	glycosyl-phosphatidylinositol-linked carbonic anhydrase [Litopenaeus vannamei]
LVAN11504	8.433333	26.13333	1.631716	0.000176	AGAP005188-PA [Anopheles gambiae str. PEST] [Anopheles gambiae]
LVAN18669	715.88	2244.083	1.648337	2.12E-06	PREDICTED: betaine--homocysteine S-methyltransferase 1-like [Hyalella azteca]
MSTRG.27247	0.586667	1.883333	1.682675	0.001612	Peptide deformylase [Penaeus vannamei]
LVAN03353	6.076667	19.54667	1.685571	0.005884	Glucose-6-phosphate translocase, partial [Stegodyphus mimosarum]

MSTRG.10554	3.656667	11.96	1.709616	2.57E-06	-
MSTRG.7592	2.44	8.04	1.720314	0.012424	LOW QUALITY PROTEIN: uridine phosphorylase 1-like [Penaeus vannamei]
LVAN22833	34.22333	115.85	1.759206	0.000137	Retinal dehydrogenase 1 [Zootermopsis nevadensis]
MSTRG.9421	3.406667	11.58	1.765203	0.011005	acetyltransferase, GNAT family [Ancylostoma duodenale]
LVAN22401	37.77667	129.0467	1.772326	3.23E-05	eukaryotic initiation factor 2 subunit alpha [Litopenaeus vannamei]
LVAN23495	7.66	27.43667	1.840689	0.010223	crustacyanin subunit A [Fenneropenaeus merguiensis]
LVAN03242	103.45	370.57	1.840812	3.11E-09	PREDICTED: gamma-butyrobetaine dioxygenase-like [Saccoglossus kowalevskii]
LVAN07292	7.21	26.13333	1.85782	0.000177	PREDICTED: brain protein I3 [Tribolium castaneum]
LVAN23497	10.21667	37.04333	1.858289	0.002375	crustacyanin subunit A [Fenneropenaeus merguiensis]
MSTRG.35205	1.456667	5.303333	1.864229	0.002894	amylase [Penaeus vannamei]
MSTRG.11310	38.29667	145.4367	1.9251	3.10E-15	ankyrin-3-like [Penaeus vannamei]
LVAN01378	3.553333	13.49667	1.925358	1.50E-05	estrogen sulfotransferase [Scylla olivacea]
LVAN13515	0.62	2.42	1.964667	0.04462	Transposon Ty3-G Gag-Pol polyprotein [Trichinella nelsoni]
LVAN14906	4.026667	16.15	2.003876	3.69E-05	3-oxoacyl-[acyl-carrier-protein] reductase [Zootermopsis nevadensis]
MSTRG.31095	0.703333	2.92	2.053688	0.036444	uncharacterized protein LOC113825773 [Penaeus vannamei]
LVAN08195	0.19	0.793333	2.061928	0.043223	PREDICTED: NACHT, LRR and PYD domains-containing protein 3 [Strongylocentrotus purpuratus]
LVAN00603	2.613333	11.02333	2.076597	0.002352	Trimethyllysine dioxygenase, mitochondrial [Daphnia magna]
LVAN09123	2.176667	9.65	2.148408	5.84E-05	-
MSTRG.13222	1.566667	7.363333	2.232661	0.003168	potassium voltage-gated channel protein Shaw-like, partial [Penaeus vannamei]
MSTRG.35981	0.673333	3.363333	2.320499	6.17E-05	keratin, type I cytoskeletal 9-like [Penaeus vannamei]
MSTRG.27864	1.55	7.84	2.338585	0.001721	Peptide deformylase [Penaeus vannamei]
LVAN14209	40.16	206.27	2.360703	0.025479	elongation factor 2 [Litopenaeus vannamei]
MSTRG.19828	0.666667	3.626667	2.443607	0.011772	uncharacterized protein LOC113814189 [Penaeus vannamei]
LVAN09822	12.99667	73.86333	2.506717	2.81E-07	PREDICTED: 40S ribosomal protein S26-like [Hyalella azteca]
MSTRG.23629	1.196667	7.25	2.59896	1.90E-05	-

LVAN23506	0.626667	5.146667	3.037868	0.013023	crustacyanin subunit C [Fenneropenaeus merguiensis]
LVAN20376	0.34	2.803333	3.043537	0.006653	PREDICTED: NAD(P) transhydrogenase, mitochondrial-like [Aplysia californica]
LVAN15807	0.083333	0.71	3.090853	8.69E-05	PREDICTED: flocculation protein FLO11-like [Hyalella azteca]
LVAN02944	0.53	4.636667	3.129024	1.15E-06	Retrovirus-related Pol polyprotein from transposon [Daphnia magna]
LVAN23504	0.406667	3.673333	3.175171	0.019603	crustacyanin subunit A [Fenneropenaeus merguiensis]
LVAN23512	0.443333	4.323333	3.28568	0.018142	crustacyanin A, partial [Penaeus monodon]
MSTRG.41372	0.433333	4.626667	3.416424	2.35E-09	zinc knuckle [Penaeus vannamei]
LVAN08170	0.123333	1.5	3.604328	0.044066	-
LVAN25367	0.263333	3.42	3.699034	0.000579	PREDICTED: alpha-(1,3)-fucosyltransferase C-like [Hyalella azteca]
MSTRG.36990	0.13	1.696667	3.70612	7.55E-06	solute carrier family 22 member 6-A-like [Penaeus vannamei]
LVAN13298	0.383333	10.24	4.739472	2.18E-07	sodium potassium-transporting ATPase subunit beta [Litopenaeus vannamei]
LVAN16590	0.133333	3.683333	4.787903	2.56E-05	PREDICTED: hydroxyacid-oxoacid transhydrogenase, mitochondrial [Sinocyclocheilus rhinocerous]
MSTRG.28833	5.946667	178.3333	4.906351	2.16E-44	reverse transcriptase [Schistosoma japonicum]
LVAN22927	0.016667	0.543333	5.0268	0.042666	PREDICTED: TRPM8 channel-associated factor 3-like [Oryctolagus cuniculus]
LVAN10769	0.083333	8.106667	6.604071	2.25E-05	PREDICTED: putative phospholipase B-like 2 [Hyalella azteca]
LVAN14672	0.001	0.396667	8.631783	0.047054	heat shock protein 21 [Macrobrachium rosenbergii]
LVAN07002	0.226667	105.6733	8.864823	1.50E-29	PREDICTED: S-formylglutathione hydrolase [Crassostrea gigas]
LVAN09731	0.001	0.803333	9.649855	0.008401	oxygenase [Oplophorus gracilirostris]
MSTRG.6632	0.001	1.413333	10.46489	7.94E-07	PREDICTED: prosalusin [Nanorana parkeri]
LVAN22811	0.001	1.793333	10.80843	0.000973	PREDICTED: serine/threonine-protein kinase RIO2 [Solenopsis invicta]
LVAN10931	0.001	2.03	10.98726	0.000474	Protein phosphatase methylesterase 1 [Daphnia magna]
LVAN22623	0.001	8.82	13.10656	3.44E-09	PREDICTED: chromodomain-helicase-DNA-binding protein Mi-2 homolog isoform X4 [Hyalella azteca]

**Metabolite_ID**	**Modes**	**S20507-0h**	**R20523-0h**	**Log2(fc)**	**VIP**	**Description**

M173T281_2	Negative ionization	866157.2	279796.7	-1.63025	5.439919	Gly-Val
M191T346	Negative ionization	147098.7	83426.61	-0.8182	1.852661	Ser-Ser
M216T344	Negative ionization	170646.8	95107.67	-0.84338	1.976112	Ala-Gln
M243T181	Negative ionization	421598.8	207212.6	-1.02476	3.215944	Ile-Leu
M229T192	Negative ionization	2472086	1594215	-0.63288	6.769129	Ile-Val
M339T27	Negative ionization	373752	31133.47	-3.58554	3.886037	Glyceraldehyde, 3-(dihydrogen phosphate)
M131T315	Negative ionization	104722.3	52590.73	-0.99369	1.589462	Gly-Gly
M217T252	Negative ionization	795169.8	419552.5	-0.92241	4.323077	Ser-Leu
M187T249_2	Negative ionization	539399.1	249246.6	-1.11378	3.67717	Val-Ala
M267T291	Negative ionization	128162.8	59036.45	-1.1183	1.798123	Leu-His
M242T76	Negative ionization	130987	15327.04	-3.09527	2.247119	Dimethachlor cga369873
M219T312	Negative ionization	545967.9	378838.2	-0.52723	2.915565	Thr-Thr
M225T352	Negative ionization	137427.4	93244.99	-0.55957	1.343667	L-Carnosine
M457T231	Negative ionization	15721.91	51591.29	1.714351	1.120448	1-(5z,8z,11z,14z-eicosatetraenoyl)-sn-glycero-3-phosphate
M115T399	Negative ionization	71648.83	127213.7	0.828239	1.459056	Fumarate
M201T250_2	Negative ionization	458144.2	258336.7	-0.82655	2.766091	Ala-Ile
M259T354	Negative ionization	1654486	1053488	-0.65121	5.412243	Glu-Leu
M114T302	Negative ionization	2178287	1728097	-0.33401	4.815976	Proline
M246T341	Negative ionization	66228.84	36398.95	-0.86356	1.16099	Gln-thr
M133T402	Negative ionization	67929.78	211621	1.639367	2.297857	Malate
M306T38	Negative ionization	129545.8	52087.61	-1.31445	1.890129	Benzoic acid, 4-[[(5,6,7,8-tetrahydro-5,5,8,8-tetramethyl-2-naphthalenyl)amino]carbonyl]-
M180T292_2	Negative ionization	2294354	1758979	-0.38335	4.732602	N-acetyl-l-tyrosine
M274T449	Negative ionization	92309.79	57250.37	-0.6892	1.14618	Glu-Lys
M324T127	Negative ionization	2004192	1106931	-0.85646	6.406008	Cycloxydime
M179T295_2	Negative ionization	380207.3	131859.1	-1.52779	3.173308	D-(+)-mannose

M187T278	Negative ionization	2836012	2061458	-0.4602	5.955089	Gly-Leu
M255T341	Negative ionization	61275.92	37979.24	-0.69011	1.027603	Thr-His
M468T94_1	Negative ionization	58640.31	31456.46	-0.89854	1.067323	Pyrazino[1',2':1,6]pyrido[3,4-b] indole-3-propanoic acid, 1,2,3,4,6,7,12,12a-octahydro-9-methoxy-6-(2-methylpropyl)- 1,4-dioxo-, 1,1-dimethylethyl ester, (3s,6s,12as)-
M377T29	Negative ionization	260270	683063.1	1.39201	3.950245	L-homocitrulline
M118T344	Negative ionization	677597.3	788567.1	0.218805	1.787536	Threonine
M231T231	Negative ionization	895511.6	582903.1	-0.61946	3.540614	Thr-Leu
M476T187_2	Negative ionization	128987.3	80637.93	-0.6777	1.432641	1-(9z,12z-octadecadienoyl)-2-hydroxy-sn-glycero-3-phosphoethanolamine
M531T111	Negative ionization	105423.8	49607.63	-1.08757	1.464541	Cochlioquinone a
M145T330_2	Negative ionization	349296.3	256824.7	-0.44367	1.967193	Gly-Ala
M544T34	Negative ionization	128109.1	48463.78	-1.40239	1.77012	1-o-hexadecyl-2-o-ethyl-sn-glycero-3-phosphocholine
M306T89	Negative ionization	5004348	3084466	-0.69816	9.266601	Methanone, (4-hydroxyphenyl)(1-pentyl-1h-indol-3-yl)-
M568T33	Negative ionization	167443.7	74284.1	-1.17255	1.908175	1-stearoyl-2-hydroxy-sn-glycero-3-phosphocholine
M265T26_2	Negative ionization	192798.8	263907.9	0.452939	1.44413	Zinniol
M540T192_2	Negative ionization	260400.5	444815.4	0.772475	2.29969	Phosphatidylcholine lyso alkyl 16:0
M243T200	Negative ionization	310870.7	547955.3	0.817743	2.616804	Pseudouridine
M495T25	Negative ionization	52193.56	126167.4	1.273395	1.564211	7.alpha.,17.alpha.-dimethyl-5.beta.-androstane-3.alpha.,17.beta.-diol glucuronide
M363T31	Negative ionization	126448.1	391394.9	1.63008	2.759831	Dhas#18
M322T132	Negative ionization	571559.6	321941.2	-0.82811	3.304105	Anorexigenic peptide
M203T412	Negative ionization	622676.4	534833.1	-0.21939	2.126776	Ala-asp
M339T29	Negative ionization	224328.3	356289.3	0.667437	1.67955	Gly-His-Lys
M429T391	Negative ionization	88568.1	36176.28	-1.29174	1.413934	Ketoprofen .beta.-d-glucuronide
M304T42_1	Negative ionization	1704797	1218879	-0.48405	4.438554	Arg-Met

M191T489	Negative ionization	93896.77	63808.95	-0.55732	1.059371	Citrate
M327T131	Negative ionization	84535.64	46766.68	-0.85408	1.086385	4'-hydroxy-2'-methyl-3,4,5-trimethoxychalcone
M617T235	Negative ionization	975124.6	417999.8	-1.22208	4.170876	7-ethyl-10-(4-n-aminopentanoic acid)-1-piperidino)carbonyloxycamptothecin
M481T29	Negative ionization	178797	108338.4	-0.72278	1.458611	1.alpha.-methyl-5.alpha.-androstan-3.alpha.,17.beta.-diol glucuronide
M349T352	Positive ionization	98739.69	66671.57	-0.56656	1.039277	Amoxicillin
M231T196	Positive ionization	8779196	4762670	-0.88232	11.98796	Val-Ile
M323T399	Positive ionization	479562.4	36883.15	-3.70068	3.770513	Matairesinol
M207T287	Positive ionization	316978.7	200504.9	-0.66075	2.036364	Gly-Met
M249T354_3	Positive ionization	221520.3	119475.4	-0.89073	1.897606	Tanshinone i
M262T345_2	Positive ionization	201069.1	120210.3	-0.74213	1.68358	Tolfenamic acid
M260T366	Positive ionization	1739254	920699.7	-0.91767	5.35041	Lys-Ile
M327T352	Positive ionization	490167.1	331664.9	-0.56355	2.358714	Bilobalide
M243T352	Positive ionization	522783.9	250759.6	-1.05991	3.100413	Ser-His
M926T394	Positive ionization	35941.15	136132.9	1.921308	1.765647	Hoiamide a
M217T409	Positive ionization	760712.5	522408.5	-0.54217	2.915926	Glu-Glu-Arg
M245T183	Positive ionization	1226674	752612.3	-0.70477	3.581594	Leucylleucine
M246T410	Positive ionization	2797257	1970481	-0.50546	4.633645	Arg-Ala
M175T282	Positive ionization	681024.9	278628.7	-1.28936	3.791566	Val-Gly
M184T225	Positive ionization	311928.7	117593.4	-1.40741	2.31758	4-pyridoxic acid
M285T406	Positive ionization	2350775	1859879	-0.33793	3.904862	His-Glu
M304T438	Positive ionization	3372340	1554306	-1.11748	7.075585	Arg-glu
M203T230_2	Positive ionization	879667.1	391451.9	-1.16812	4.178029	Ile-Ala
M261T436	Positive ionization	1003439	755445.5	-0.40955	2.578129	Asn-Lys
M294T326	Positive ionization	235044.2	163248.3	-0.52586	1.463114	Phe-lys
M282T132	Positive ionization	250717	87405.72	-1.52026	2.247244	1-methyladenosine

M558T469	Positive ionization	66788.66	25816.69	-1.3713	1.175336	Penitrem a
M86T231	Positive ionization	268643.4	149104.5	-0.84937	2.042007	1,5-pentanediamine
M363T245	Positive ionization	35963.79	76554.79	1.089948	1.13189	Cinobufotalin
M274T356	Positive ionization	946547.4	625576.4	-0.59749	3.19632	Arg-Val
M552T178	Positive ionization	824357.4	515005.8	-0.67868	3.045147	1-octadecyl-2-acetyl-sn-glycero-3-phosphocholine
M482T189	Positive ionization	1125966	724658.6	-0.63579	3.661014	2-hexadecanoylthio-1-ethylphosphorylcholine
M261T356	Positive ionization	1804944	1201495	-0.58712	4.506228	Ile-Glu
M231T371_2	Positive ionization	6156214	31985921	2.377322	29.37119	Lys-Trp-Lys
M203T361	Positive ionization	83602.93	36094.97	-1.21176	1.291962	Asp-Asp-Arg
M559T415	Positive ionization	119772	223533.6	0.900201	1.929831	Karbutilate
M477T156	Positive ionization	229953.4	124354.2	-0.88689	1.812099	Obacunone
M246T300	Positive ionization	2431268	1511537	-0.68569	5.649565	Val-Gln
M298T420	Positive ionization	228443.1	149611.8	-0.61061	1.60286	L-cysteine-glutathione disulfide
M219T256	Positive ionization	3196727	1875717	-0.76915	6.763018	Val-Thr
M83T336	Positive ionization	298990.5	151198.9	-0.98365	1.85133	4-methyl-1h-pyrazole
M333T452	Positive ionization	281648	145540.3	-0.95247	2.037883	Glu-Gly-Lys
M217T211	Positive ionization	1887623	936914.7	-1.01058	5.7794	Val-Val
M234T354	Positive ionization	322754.3	186820	-0.78879	2.125817	Ser-Gln
M247T370	Positive ionization	3292759	2307569	-0.51292	5.786747	Val-Glu
M264T294	Positive ionization	84701.87	48122.74	-0.81567	1.074971	Met-Asn
M205T287	Positive ionization	882207.5	460799.8	-0.93698	3.847821	Val-Ser
M279T335	Positive ionization	89495.53	17045.45	-2.39243	1.576618	Dibutyl phthalate
M129T336_2	Positive ionization	1767947	867413.4	-1.02728	4.539564	4-ketopimelic acid
M342T367	Positive ionization	167789.9	261376.2	0.639472	1.819835	7-acetyllycopsamine
M463T440	Positive ionization	186279.2	304488	0.708919	2.051879	1-piperazinecarboxylic acid, 4-[(3-phenoxyphenyl)methyl]-, 2,2,2-trifluoro-1-(trifluoromethyl)ethyl ester

M232T305	Positive ionization	347712.6	166739.9	-1.0603	2.466624	Val-Asn
M340T328	Positive ionization	626822.1	895793.7	0.51511	3.109825	Propoxyphene
M170T337_2	Positive ionization	602485.8	1186042	0.977156	4.425605	Diphenylamine
M284T362	Positive ionization	671359.3	284448.2	-1.23892	3.685566	His-Gln
M279T361	Positive ionization	521827.1	346561.8	-0.59046	2.377822	gamma-Glutamyl-L-methionine
M147T300	Positive ionization	183092	128471.8	-0.51112	1.334002	.alpha.-pyrrolidinobutiophenone
M265T353	Positive ionization	188649	128653.3	-0.55222	1.418408	Thiamine
M436T366	Positive ionization	95873.8	145159.3	0.598428	1.244564	Paxilline
M220T361	Positive ionization	222232.6	92888.86	-1.25849	2.114832	Ser-Asn
M391T222	Positive ionization	27428.68	99502.6	1.859049	1.540758	Prostaglandin g2
M317T366	Positive ionization	199080.5	90686.74	-1.13439	1.78451	Leu-Gly-Lys
M369T394	Positive ionization	337145	444819.3	0.39985	1.907692	Hirsutine
M203T254_2	Positive ionization	1022926	588432.4	-0.79775	3.470551	L-alanyl-l-norleucine
M372T388	Positive ionization	25226.82	247418.2	3.293922	2.523839	Tamoxifen
M275T475	Positive ionization	113407.8	58532.62	-0.95421	1.329589	Lys-Gln
M503T444	Positive ionization	79724.49	111427.7	0.483013	1.010687	Crustecdysone
M234T447	Positive ionization	189870.4	125402.7	-0.59845	1.412323	Methylphenidate
M126T290_2	Positive ionization	6756481	9392699	0.475268	9.742971	Taurine
M116T264	Positive ionization	34484.82	72752.86	1.077043	1.127241	3-dehydrocarnitine
M249T199	Positive ionization	873011	585645.2	-0.57597	2.980755	Val-met
M260T284	Positive ionization	2944028	1456619	-1.01517	7.163912	Leu-Gln
M218T429	Positive ionization	1866470	1314525	-0.50577	3.909377	Ala-Lys
M373T230	Positive ionization	96420.78	57435.43	-0.7474	1.100846	Ile-Leu-Lys
M244T359	Positive ionization	670809.3	444906.2	-0.5924	2.693505	Pro-gln
M261T456	Positive ionization	372984.9	219600.4	-0.76424	2.243807	Lys-Asn
M527T417	Positive ionization	122050.9	70710.19	-0.78749	1.244501	Benzoic acid, 2-[3-[3-[(5-ethyl-4'-fluoro-2-hydroxy[1,1'-biphenyl]-4-

						yl)oxy]propoxy]-2-propylphenoxy]-
M504T407	Positive ionization	170756.5	76209.37	-1.1639	1.585068	Glycocholic acid
M204T450_2	Positive ionization	1642578	1378635	-0.25272	2.494567	Gly-Lys
M311T361	Positive ionization	177924.1	112080.6	-0.66673	1.444105	Tyr-Glu
M221T60	Positive ionization	362373.9	176782.6	-1.0355	2.263438	Met-Ala
M367T252	Positive ionization	158196	112868.3	-0.48707	1.187122	Hirsuteine
M401T38	Positive ionization	123856.2	65405.76	-0.92118	1.341569	7.alpha.,27-dihydroxycholesterol
M306T318	Positive ionization	122433.6	82893.77	-0.56266	1.061145	Cytidine 2',3'-cyclic phosphate
M361T365	Positive ionization	137034.9	90635.3	-0.5964	1.161171	Thr-Leu-Lys
M423T245	Positive ionization	52507.56	13583.14	-1.95071	1.141675	Ginsenoside f1
M261T392	Positive ionization	7019670	1787705	-1.97329	12.8419	Gly-Gly-Lys
M290T449	Positive ionization	203531.2	135012.1	-0.59216	1.416858	Arg-asp
M292T252	Positive ionization	180825.4	123946.5	-0.54488	1.307022	Ser-Trp
M276T410	Positive ionization	3110037	1816758	-0.77557	6.130376	Glu-Gln
M247T423	Positive ionization	175976.9	101738	-0.79053	1.3742	2-cis-4-trans-abscisic acid
M161T72	Positive ionization	1075581	607163.7	-0.82496	3.504569	Ala-Ala
M459T233	Positive ionization	41548.22	122335.4	1.557983	1.459798	Fumagillin
M237T276	Positive ionization	202679.1	116819.6	-0.79491	1.654283	Ser-Met
M276T449	Positive ionization	3975612	3036122	-0.38895	4.313947	.gamma.-l-glu-.epsilon.-l-lys
M201T81_2	Positive ionization	2131161	1032895	-1.04495	5.503367	Pro-Glu-Arg
M331T363	Positive ionization	296096.5	223255.4	-0.40737	1.329537	Ala-Leu-Lys
M391T468	Positive ionization	268474.3	178968.8	-0.58508	1.534452	Asp-Glu-Lys
M223T257	Positive ionization	437674.8	284921	-0.6193	2.124723	Phe-gly
M295T404	Positive ionization	60643.91	26293.48	-1.20566	1.070056	Glu-Met(o)
M216T387	Positive ionization	194637.7	80882.39	-1.26689	1.66495	sn-Glycerol 3-phosphoethanolamine
M284T151	Positive ionization	107952	750647.8	2.797746	4.285272	Imazapyr

M309T496	Positive ionization	2992010	1472522	-1.02283	7.104531	Fructoselysine
M255T308	Positive ionization	310040.2	191753.3	-0.6932	1.936112	Val-His
M496T455	Positive ionization	105844.4	41189.14	-1.36161	1.335663	Cytochalasin e
M359T341	Positive ionization	124279.2	190609.6	0.617036	1.454842	Leu-Ala-Arg
M269T292	Positive ionization	8484451	4050173	-1.06684	12.06992	His-Leu
M164T276	Positive ionization	92901.85	42840.72	-1.11672	1.184711	4-hydroxy-l-glutamic acid
M177T257	Positive ionization	92458.64	53797.36	-0.78127	1.095213	1-(1',3'-benzodioxol-5'-yl)-2-butanamine
M369T453	Positive ionization	1045517	333551.7	-1.64823	4.454167	Arg-Pro-Pro
M118T294	Positive ionization	8890112	4694394	-0.92126	10.87616	DL-valine
M319T447	Positive ionization	521377	243414.7	-1.09891	2.964969	Asp-Gly-Lys
M276T239	Positive ionization	113068.3	72941.39	-0.63238	1.002486	Ala-Trp
M346T319_1	Positive ionization	132058.2	89289.63	-0.56461	1.094231	Cyclic gmp
M618T159	Positive ionization	63390.14	120733.4	0.929495	1.238326	Benzoic acid, 3-[[(3-carboxycyclohexyl)amino]carbonyl]-4-[3-[4-[4-(cyclohexyloxy)butoxy]phenyl]propoxy]-
M375T39_1	Positive ionization	72526.26	171357.6	1.240435	1.657925	Prostaglandin f2.alpha.-1-glyceryl ester
M375T366	Positive ionization	578995	417074	-0.47325	2.025797	Thr-Val-Arg
M262T300	Positive ionization	261191	172884.8	-0.59529	1.156434	(2r)-3-hydroxyisovaleroylcarnitine
M354T299	Positive ionization	1186255	1451007	0.290641	2.901189	Proadifen
M219T468	Positive ionization	383300.3	66081.66	-2.53615	3.2047	2-phenylpiperidine-2-acetamide
M140T107	Positive ionization	113893.4	23104.82	-2.30142	1.735839	N-carboxyethyl-.gamma.-aminobutyric acid
M301T446_2	Positive ionization	156332.4	94353.17	-0.72847	1.354012	Ethanone, 1-[1-(4-fluorophenyl)-2,5-dimethyl-1h-pyrrol-3-yl]-2-(1-pyrrolidinyl)-
M230T362	Positive ionization	263294.2	183229.9	-0.52302	1.500992	Pro-asn
M353T481	Positive ionization	219205.1	107561.3	-1.02712	1.852791	Ajmalicine
M496T187_2	Positive ionization	29458748	24212042	-0.28297	11.3133	Lpc 16:0
M769T418	Positive ionization	44522.87	80252.31	0.849996	1.014781	Okadaic acid

M289T421	Positive ionization	307578.3	232408.8	-0.40429	1.446304	Arg-Asn
M705T259	Positive ionization	42878.15	79802.93	0.896199	1.098051	Voacamine
M425T388	Positive ionization	100783.8	168711.2	0.743292	1.32733	His-Leu-Arg
M610T30	Positive ionization	296392.1	169616.5	-0.80523	1.638368	Acetyl-l-carnitine
M254T153	Positive ionization	298690	178276.1	-0.74454	1.643485	Normorphine
M147T333	Positive ionization	331992.2	264811.1	-0.32619	1.509019	Ala-Gly
M233T245	Positive ionization	2660922	1394564	-0.93211	6.020483	Ile-Thr
M337T191	Positive ionization	251086.3	151267.1	-0.73109	1.658079	1-palmitoyl-2-linoleoyl-rac-glycerol
M253T69_1	Positive ionization	1069606	691354.8	-0.62958	3.264708	Nebularine
M655T456	Positive ionization	228819.6	81859.87	-1.48298	1.916367	Coproporphyrin I
M318T362	Positive ionization	518939.6	339941.9	-0.61028	2.239878	Ile-Trp
M381T253	Positive ionization	214877.8	162059.9	-0.40699	1.170793	Methanone, (4-methyl-1-naphthalenyl)[1-[2-(4-morpholinyl)ethyl]-1h-indol-3-yl]-
M187T330	Positive ionization	698094.9	380387.7	-0.87595	3.082275	Pro-Ala
M241T302	Positive ionization	5453296	9350129	0.777858	11.82222	Pheniramine
M243T286	Positive ionization	217829.4	110956.9	-0.9732	1.837594	Molsidomine
M290T476	Positive ionization	170250.9	249048.6	0.548765	1.464581	Asp-Arg
M257T339	Positive ionization	2108391	1419204	-0.57106	4.495322	His-Thr
M231T84	Positive ionization	1011699	563289.3	-0.84483	3.349947	DL-proline
M876T242	Positive ionization	169478.3	235430.7	0.474202	1.387712	3-[(3s,5r,10s,13r,14s,17s)-14-hydroxy-3-[(2r,3s,4s,5s,6s)-3-hydroxy-4-methoxy-6-methyl-5-[(2s,3r,4s,5s,6r)-3,4,5-trihydroxy-6-[[(2r,3r,4s,5s,6r)-3,4,5-trihydroxy-6-(hydroxymethyl)oxan-2-yl]oxymethyl]oxan-2-yl]oxyoxan-2-yl]oxy-10,13-dimethyl-1,2,3,4,5,6,7,8,9,11,12,15,16,17-tetradecahydrocyclopenta[a]phenanthren-17-yl]-2h-furan-5-one
M355T532	Positive ionization	114695.7	65118.47	-0.81667	1.20225	Deoxycorticosterone acetate

M300T40	Positive ionization	1098679	2144991	0.965201	5.333633	Palmitoyl ethanolamide
M582T391	Positive ionization	172426.9	91393.17	-0.91583	1.433284	Ergotamine
M248T337	Positive ionization	670924.9	369309.8	-0.86132	3.053659	Thr-Gln
M387T295	Positive ionization	208234.2	145359.9	-0.51858	1.241697	Bufalin
M404T478	Positive ionization	104142.6	62970.31	-0.72582	1.081162	Ala-Trp-Lys
M348T398	Positive ionization	285257.4	145917	-0.96712	1.665195	Adenosine 2'-monophosphate
M487T440	Positive ionization	132961.9	201650.5	0.600845	1.325891	4-androsten-17.beta.-ol-3-one glucosiduronate
M177T86	Positive ionization	651491	406248.1	-0.68138	2.163792	Ser-Ala
M742T413	Positive ionization	68832.73	137188.9	0.994997	1.301921	1-palmitoyl-2-(4-ketododec-3-enedioyl)phosphatidylcholine
M120T121	Positive ionization	133625.3	80177.57	-0.73692	1.137724	L-.beta.-homoserine
M247T276_1	Positive ionization	166178.3	95897.26	-0.79317	1.429777	Pro-met
M326T40	Positive ionization	115138	184043.2	0.676681	1.352698	N-oleoylethanolamine
M462T174	Positive ionization	65312.85	27102.13	-1.26896	1.08402	Psychosine
M261T365	Positive ionization	167600.2	91787.27	-0.86866	1.36989	Gln-asn
M459T396	Positive ionization	279173.7	493814.7	0.822807	2.471496	Arg-Lys-Arg
M187T406	Positive ionization	552625.5	367811	-0.58734	2.223897	Pyroglutamylglycine
M284T156	Positive ionization	18554.61	101238.5	2.447908	1.49035	Oxymorphone
M468T191	Positive ionization	1624027	1000006	-0.69957	4.32476	1-myristoyl-sn-glycero-3-phosphocholine
M500T396	Positive ionization	181206	114672.5	-0.66011	1.27909	Aconine
M404T439	Positive ionization	264153	181307.4	-0.54294	1.397007	Thr-Gln-Arg
M157T358	Positive ionization	198450.3	105418.9	-0.91264	1.391784	Indole-3-acetonitrile
M478T191	Positive ionization	645997.3	418037	-0.6279	2.634013	4-[5-[[4-[5-[acetyl(hydroxy)amino]pentylamino]-4-oxobutanoyl]-hydroxyamino]pentylamino]-4-oxobutanoic acid
M391T387	Positive ionization	115066.8	70763.16	-0.7014	1.083586	Mitraphylline
M160T381	Positive ionization	503146.3	1158211	1.202849	3.686725	5-aminovaleric acid betaine
M770T232	Positive ionization	265761.3	129850.5	-1.03328	1.85756	2-[2-[19-acetamido-6-(3,4-dicarboxybutanoyloxy)-16,18-dihydroxy-

						5,9-dimethylicosan-7-yl]oxy-2-oxoethyl]butanedioic aci
M247T471	Positive ionization	172914.5	104940.9	-0.72048	1.29828	N-.alpha.-(tert-butoxycarbonyl)-l-lysine
M144T300	Positive ionization	236689.3	162133.4	-0.54581	1.214798	L-homoarginine
M369T541_7	Positive ionization	12619254	9863307	-0.35548	7.263704	Perindopril
M310T294	Positive ionization	171043.1	122551.9	-0.48097	1.187984	N-acetylneuraminate
M387T235	Positive ionization	146994.2	82762.17	-0.82872	1.321573	Val-Ile-Arg
M327T538	Positive ionization	73858.2	37389.6	-0.98212	1.009579	Medroxyprogesterone
M530T158	Positive ionization	573074.7	371957.4	-0.62359	2.304479	(2e,6e,12e)-18-(2,6-dioxopiperidin-4-yl)-9,11-dihydroxy-8-methoxy-10,12,14-trimethyl-15-oxooctadeca-2,6,12-trienoic acid
M268T167	Positive ionization	961274.7	550432.8	-0.80438	3.007365	Adenosine
M146T43	Positive ionization	1284255	782376.8	-0.715	3.417021	Dl-.beta.-homoleucine
M271T409	Positive ionization	547504.6	365123.6	-0.58449	2.331113	His-Asp
M274T66	Positive ionization	7981843	6173012	-0.37075	6.157503	Fenpropidin
M159T120	Positive ionization	176039.4	98924.41	-0.8315	1.355731	Alitame
M90T339	Positive ionization	317936.9	351791.3	0.145979	1.157707	Alanine
M100T62	Positive ionization	234909.2	597316.3	1.346392	2.89452	2-piperidone
M819T140	Positive ionization	2899188	1838374	-0.65722	5.648615	1-(1z-octadecenyl)-2-(4z,7z,10z,13z,16z,19z-docosahexaenoyl)-sn-glycero-3-phosphocholine
M347T409	Positive ionization	350286.8	236742.1	-0.56522	1.613964	Ala-Thr-Arg
M416T367	Positive ionization	196165.1	104930.3	-0.90264	1.252538	Gln-Leu-Arg
M177T120	Positive ionization	116480.2	53929.77	-1.11093	1.19717	Ala-Ser
M558T433	Positive ionization	20729.45	95463.43	2.203266	1.324537	Hexanamide, n-[(1s,2r,3e)-2-hydroxy-1-(hydroxymethyl)-3-heptadecen-1-yl]-6-[(7-nitro-2,1,3-benzoxadiazol-4-yl)amino]-
M246T392	Positive ionization	1626684	1127480	-0.52883	3.650863	Lys-Val
M235T140	Positive ionization	166184.1	97853.03	-0.76409	1.366966	Zolpidem
M118T267_3	Positive ionization	74636516	69147998	-0.11019	8.734132	Betaine

M206T562	Positive ionization	1389049	1096674	-0.34096	2.564658	N-methylparoxetine
M275T363	Positive ionization	180510.7	92619.66	-0.96269	1.584635	Gln-gln
M340T147	Positive ionization	158763.7	114924.9	-0.46619	1.030584	Bursin
M281T220	Positive ionization	372214.7	280315	-0.40909	1.583521	Val-Tyr
M239T297	Positive ionization	478111.1	419289.9	-0.1894	1.081953	Gly-Tyr
M262T411	Positive ionization	972940.1	799262	-0.28368	2.025268	Glu-Asn
M667T444	Positive ionization	119621.8	31089.99	-1.94396	1.30378	Prostaglandin a2-biotin
M545T394	Positive ionization	118231.7	198124.2	0.744788	1.34811	((4r)-4-((3r,5s,7r,9s,10s,12s,13r,14s,17r)-3,7,12-trihydroxy-10,13-dimethylhexadecahydro-1h-cyclopenta[a]phenanthren-17-yl)pentanoyl)glycylglycine
M219T403	Positive ionization	3079415	2740490	-0.16822	3.004462	5-L-Glutamyl-L-alanine
M233T377	Positive ionization	1577406	1247761	-0.33821	3.021604	Val-Asp
M232T433	Positive ionization	5958153	5089360	-0.22738	3.847853	Gly-Arg
M779T146	Positive ionization	248961	151460.1	-0.71698	1.469318	1,2-di-(9z,12z,15z-octadecatrienoyl)-sn-glycero-3-phosphocholine
M227T337	Positive ionization	606608.5	282598.2	-1.10201	3.003793	L-carnosine
M359T254	Positive ionization	348098.2	270411.9	-0.36434	1.354372	Cannabidiolic acid
M219T113	Positive ionization	135302.6	70214.62	-0.94635	1.198442	Eseroline
M921T377	Positive ionization	210644.4	422883.9	1.005452	2.278355	Stachybocin c
M304T377	Positive ionization	431304.5	271989.5	-0.66516	1.857134	Val-Trp
M310T362	Positive ionization	158722.6	102076.7	-0.63685	1.199319	Tyr-Lys
M666T396	Positive ionization	257214.1	446316.2	0.795096	2.196168	Buprenorphine glucuronide
M288T202	Positive ionization	372047.4	162357.2	-1.19631	2.23175	Octanoylcarnitine
M229T298	Positive ionization	10167050	6696728	-0.60237	9.423138	Pro-leu
M249T396	Positive ionization	4474023	4120797	-0.11865	2.395138	Thr-Glu
M246T237	Positive ionization	731249.8	335357	-1.12467	2.78713	2-methylbutyryl-l-carnitine
M361T451	Positive ionization	168487.2	246353.5	0.548091	1.497071	Arg-Trp

M388T427_2	Positive ionization	85532.49	41957.68	-1.02754	1.01627	4-hydroxytamoxifen
M132T339	Positive ionization	192109	66379.05	-1.53313	1.611223	Hydroxyproline
M218T277	Positive ionization	2531225	1306081	-0.95459	5.243351	L-propionylcarnitine
M237T229	Positive ionization	404446.5	336422.5	-0.26567	1.187205	Ala-phe
M359T238	Positive ionization	1812982	1097280	-0.72443	3.563736	Butaprost (free acid)
M283T73	Positive ionization	812144.1	638358.8	-0.34737	1.89432	Tyr-Thr
M302T242	Positive ionization	1351518	1037913	-0.3809	2.775413	Leu-Gly-Leu
M328T510	Positive ionization	147320.3	79990.07	-0.88106	1.38945	Piperidine, 4-(5h-dibenzo[a,d]cyclohepten-5-ylidene)-1-[4-(2h-tetrazol-5-yl)butyl]-
M316T191	Positive ionization	152211.3	61362.94	-1.31064	1.418861	Decanoyl-l-carnitine
M331T399	Positive ionization	280851.7	167900.1	-0.74221	1.749219	Gly-Val-Arg
M357T534	Positive ionization	181343.6	101950.7	-0.83085	1.438927	.beta.-estradiol 17-valerate
M116T305_2	Positive ionization	7863675	6965035	-0.17507	4.798673	D-ornithine
M473T35	Positive ionization	238310.7	139596.3	-0.77158	1.80205	.alpha.-tocopheryl acetate
M290T277	Positive ionization	302795.5	251363.8	-0.26857	1.115763	Tetrahydropiperine
M367T463	Positive ionization	950142.1	1927612	1.020599	4.074503	Phosphocholine
M293T456	Positive ionization	74765.3	116515	0.640075	1.05533	Ethylenediaminetetraacetic acid
M463T424	Positive ionization	150370.9	96503.53	-0.63987	1.145876	2-naphthalenecarboxamide, n-[(1s)-2-[4-(5-chloro-2,3-dihydro-2-oxo-1h-benzimidazol-1-yl)-1-piperidinyl]-1-methylethyl]-
M646T396	Positive ionization	117382.6	185985.5	0.663972	1.16456	Apicidin
M731T148	Positive ionization	222266	104949.3	-1.0826	1.687307	1,2-dipalmitoleoyl-sn-glycero-3-phosphocholine
M207T330	Positive ionization	810903.1	693960	-0.22468	1.839604	Ser-Thr
M480T196	Positive ionization	1990915	3471784	0.802246	6.420766	1-(1z-hexadecenyl)-sn-glycero-3-phosphocholine
M329T427	Positive ionization	52731.22	113181.1	1.101903	1.099332	Pro-Gly-Arg
M302T405	Positive ionization	376008.1	263136.7	-0.51495	1.593712	Flutriafol
M148T390_2	Positive ionization	3507172	3815029	0.121386	3.370789	DL-Glutamic acid

M104T68	Positive ionization	414270.9	212419.3	-0.96366	1.895795	3-aminobutanoic acid
M263T39	Positive ionization	812020.5	644446.9	-0.33345	2.115931	2-linoleoylglycerol
M136T159	Positive ionization	176520.4	103574.9	-0.76916	1.277955	Adenine
M520T186_2	Positive ionization	57969733	47265147	-0.29452	16.43807	Lpc 18:2
