# Supplementary material for: Integrated application of transcriptomics and metabolomics provides insights into acute hepatopancreatic necrosis disease resistance of Pacific white shrimp Litopenaeus vannamei
Source: mSystems. 2023 Jun 26;8(4):e00067-23. doi: 10.1128/msystems.00067-23 (PMC10469596; doi:10.1128/msystems.00067-23)
Supplement: TABLE S4 — DEGs and DMs between resistant and susceptible families after AHPND infection. [file msystems.00067-23-s0008.pdf]

Table S4. DEGs and DMs between resistant and susceptible families after AHPND infection

Gene_ID	S20507-12h	R20523-12h	log2(fc)	FDR	Description
LVAN07002	0.42	115.1633	8.099076	3.39E-51	PREDICTED: S-formylglutathione hydrolase [Crassostrea gigas]
MSTRG.28833	5.206667	134.0733	4.686518	6.04E-32	reverse transcriptase [Schistosoma japonicum]
MSTRG.11152	36.27667	128.4967	1.824617	8.07E-23	phosphoglucomutase [Penaeus vannamei]
LVAN09822	16.40667	110.86	2.756385	5.37E-18	PREDICTED: 40S ribosomal protein S26-like [Hyalella azteca]
LVAN20377	0.106667	20.37	7.577193	1.64E-15	NAD(P) transhydrogenase, mitochondrial [Daphnia magna]
LVAN12995	3.006667	35.77	3.572513	2.02E-14	-
LVAN07652	778.9467	298.2233	-1.38513	2.02E-14	chitinase 1 precursor [Litopenaeus vannamei]
LVAN12493	0.816667	14.29667	4.129788	3.04E-14	PREDICTED: glutamate receptor ionotropic, kainate 4-like [Hyalella azteca]
MSTRG.37237	1.193333	0.05	-4.57693	2.81E-13	hypothetical protein AS27_10936, partial [Aptenodytes forsteri]
MSTRG.11310	30.08333	123.6667	2.03942	4.24E-13	ankyrin-3-like [Penaeus vannamei]
LVAN07473	56.09	18.76667	-1.57957	2.85E-12	D-beta-hydroxybutyrate dehydrogenase, mitochondrial [Daphnia magna]
MSTRG.30331	3.053333	0.001	-11.5762	3.99E-12	-
LVAN10580	301.6833	1028.473	1.769397	6.82E-12	ribosomal protein L7 [Penaeus monodon]
LVAN01599	38.04	9.056667	-2.07047	1.94E-11	PREDICTED: gamma-glutamyl hydrolase-like [Galendromus occidentalis]
LVAN15198	81.87333	37.38333	-1.131	1.94E-11	PREDICTED: sphingomyelin phosphodiesterase-like [Diachasma alloeum]
MSTRG.35191	700.5433	286.5333	-1.28977	2.97E-11	keratin-associated protein 16-1-like [Penaeus vannamei]
MSTRG.10554	1.27	14.51333	3.514479	6.09E-11	-
LVAN14202	34.23667	12.16667	-1.49261	7.56E-11	Zinc/iron regulated transporter-related protein 42C.2 [Daphnia magna]
LVAN00007	15.82	6.036667	-1.38993	8.16E-11	PI-PLC X domain-containing protein [Daphnia magna]
LVAN14777	43.84	141.6933	1.692452	8.85E-11	PREDICTED: methionine synthase [Apteryx australis mantelli]
LVAN22623	0.001	12.41667	13.59999	4.14E-10	PREDICTED: chromodomain-helicase-DNA-binding protein Mi-2 homolog isoform X4 [Hyalella azteca]
MSTRG.31652	0.49	12.18333	4.635983	1.04E-09	-

LVAN10211	29.01	225.95	2.961381	1.19E-09	PREDICTED: lactoylglutathione lyase [Crassostrea gigas]
LVAN23560	53.82	21.33	-1.33526	1.49E-09	PREDICTED: ectonucleotide pyrophosphatase/phosphodiesterase family member 6-like [Parasteatoda tepidariorum]
MSTRG.10006	40.65667	107.52	1.403041	1.81E-09	poly [ADP-ribose] polymerase 12-like [Penaeus vannamei]
MSTRG.27439	11.58667	1.186667	-3.28748	2.60E-09	chitinase [Penaeus vannamei]
MSTRG.41094	7.473333	0.23	-5.02205	3.75E-09	-
LVAN10880	0.153333	2.833333	4.207757	4.16E-09	Retrovirus-related Pol polyprotein from transposon 412-like Protein [Tribolium castaneum]
LVAN02944	0.47	4.17	3.149315	5.43E-09	Retrovirus-related Pol polyprotein from transposon [Daphnia magna]
LVAN07651	504.2667	219.9567	-1.19697	7.83E-09	chitinase 1 precursor [Litopenaeus vannamei]
LVAN13548	186.1033	67.53667	-1.46236	7.83E-09	beta-1,3-glucan-binding protein precursor [Astacus astacus]
LVAN22415	813.2067	1871.32	1.202362	1.01E-08	PREDICTED: adenosylhomocysteinase-like isoform X1 [Hyalella azteca]
MSTRG.31649	0.12	14.59333	6.926131	1.43E-08	-
LVAN14972	38.27667	15.34	-1.31917	1.66E-08	PREDICTED: ectonucleotide pyrophosphatase/phosphodiesterase family member 6 isoform X2 [Cynoglossus semilaevis]
MSTRG.7585	16.05667	1.52	-3.40103	2.08E-08	-
LVAN23455	21.55667	100.1767	2.21634	3.00E-08	thioredoxin 1 [Litopenaeus vannamei]
LVAN06160	143.98	60.01	-1.26259	5.50E-08	PREDICTED: UDP-glucuronosyltransferase 2B19-like isoform X1 [Hyalella azteca]
LVAN17003	48.73333	192.11	1.978952	5.50E-08	alpha-I tubulin [Cherax quadricarinatus]
LVAN22807	109.3733	42.4	-1.36712	5.50E-08	Peroxiredoxin-6 [Lepeophtheirus salmonis]
MSTRG.16167	0.166667	5.766667	5.1127	6.77E-08	-
LVAN11304	49.06	17.27	-1.50628	8.72E-08	chitin binding-like protein [Fenneropenaeus chinensis]
MSTRG.36985	13.56	4.536667	-1.57965	1.06E-07	PREDICTED: uncharacterized oxidoreductase C26H5.09c-like [Linepithema humile]
MSTRG.41372	0.423333	4.093333	3.27341	1.12E-07	zinc knuckle [Penaeus vannamei]
MSTRG.20369	0.433333	6.733333	3.957772	1.19E-07	-
MSTRG.25896	1.706667	10.39	2.605942	1.59E-07	polycystic kidney disease protein 1-like 2 [Penaeus vannamei]
LVAN13545	631.1467	292.31	-1.11048	2.26E-07	-

MSTRG.9281	0.073333	37.02667	8.97988	2.58E-07	-
MSTRG.41759	0.173333	2.953333	4.090723	2.63E-07	-
MSTRG.31263	3.44	18.1	2.395509	2.96E-07	general transcription factor IIH subunit 1-like [Penaeus vannamei]
MSTRG.33445	0.616667	4.276667	2.793924	3.15E-07	Reverse transcriptase domain, partial [Trinorchestia longiramus]
LVAN22771	74.20333	12.11	-2.61529	3.18E-07	hemocyanin V4 [Litopenaeus vannamei]
LVAN17437	43.20333	125	1.532714	3.25E-07	PREDICTED: tubulin-folding cofactor B-like [Hyalella azteca]
MSTRG.34811	159.5367	60.70333	-1.39404	3.25E-07	uncharacterized protein LOC113807558 [Penaeus vannamei]
MSTRG.39528	74.14	240.6	1.698313	4.03E-07	metallothionein, partial [Penaeus monodon]
LVAN13547	78.83667	29.93333	-1.39711	4.34E-07	Microsomal triglyceride transfer protein large subunit [Daphnia magna]
LVAN13298	0.503333	8.97	4.155522	5.25E-07	sodium potassium-transporting ATPase subunit beta [Litopenaeus vannamei]
MSTRG.31794	1.366667	12.25	3.164048	5.55E-07	phenoloxidase-activating factor 1-like [Penaeus vannamei]
MSTRG.41017	67.99	27.44667	-1.30869	6.58E-07	myrosinase-binding protein 2-like [Penaeus vannamei]
LVAN09956	7.74	0.746667	-3.3738	8.73E-07	Glucosylceramidase precursor [Daphnia magna]
MSTRG.249	5.873333	22.45667	1.934892	1.38E-06	-
MSTRG.31299	4.143333	14.94667	1.85096	1.69E-06	uncharacterized protein LOC113822930 [Penaeus vannamei]
LVAN03811	0.001	5.323333	12.37811	1.73E-06	triosephosphate isomerase [Penaeus monodon]
MSTRG.31849	12.25333	45.73667	1.900177	1.75E-06	-
LVAN13483	20.96	8.893333	-1.23684	1.75E-06	PREDICTED: sphingomyelin phosphodiesterase-like [Hyalella azteca]
LVAN22127	10.81	40.73667	1.913961	2.82E-06	Kruppel-like protein, partial [Scylla paramamosain]
LVAN14888	113.8233	53.66667	-1.0847	4.53E-06	prophenoloxidase 3 [Fenneropenaeus chinensis]
LVAN10879	0.756667	4.156667	2.457697	4.89E-06	Retrovirus-related Pol polyprotein from transposon [Daphnia magna]
LVAN08431	5.086667	0.696667	-2.86818	5.99E-06	vitellogenin [Metapenaeus ensis]
LVAN09046	0.253333	6.823333	4.751368	6.14E-06	PREDICTED: uncharacterized protein LOC108674387 [Hyalella azteca]
LVAN04990	2.51	72.85667	4.859302	6.39E-06	PREDICTED: filaggrin-2-like isoform X3 [Hyalella azteca]
MSTRG.252	0.001	1.233333	10.26835	7.06E-06	-
LVAN05761	78.37667	35.59667	-1.13868	8.53E-06	PREDICTED: uncharacterized protein LOC108676292 [Hyalella azteca]

LVAN08651	0.46	62.81333	7.093293	8.54E-06	caspase [Eriocheir sinensis]
LVAN12131	3077.343	1510.18	-1.02696	8.92E-06	cathepsin l [Litopenaeus vannamei]
LVAN19879	91.25333	227.29	1.316585	8.92E-06	juvenile hormone esterase-like carboxylesterase 1 [Eriocheir sinensis]
MSTRG.35540	18.43667	6.106667	-1.59412	9.12E-06	titin-like [Penaeus vannamei]
LVAN11298	23.15667	58.02	1.325123	9.60E-06	-
LVAN13356	646.0767	302.4867	-1.09483	1.06E-05	Alpha-N-acetylgalactosaminidase [Harpegnathos saltator]
LVAN16590	0.096667	4.713333	5.607585	1.14E-05	PREDICTED: hydroxyacid-oxoacid transhydrogenase, mitochondrial [Sinocyclocheilus rhinocerous]
LVAN09123	1.553333	7.893333	2.345267	1.41E-05	-
LVAN23498	6.76	0.001	-12.7228	1.44E-05	crustacyanin subunit C [Fenneropenaeus merguiensis]
LVAN22401	32.94667	155.11	2.235087	1.44E-05	eukaryotic initiation factor 2 subunit alpha [Litopenaeus vannamei]
LVAN16792	28.1	9.45	-1.57218	1.73E-05	RFS2 protein [Daphnia magna]
MSTRG.640	21.58667	8.576667	-1.33165	1.73E-05	neuroparsin-A-like [Penaeus vannamei]
MSTRG.14311	1.876667	9.396667	2.323977	1.90E-05	hypothetical protein C7M84_008441 [Penaeus vannamei]
LVAN08497	9.033333	0.726667	-3.63589	1.92E-05	PREDICTED: slit homolog 3 protein, partial [Dasypus novemcinctus]
MSTRG.20839	18.07667	41.06333	1.183722	2.11E-05	RNA-directed DNA polymerase from mobile element jockey [Araneus ventricosus]
LVAN07292	5.836667	23.01667	1.979462	2.11E-05	PREDICTED: brain protein I3 [Tribolium castaneum]
MSTRG.18966	1.17	12.17	3.378749	2.27E-05	uncharacterized protein LOC113813488 [Penaeus vannamei]
LVAN03473	1.073333	13.13667	3.613429	2.84E-05	PREDICTED: uncharacterized protein LOC108669104 [Hyalella azteca]
LVAN13546	425.5767	211.9333	-1.00581	2.92E-05	Vitellogenin [Melipona quadrifasciata]
LVAN18520	2035.243	987.3167	-1.04362	2.97E-05	chitinase precursor [Litopenaeus vannamei]
LVAN03810	0.686667	12.05	4.133279	3.32E-05	triosephosphate isomerase [Penaeus monodon]
MSTRG.5205	28.58667	11.32667	-1.33562	3.71E-05	chymotrypsin BII-like [Penaeus vannamei]
MSTRG.27803	2.906667	19.73667	2.763441	3.95E-05	waprin-Phi3-like [Penaeus vannamei]
MSTRG.9467	50.64	21.11667	-1.2619	4.19E-05	filamin-A-like [Penaeus vannamei]
LVAN01162	6.396667	1.953333	-1.71138	4.84E-05	PREDICTED: platelet glycoprotein Ib alpha chain [Aotus nancymaae]

MSTRG.6507	0.63	103.7533	7.36359	5.01E-05	caspase [Penaeus vannamei]
LVAN08434	2.986667	0.293333	-3.34792	5.92E-05	vitellogenin [Metapenaeus ensis]
MSTRG.30068	4.593333	12.03333	1.389423	5.98E-05	NFX1-type zinc finger-containing protein 1-like [Penaeus vannamei]
LVAN08647	0.42	44.11667	6.714791	6.11E-05	caspase [Eriocheir sinensis]
LVAN08436	4.073333	0.54	-2.91518	6.40E-05	vitellogenin [Metapenaeus ensis]
LVAN08650	0.156667	15.58667	6.63647	7.08E-05	caspase [Eriocheir sinensis]
LVAN17270	1291.713	565.5933	-1.19145	7.57E-05	trypsin [Litopenaeus vannamei]
MSTRG.19834	67.23667	23.76	-1.50071	7.77E-05	-
LVAN14747	68.08333	212.76	1.643853	7.84E-05	PREDICTED: methylenetetrahydrofolate reductase-like [Hyalella azteca]
MSTRG.36992	11.93667	4.163333	-1.51959	8.32E-05	solute carrier family 22 member 6-A-like [Penaeus vannamei]
LVAN18690	162.3667	393.33	1.276485	8.75E-05	PREDICTED: UDP-glucose 4-epimerase-like isoform X2 [Hyalella azteca]
MSTRG.27864	1.213333	7.12	2.552901	9.08E-05	Peptide deformylase [Penaeus vannamei]
LVAN13516	1.75	10.50333	2.58542	9.26E-05	Retrovirus-related Pol polyprotein from transposon [Daphnia magna]
MSTRG.1087	0.76	6.58	3.114016	9.57E-05	uncharacterized protein LOC113814559 [Penaeus vannamei]
LVAN13101	15.68667	3.046667	-2.36424	9.94E-05	chitin binding-like protein [Fenneropenaeus chinensis]
MSTRG.31079	183.35	68.00667	-1.43085	0.000113	-
LVAN22811	0.001	2.78	11.44087	0.000121	PREDICTED: serine/threonine-protein kinase RIO2 [Solenopsis invicta]
LVAN00449	388.9967	146.81	-1.40581	0.000124	PREDICTED: very low-density lipoprotein receptor isoform X2 [Microplitis demolitor]
LVAN10769	0.136667	6.583333	5.590085	0.000125	PREDICTED: putative phospholipase B-like 2 [Hyalella azteca]
LVAN01613	4.103333	1.16	-1.82267	0.000127	PREDICTED: endothelin-converting enzyme 1-like [Hyalella azteca]
MSTRG.30088	12.18333	3.733333	-1.70637	0.000133	gastrula zinc finger protein XlCGF57.1-like [Danio rerio]
LVAN10648	0.443333	3.723333	3.070131	0.000134	PREDICTED: peroxisomal N(1)-acetyl-spermine/spermidine oxidase [Camponotus floridanus]
LVAN16587	1.136667	0.001	-10.1506	0.00015	Oxysterol-binding protein-related protein 10 [Columba livia]
LVAN20600	2.863333	0.47	-2.60696	0.00015	Esterase FE4 [Orchesella cincta]
LVAN11299	8.673333	22.62	1.38294	0.00015	ankyrin [Orbicella faveolata]

MSTRG.32108	14.97667	7.353333	-1.02625	0.000154	coiled-coil domain-containing protein 58-like [Penaeus vannamei]
LVAN11759	129.7467	479.0633	1.884519	0.000156	fructose 1,6-biphosphate-aldolase A [Fenneropenaeus chinensis]
LVAN17702	1.056667	39.17667	5.212402	0.000156	triosephosphate isomerase [Penaeus monodon]
LVAN06425	0.89	12.33667	3.793003	0.00016	-
LVAN24174	0.466667	3.493333	2.90414	0.00016	PREDICTED: 1-aminocyclopropane-1-carboxylate synthase-like protein 1 isoform X2 [Lingula anatina]
MSTRG.10569	10.37667	40.40667	1.96125	0.00016	-
LVAN00911	5.11	1.65	-1.63086	0.000161	Nose resistant to fluoxetine protein 6, partial [Stegodyphus mimosarum]
MSTRG.25712	9.326667	2.126667	-2.13277	0.000162	-
LVAN08433	3.67	0.503333	-2.86619	0.000168	vitellogenin [Metapenaeus ensis]
LVAN11053	1.406667	61.50333	5.450312	0.000168	caspase [Eriocheir sinensis]
MSTRG.1596	1.606667	5.696667	1.826047	0.000168	-
MSTRG.25451	8.216667	2.65	-1.63256	0.000181	uncharacterized protein LOC113818508 [Penaeus vannamei]
MSTRG.40979	6.756667	19.38	1.520185	0.000181	thioredoxin 1 [Penaeus vannamei]
LVAN04981	1.873333	54.21333	4.854968	0.000194	PREDICTED: filaggrin-2-like isoform X3 [Hyalella azteca]
MSTRG.39654	64.89333	167.0433	1.36408	0.00022	-
LVAN08146	20.80333	93.76	2.172158	0.000227	serine proteinase inhibitor [Litopenaeus vannamei]
LVAN08223	17.64333	6.88	-1.35864	0.000234	Brix domain-containing protein 1 [Zootermopsis nevadensis]
LVAN19965	115.74	57.80333	-1.00166	0.000252	PREDICTED: arylsulfatase B-like [Branchiostoma belcheri]
LVAN17385	11.11	4.953333	-1.16539	0.000258	AAEL014668-PA, partial [Aedes aegypti]
MSTRG.30303	138.3867	301.7367	1.124585	0.00026	solute carrier family 15 member 2-like isoform X3 [Penaeus vannamei]
MSTRG.34761	32.02667	92.61	1.531894	0.00027	uncharacterized protein LOC113825568 [Penaeus vannamei]
LVAN20409	98.21333	35.83	-1.45475	0.000285	hemolymph clottable protein [Litopenaeus vannamei]
LVAN08646	0.876667	38.24	5.44691	0.000287	caspase [Eriocheir sinensis]
MSTRG.35752	3.136667	9.326667	1.572129	0.000293	-
LVAN04508	1.126667	37.93333	5.073334	0.000304	triosephosphate isomerase [Penaeus monodon]

LVAN11055	2.216667	11.02667	2.314533	0.00031	PREDICTED: solute carrier family 28 member 3-like [Hyalella azteca]
LVAN12117	13.49	79.77	2.563956	0.000365	anti-lipopolysaccharide factor [Litopenaeus stylirostris]
MSTRG.2528	1.786667	0.246667	-2.85664	0.000371	-
MSTRG.27247	0.456667	1.763333	1.949092	0.000383	Peptide deformylase [Penaeus vannamei]
MSTRG.15566	4.433333	23.02	2.376424	0.000396	uncharacterized protein LOC113810921 [Penaeus vannamei]
LVAN04355	34.07	79.86333	1.229031	0.000397	sodium-dependent phosphate transporter [Daphnia pulex]
LVAN00215	4.786667	22.45	2.229622	0.000407	Brain chitinase and chia [Daphnia magna]
MSTRG.33548	16.17	37.71667	1.221882	0.000423	beta-1,4-N-acetylgalactosaminyltransferase bre-4-like [Penaeus vannamei]
MSTRG.30359	53.02667	24.92667	-1.08903	0.000423	PREDICTED: clumping factor A [Sinocyclocheilus rhinocerous]
LVAN12420	2.853333	0.776667	-1.87728	0.000423	transient receptor potential cation channel pyrexia [Homarus americanus]
MSTRG.12361	0.026667	3.256667	6.932215	0.00043	-
LVAN08488	21.96333	8.666667	-1.34155	0.000431	PREDICTED: alpha-mannosidase 2C1-like [Neolamprologus brichardi]
MSTRG.37481	0.29	9.103333	4.97227	0.000435	-
MSTRG.2842	4.526667	13.64	1.591323	0.000446	-
LVAN08432	1.713333	0.063333	-4.7577	0.000466	vitellogenin [Fenneropenaeus chinensis]
LVAN02341	55.02667	145.48	1.402618	0.000471	trypsin [Euphausia superba]
MSTRG.32408	1.96	4.546667	1.213956	0.000479	Transmembrane protein C9orf91-like protein [Armadillidium vulgare]
LVAN11052	0.046667	6.586667	7.141012	0.000489	caspase [Eriocheir sinensis]
LVAN10931	0.001	2.273333	11.15059	0.000508	Protein phosphatase methylesterase 1 [Daphnia magna]
LVAN16375	0.346667	6.1	4.137188	0.000517	PREDICTED: methyltransferase-like protein 24 [Biomphalaria glabrata]
MSTRG.36759	51.87333	134.48	1.374327	0.000551	uncharacterized protein LOC113827125 [Penaeus vannamei]
MSTRG.25653	5.793333	0.08	-6.17825	0.000569	-
MSTRG.4788	0.55	6.063333	3.462608	0.000579	uncharacterized protein LOC113803140 [Penaeus vannamei]
MSTRG.38015	2.893333	10.21667	1.82012	0.000599	organic solute transporter subunit alpha-like [Penaeus vannamei]
MSTRG.27179	0.001	2.586667	11.33688	0.000605	-
MSTRG.38122	15.55	31.35667	1.011858	0.000648	nicotinamide phosphoribosyltransferase-like [Penaeus vannamei]

LVAN15657	93.35667	39.32667	-1.24725	0.000835	Calcium-activated chloride channel regulator 4, 30 kDa form [Daphnia magna]
LVAN00452	8.036667	2.383333	-1.75362	0.000835	-
MSTRG.5266	2.1	11.50333	2.453591	0.000853	-
LVAN19880	14.61333	29.82333	1.029156	0.000856	juvenile hormone esterase-like carboxylesterase 1 [Eriocheir sinensis]
MSTRG.11311	43.21	110.15	1.350032	0.000875	trichohyalin-like, partial [Penaeus vannamei]
LVAN00565	14.65333	33.50667	1.193219	0.000896	PREDICTED: N(4)-(Beta-N-acetylglucosaminyl)-L-asparaginase-like [Hyalella azteca]
MSTRG.15937	13.7	35.69	1.381344	0.00105	ras-related protein Rac1-like [Penaeus vannamei]
LVAN13733	101.9833	47.42333	-1.10466	0.001075	PREDICTED: cytochrome P450 9e2-like [Hyalella azteca]
LVAN23334	8.37	22.36667	1.418051	0.001093	PREDICTED: beta-ureidopropionase-like [Priapulus caudatus]
MSTRG.35844	370.1433	161.8033	-1.19384	0.001151	zinc proteinase [Astacus astacus]
MSTRG.13636	6.526667	2.966667	-1.1375	0.001151	-
LVAN16241	57.25667	26.47333	-1.1129	0.001204	-
MSTRG.35523	9.51	20.66	1.119323	0.001269	Mariner Mos1 transposase [Cryptotermes secundus]
LVAN17704	0.533333	27.49	5.687726	0.001269	triosephosphate isomerase [Penaeus monodon]
MSTRG.14713	8.03	29.58667	1.881475	0.001276	-
MSTRG.36579	2.12	0.366667	-2.53152	0.001326	uncharacterized protein LOC113826983 [Penaeus vannamei]
LVAN04982	1.636667	40.02	4.611889	0.001348	PREDICTED: filaggrin-2-like isoform X3 [Hyalella azteca]
MSTRG.15525	4.003333	11.10667	1.472152	0.001453	putative glycine N-acyltransferase-like isoform X2 [Penaeus vannamei]
LVAN23240	8.176667	3.746667	-1.12591	0.001472	toll2 [Litopenaeus vannamei]
LVAN07382	6.246667	14.84333	1.248657	0.001472	PREDICTED: probable phosphoserine aminotransferase [Drosophila elegans]
LVAN22770	1409.32	536.4567	-1.39347	0.001487	hemocyanin [Litopenaeus vannamei]
MSTRG.41074	0.001	2.473333	11.27224	0.001511	uncharacterized protein LOC113830405 [Penaeus vannamei]
LVAN18819	0.506667	3.586667	2.823535	0.001511	serine protease [Scylla paramamosain]
LVAN22815	11.27333	23.88667	1.083291	0.001511	-
MSTRG.31417	6.396667	17.41667	1.445076	0.001511	Plastin-3 [Clonorchis sinensis]
MSTRG.13209	2.403333	6.403333	1.413786	0.001546	E3 ubiquitin-protein ligase SIAH1B-like [Penaeus vannamei]

LVAN07379	10.91	28.63667	1.392212	0.0017	-
MSTRG.6643	19.47333	9.706667	-1.00445	0.001705	phosphoglucomutase [Penaeus vannamei]
LVAN09041	0.14	3.096667	4.467217	0.001705	PREDICTED: ETS-related transcription factor Elf-4-like [Hyalella azteca]
LVAN21223	95.38333	47.24	-1.01373	0.001775	PREDICTED: cytochrome P450 9e2-like [Hyalella azteca]
MSTRG.31532	87	33.89667	-1.35987	0.001906	uncharacterized protein LOC113823073 isoform X1 [Penaeus vannamei]
LVAN21839	0.27	2.65	3.294961	0.001924	PREDICTED: arrestin homolog [Hyalella azteca]
LVAN17854	0.036667	2.456667	6.066089	0.002058	PREDICTED: pentraxin-related protein PTX3-like [Hyalella azteca]
LVAN23506	2.5	15.96333	2.674762	0.002062	crustacyanin subunit C [Fenneropenaeus merguiensis]
LVAN00797	10.2	4.176667	-1.28815	0.002178	Sodium-coupled neutral amino acid transporter [Daphnia magna]
LVAN06308	4.146667	11.15	1.42702	0.002183	-
LVAN13864	15.17667	30.58667	1.011048	0.002281	PREDICTED: GDP-Man:Man(3)GlcNAc(2)-PP-Dol alpha-1,2-mannosyltransferase isoform X2 [Strongylocentrotus purpuratus]
MSTRG.12363	0.07	17.98	8.004822	0.00237	-
LVAN10013	6.153333	14.22667	1.209158	0.002375	Calpain-7 [Zootermopsis nevadensis]
MSTRG.28446	5.71	16.67333	1.54598	0.002427	poly [ADP-ribose] polymerase 3-like [Penaeus vannamei]
LVAN20011	174.3033	534.6033	1.616869	0.002427	-
LVAN13515	1.613333	5.876667	1.864954	0.002438	Transposon Ty3-G Gag-Pol polyprotein [Trichinella nelsoni]
LVAN25149	8.95	4.16	-1.1053	0.00269	apoptosis-inducing factor [Litopenaeus vannamei]
LVAN02374	39.81	18.54667	-1.10197	0.002703	PREDICTED: sphingosine-1-phosphate lyase-like [Hyalella azteca]
MSTRG.29809	33.63667	13.07333	-1.36341	0.002773	cytochrome P450 2L1-like [Penaeus vannamei]
MSTRG.14534	78.20333	235.2167	1.588688	0.00313	zonadhesin-like [Penaeus vannamei]
MSTRG.2555	4.566667	18.06	1.983585	0.003251	general transcription factor 3C polypeptide 5-like [Penaeus vannamei]
LVAN23845	0.02	1.016667	5.667703	0.003253	heat shock protein 21 [Macrobrachium rosenbergii]
LVAN08498	28.06333	12.4	-1.17835	0.003297	-
LVAN01232	0.076667	1.79	4.545216	0.003437	PREDICTED: uncharacterized protein LOC108666259 [Hyalella azteca]
MSTRG.30846	1299.557	2643.13	1.024228	0.003661	Gamma-crystallin-related, partial [Trinorchestia longiramus]

LVAN15695	82.31	177.6533	1.109925	0.003822	PREDICTED: parafibromin-like [Hyalella azteca]
LVAN05326	8.656667	30.75	1.828703	0.003822	PREDICTED: probable E3 ubiquitin-protein ligase DTX2 [Eurypyga helias]
LVAN03710	31.22333	106.33	1.767852	0.003859	innexin 7 [Homarus americanus]
MSTRG.33540	3.343333	1.42	-1.2354	0.00394	sphingosine-1-phosphate phosphatase 2-like [Penaeus vannamei]
LVAN02946	0.483333	3.766667	2.962198	0.003963	antilipopolysaccharide factor isoform 5 [Fenneropenaeus chinensis]
LVAN05145	39.57333	19.35667	-1.0317	0.00402	PREDICTED: UDP-glucuronosyltransferase 2B19-like isoform X1 [Hyalella azteca]
MSTRG.29785	15.31	34.44667	1.16989	0.004075	zinc finger X-linked protein ZXDA-like isoform X1 [Penaeus vannamei]
LVAN25204	0.633333	4.546667	2.843772	0.004402	AGAP003729-PA [Anopheles gambiae str. PEST] [Anopheles gambiae]
LVAN12625	0.093333	2.873333	4.944189	0.004575	AGAP005712-PC [Anopheles gambiae str. PEST] [Anopheles gambiae]
LVAN10876	47.62667	22.66	-1.07162	0.004575	Beta,beta-carotene 9',10'-oxygenase [Daphnia magna]
LVAN15312	2.356667	8.313333	1.818679	0.004813	juvenile hormone esterase-like carboxylesterase 1 [Eriocheir sinensis]
MSTRG.23702	2.893333	10.56667	1.868716	0.004901	gag-pol fusion protein [Penaeus vannamei]
LVAN12525	60.97	134.8967	1.145683	0.00495	PREDICTED: zinc metalloproteinase nas-4-like [Pogonomyrmex barbatus]
MSTRG.41684	47.55667	12.35667	-1.94436	0.005052	uncharacterized protein LOC113800107 isoform X1 [Penaeus vannamei]
MSTRG.6378	4.6	12.08	1.392915	0.005073	-
LVAN14291	5.106667	1.22	-2.0655	0.005445	PREDICTED: pre-rRNA-processing protein TSR2 homolog isoform X2 [Lingula anatina]
MSTRG.37424	0.306667	9.013333	4.877317	0.005716	uncharacterized protein LOC113827644 [Penaeus vannamei]
MSTRG.16678	3.103333	0.273333	-3.50509	0.005722	polycomb group RING finger protein 3-like [Penaeus vannamei]
LVAN04989	1.226667	21.89667	4.157896	0.005847	PREDICTED: filaggrin-2-like isoform X3 [Hyalella azteca]
LVAN01206	0.056667	13.13333	7.856517	0.005978	prophenoloxidase activating enzyme 2 [Litopenaeus vannamei]
LVAN12300	21.01667	44.18667	1.072077	0.006392	Phosphoglucomutase-1 protein [Daphnia magna]
MSTRG.17657	59.86667	180.08	1.588813	0.006495	cilia- and flagella-associated protein 251-like isoform X1 [Penaeus vannamei]
LVAN24425	92.83333	37.85	-1.29435	0.006576	PREDICTED: hemocyte protein-glutamine gamma-glutamyltransferase-like [Hyalella azteca]
LVAN01421	0.18	2.226667	3.628817	0.006814	serine proteinase [Litopenaeus vannamei]
LVAN08170	0.026667	1.83	6.100662	0.006832	-

LVAN05380	23	8.73	-1.39758	0.006934	PREDICTED: epithelial chloride channel protein-like [Nanorana parkeri]
LVAN04834	80.5	180.8433	1.16768	0.007368	-
LVAN23018	0.643333	4.043333	2.651907	0.007582	PREDICTED: tyrosine decarboxylase-like [Limulus polyphemus]
LVAN08919	27.39333	70.33333	1.360384	0.00771	flotillin-2 [Litopenaeus vannamei]
MSTRG.38195	14.01333	4.79	-1.5487	0.00771	galactoside 3(4)-L-fucosyltransferase-like, partial [Penaeus vannamei]
LVAN20590	1.926667	0.166667	-3.53107	0.00771	PREDICTED: lactosylceramide 4-alpha-galactosyltransferase-like [Hyalella azteca]
LVAN22142	50.84333	24.41	-1.05859	0.00771	PREDICTED: cytochrome P450 9e2-like [Hyalella azteca]
LVAN18517	1.39	60.09333	5.434048	0.007727	chitinase-4 [Penaeus monodon]
LVAN14784	0.65	1.71	1.395485	0.007729	sodium leak channel non-selective [Cancer borealis]
LVAN05961	3.75	1.076667	-1.80032	0.007833	-
LVAN10801	13.10667	33.08667	1.335949	0.007859	trehalose-6-phosphate synthase [Fenneropenaeus chinensis]
LVAN11287	0.001	2.013333	10.97537	0.007898	PREDICTED: organic cation transporter protein-like [Hyalella azteca]
LVAN09257	79.83333	215.23	1.430816	0.007994	PREDICTED: hexokinase type 2-like isoform X2 [Hyalella azteca]
LVAN00802	3.383333	1.063333	-1.66985	0.008027	cytochrome P450 [Panulirus argus]
LVAN22546	356.1933	171.9333	-1.05081	0.008265	Kazal-type serine proteinase inhibitor 2 [Fenneropenaeus chinensis]
MSTRG.22672	1.003333	3.76	1.905932	0.008376	protein ALP1-like [Aphis craccivora]
LVAN12502	3.67	1.276667	-1.5234	0.00845	APOBEC1 complementation factor [Cricetulus griseus]
MSTRG.33552	10.86333	32.73667	1.591441	0.008759	glycine N-acyltransferase-like protein 3 isoform X2 [Pelodiscus sinensis]
LVAN13121	767.33	1751.913	1.191012	0.008904	PREDICTED: low-density lipoprotein receptor-related protein 2-like [Hyalella azteca]
LVAN15469	0.263333	3.756667	3.834491	0.008904	-
MSTRG.13219	2.313333	7.763333	1.746703	0.009414	PREDICTED: uncharacterized transmembrane protein DDB_G0289901-like [Hyalella azteca]
LVAN08495	12.43667	3.096667	-2.00581	0.009428	PREDICTED: biglycan [Poecilia reticulata]
LVAN16507	0.446667	2.613333	2.548621	0.009857	PREDICTED: platelet binding protein GspB-like [Xenopus tropicalis]
MSTRG.6632	0.066667	0.983333	3.882643	0.009962	PREDICTED: prosalusin [Nanorana parkeri]
MSTRG.20390	5.473333	13.34667	1.285988	0.009979	serine protease inhibitor I/II-like [Penaeus vannamei]

LVAN06562	6.68	19.11333	1.516659	0.010006	PREDICTED: ETS-related transcription factor Elf-4-like [Hyalella azteca]
MSTRG.35769	3.916667	8.63	1.139734	0.010051	exonuclease mut-7 homolog [Penaeus vannamei]
LVAN02957	3.06	0.673333	-2.18414	0.01007	PREDICTED: glycine receptor subunit alphaZ1-like [Parasteatoda tepidariorum]
MSTRG.20212	37.17667	82.37333	1.14778	0.010114	-
LVAN17699	1.43	0.17	-3.07241	0.010176	PREDICTED: arf-GAP with SH3 domain, ANK repeat and PH domain-containing protein 1 isoform X4 [Cerapachys biroi]
MSTRG.12985	5.386667	15.76333	1.549108	0.010213	RecName: Full=Penaeidin-4a; Short=Pen-4a; Flags: Precursor [Penaeus vannamei]
LVAN03889	23.59	49.81667	1.078453	0.010233	Riboflavin kinase [Zootermopsis nevadensis]
LVAN14906	2.993333	9.71	1.697718	0.010316	3-oxoacyl-[acyl-carrier-protein] reductase [Zootermopsis nevadensis]
LVAN12494	221.2867	576.4933	1.381387	0.010435	PREDICTED: sulfoquinovosidase-like [Hyalella azteca]
LVAN19796	4.503333	2	-1.17099	0.01044	-
MSTRG.32534	0.483333	2.79	2.529175	0.010525	uncharacterized protein LOC113823796 [Penaeus vannamei]
LVAN20504	2.076667	5.153333	1.311236	0.010555	PREDICTED: sodium/glucose cotransporter 5-like [Limulus polyphemus]
LVAN07131	20.09	41.18667	1.0357	0.010818	Phospholipid scramblase 2 [Zootermopsis nevadensis]
LVAN11936	3.543333	14.46	2.028888	0.011052	crustacean hematopoietic factor-like protein [Litopenaeus vannamei]
MSTRG.35205	0.99	5.236667	2.403148	0.01107	amylase [Penaeus vannamei]
LVAN15054	0.456667	14.30667	4.969402	0.01107	caspase [Eriocheir sinensis]
LVAN10953	0.083333	0.756667	3.182692	0.011117	PREDICTED: protein Wnt-8b-like [Limulus polyphemus]
MSTRG.29357	9.256667	2.01	-2.2033	0.011525	-
LVAN03709	9.326667	27.28	1.54841	0.011525	innexin 7 [Homarus americanus]
MSTRG.43424	3.833333	14.71667	1.94078	0.011812	small cysteine and glycine repeat-containing protein 2-like [Penaeus vannamei]
MSTRG.20181	5.116667	2.543333	-1.00848	0.011812	-
MSTRG.37533	4.326667	9.583333	1.147272	0.01272	sodium/glucose cotransporter 5-like [Penaeus vannamei]
LVAN15530	21.97	50.24	1.193302	0.012969	-
MSTRG.24253	4.476667	0.77	-2.53949	0.013032	Transposon Ty3-G Gag-Pol polyprotein [Stylophora pistillata]
LVAN08430	2.94	0.416667	-2.81885	0.013068	vitellogenin, partial [Penaeus monodon]

LVAN13242	11.36333	3.426667	-1.72951	0.01348	-
LVAN05758	8.98	25.02667	1.478679	0.013785	C-type lectin [Procambarus clarkii]
LVAN08157	4.306667	10.85	1.333051	0.013785	alpha-amylase, partial [Litopenaeus vannamei]
MSTRG.20057	11.18333	34.61333	1.629978	0.013832	hypothetical protein C7M84_011991 [Penaeus vannamei]
MSTRG.19128	14.49333	39.59	1.449747	0.013937	insulin-like growth factor-binding protein-related protein 1 [Penaeus vannamei]
LVAN17703	0.146667	2.416667	4.042406	0.014186	triosephosphate isomerase [Penaeus monodon]
LVAN08648	0.02	4.013333	7.648657	0.014254	caspase [Eriocheir sinensis]
LVAN03640	6.033333	17.27	1.517241	0.014254	-
LVAN06601	22.02333	8.32	-1.40438	0.014596	PREDICTED: asporin-like [Sinocyclocheilus rhinocerous]
MSTRG.32308	1062.24	161.0467	-2.72156	0.015197	hemocyanin C chain-like [Penaeus vannamei]
LVAN21999	10.12333	3.126667	-1.69499	0.015236	chitinase 5 [Litopenaeus vannamei]
MSTRG.23680	3.283333	9.44	1.523626	0.015273	hypothetical protein C7M84_003490 [Penaeus vannamei]
MSTRG.29435	4.366667	10.88667	1.317958	0.01538	mannose-1-phosphate guanyltransferase beta-like [Penaeus vannamei]
LVAN15367	13.51333	30.46333	1.17269	0.016138	Dystroglycan, partial [Zootermopsis nevadensis]
LVAN12704	0.886667	5.283333	2.574985	0.016138	ecdysteroid regulated-like protein [Litopenaeus vannamei]
LVAN20413	1.976667	8.13	2.040186	0.01616	X-linked interleukin-1 receptor accessory protein-like 1 [Zootermopsis nevadensis]
LVAN07290	1.276667	0.146667	-3.12177	0.01658	PREDICTED: uncharacterized protein LOC100745028 [Bombus impatiens]
MSTRG.43422	11.30333	32.22667	1.511507	0.016582	small cysteine and glycine repeat-containing protein 2-like [Penaeus vannamei]
MSTRG.31773	7.553333	1.336667	-2.49847	0.016619	dnaJ homolog subfamily C member 9-like [Penaeus vannamei]
LVAN22775	350.9333	147.0033	-1.25535	0.016903	hemocyanin subunit L2, partial [Litopenaeus vannamei]
LVAN17888	4.276667	11.13333	1.380327	0.016903	Nostrin [Zootermopsis nevadensis]
LVAN20376	0.486667	2.63	2.434057	0.01725	PREDICTED: NAD(P) transhydrogenase, mitochondrial-like [Aplysia californica]
LVAN25367	0.866667	3.563333	2.039678	0.017311	PREDICTED: alpha-(1,3)-fucosyltransferase C-like [Hyalella azteca]
MSTRG.35616	3.316667	0.486667	-2.76873	0.017464	Nose resistant to fluoxetine protein 6 [Penaeus vannamei]
LVAN22450	1.216667	0.143333	-3.08549	0.017592	PREDICTED: alpha-(1,6)-fucosyltransferase-like [Parasteatoda tepidariorum]
LVAN24190	1.026667	6.93	2.754888	0.017771	PREDICTED: gamma-butyrobetaine dioxygenase-like isoform X2 [Lingula anatina]

MSTRG.5662	20.73333	8.453333	-1.29436	0.01784	-
LVAN06850	1.476667	4.403333	1.576252	0.018206	-
MSTRG.21080	8.723333	19.51667	1.161755	0.018269	-
LVAN24554	8.953333	4.153333	-1.10816	0.018759	PREDICTED: neprilysin-like isoform X3 [Hyalella azteca]
LVAN22696	2.933333	0.973333	-1.59154	0.018788	PREDICTED: metalloreductase STEAP4-like [Hyalella azteca]
MSTRG.21114	0.42	1.373333	1.709221	0.019035	-
MSTRG.22944	0.026667	1.423333	5.738092	0.019091	endocuticle structural glycoprotein SgAbd-8-like [Penaeus vannamei]
LVAN22766	233.47	108.2	-1.10954	0.019181	hemocyanin [Fenneropenaeus chinensis]
MSTRG.13795	6.94	0.563333	-3.62287	0.019261	platelet glycoprotein Ib alpha chain-like isoform X2 [Penaeus vannamei]
LVAN12495	79.45	219.75	1.467744	0.019471	PREDICTED: sulfoquinovosidase-like [Hyalella azteca]
MSTRG.23575	7.913333	1.98	-1.99879	0.019986	integrin alpha 8 [Penaeus chinensis]
MSTRG.19615	0.553333	4.21	2.927599	0.020094	-
LVAN04343	19.59333	40.87333	1.060797	0.02039	PREDICTED: hsp70-binding protein 1-like isoform X1 [Limulus polyphemus]
MSTRG.28023	32.17667	11.95	-1.429	0.020458	Gamma-crystallin-1, partial [Orchesella cincta]
LVAN20981	14.82667	41.97	1.501164	0.02104	PREDICTED: eukaryotic translation initiation factor 2 subunit 3 [Corvus brachyrhynchos]
MSTRG.41129	0.296667	2.973333	3.325166	0.021359	hypothetical protein C7M84_023505 [Penaeus vannamei]
LVAN10543	6.476667	19.92333	1.621136	0.021629	-
LVAN14267	0.426667	3.063333	2.843921	0.021975	hypothetical protein, partial [Scylla paramamosain]
LVAN09962	1.123333	5.433333	2.274051	0.022322	PREDICTED: glucosylceramidase-like isoform X1 [Hyalella azteca]
MSTRG.17808	0.093333	3.5	5.228819	0.022515	-
LVAN16434	41.33667	88.73667	1.102108	0.022516	-
LVAN19593	2.236667	6.576667	1.556006	0.023022	-
MSTRG.11079	13.70667	29.06667	1.084488	0.023175	NADH-cytochrome b5 reductase-like [Penaeus vannamei]
LVAN13712	3.88	13.01333	1.745862	0.023373	lipoprotein receptor 2A [Pandalopsis japonica]
MSTRG.21847	5.116667	24.13333	2.237751	0.023531	secretory leukocyte proteinase inhibitor [Penaeus vannamei]
LVAN09843	49.90667	137.9567	1.466911	0.023693	PREDICTED: uncharacterized protein LOC108675418 [Hyalella azteca]

LVAN19598	2.25	6.226667	1.468535	0.023932	PREDICTED: microtubule-associated protein futsch-like [Hyalella azteca]
LVAN17949	1.15	5.85	2.346803	0.023967	PREDICTED: serine/threonine-protein kinase SBK1-like [Hyalella azteca]
LVAN02831	0.13	0.896667	2.78606	0.024054	juvenile hormone esterase-like carboxylesterase 1 [Eriocheir sinensis]
LVAN11581	0.006667	0.31	5.539159	0.024133	hypothetical protein CAOG_00428 [Capsaspora owczarzaki ATCC 30864]
LVAN24042	0.153333	1.376667	3.166436	0.024908	Low choriolytic enzyme [Daphnia magna]
MSTRG.28080	17.92	8.793333	-1.02709	0.02554	hypothetical protein Anas_03030 [Armadillidium nasatum]
LVAN19365	1.15	0.33	-1.8011	0.025917	PREDICTED: tetratricopeptide repeat protein 21B-like [Hyalella azteca]
LVAN11202	0.883333	3.53	1.998638	0.026017	PREDICTED: inositol hexakisphosphate and diphosphoinositol-pentakisphosphate kinase-like [Limulus polyphemus]
LVAN00201	15.14667	113.91	2.910822	0.026017	PDGF/VEGF-related factor 1 [Eriocheir sinensis]
MSTRG.11151	28.93667	4.223333	-2.77644	0.026353	phosphoglucomutase [Penaeus vannamei]
LVAN03764	5.526667	13.16333	1.252043	0.026549	PREDICTED: low-density lipoprotein receptor-related protein-like [Parasteatoda tepidariorum]
LVAN14506	7.96	2.183333	-1.86624	0.026602	PREDICTED: galactose-specific lectin nattectin-like [Hyalella azteca]
LVAN21214	14.32333	6.55	-1.1288	0.02683	RecName: Full=Crustacyanin-A2 subunit
MSTRG.30546	0.043333	1.616667	5.221401	0.027667	-
LVAN16195	12.35	25.75	1.060061	0.027882	PREDICTED: glycerol-3-phosphate dehydrogenase, mitochondrial-like [Hyalella azteca]
MSTRG.36995	11.12333	26.54333	1.25476	0.028662	cyclin-dependent kinase inhibitor 1B-like [Penaeus vannamei]
LVAN11291	38.12	18.67333	-1.02957	0.028662	PREDICTED: organic cation transporter protein-like [Hyalella azteca]
MSTRG.26816	3.79	0.836667	-2.17947	0.028804	RNA polymerase [Penaeus vannamei]
LVAN08220	0.106667	1.35	3.661778	0.029142	PREDICTED: deoxynucleoside kinase-like isoform X2 [Hyalella azteca]
MSTRG.41872	4.456667	10.43	1.226702	0.029189	aurora kinase A-like [Penaeus vannamei]
MSTRG.32905	11.82667	26.68	1.173715	0.029801	DNA-(apurinic or apyrimidinic site) lyase 2-like, partial [Penaeus vannamei]
MSTRG.41426	0.976667	5.216667	2.41719	0.030171	uncharacterized protein LOC113799928 [Penaeus vannamei]
LVAN03284	62.56	168.1333	1.426293	0.030171	anti-lipopolysaccharide AV-R isoform [Litopenaeus vannamei]
LVAN04988	0.176667	1.536667	3.120702	0.030171	PREDICTED: fibrillin-1-like [Limulus polyphemus]

LVAN12822	9.226667	3.45	-1.41921	0.030328	PREDICTED: UDP-glucuronosyltransferase-like isoform X4 [Hyalella azteca]
LVAN12044	3.963333	1.496667	-1.40496	0.030651	Carboxypeptidase E, partial [Anoplophora glabripennis]
LVAN02714	42.47333	86.77	1.030639	0.031096	integrin [Litopenaeus vannamei]
LVAN16093	124.6767	25.07667	-2.31377	0.03139	chitin binding-like protein [Fenneropenaeus chinensis]
MSTRG.37181	0.483333	0.153333	-1.65635	0.031496	neuronal acetylcholine receptor subunit alpha-7-like [Penaeus vannamei]
LVAN03978	7.92	16.94	1.096862	0.031576	Tyrosine-protein phosphatase 69D, partial [Trachymyrmex zeteki]
LVAN07756	5.043333	1.82	-1.47044	0.031955	PREDICTED: membrane-bound alkaline phosphatase-like, partial [Hyalella azteca]
MSTRG.35341	1.963333	0.386667	-2.34414	0.032356	-
LVAN23848	0.053333	0.953333	4.159871	0.032434	heat shock protein 21 [Macrobrachium rosenbergii]
LVAN15388	2.96	9.926667	1.745712	0.033279	PREDICTED: uncharacterized protein LOC108679670 [Hyalella azteca]
MSTRG.36155	1.993333	6.326667	1.666263	0.033304	mucin-5AC-like [Penaeus vannamei]
LVAN05590	7.13	14.38667	1.012758	0.034561	PREDICTED: uncharacterized protein LOC106460216 [Limulus polyphemus]
LVAN08975	4.13	1.383333	-1.57799	0.034582	PREDICTED: microfibril-associated glycoprotein 4-like [Clupea harengus]
LVAN08407	4.736667	2.176667	-1.12175	0.034909	AAEL012091-PA [Aedes aegypti]
LVAN21713	26.37	53.63333	1.024232	0.034975	PREDICTED: bifunctional glutamate/proline--tRNA ligase-like [Hyalella azteca]
LVAN12296	2.863333	1.38	-1.05303	0.035167	Apolipophorins [Daphnia magna]
LVAN03107	1.64	0.183333	-3.16115	0.035167	PREDICTED: dysbindin-like isoform X1 [Hyalella azteca]
MSTRG.19804	2.856667	1.146667	-1.31689	0.035167	hypothetical protein C7M84_025354 [Penaeus vannamei]
MSTRG.30547	1.17	8.87	2.922426	0.035224	-
LVAN04047	1.213333	0.243333	-2.31797	0.035224	I-connectin [Procambarus clarkii]
LVAN05544	2.3	8.35	1.860142	0.036178	PREDICTED: uncharacterized protein LOC108670255 [Hyalella azteca]
LVAN04844	31.57	76.22	1.271615	0.036602	PREDICTED: metallophosphoesterase domain-containing protein 1-like [Limulus polyphemus]
LVAN05134	1.82	18.38	3.336126	0.036619	triacylglycerol lipase [Portunus trituberculatus]
LVAN09040	0.053333	1.223333	4.519636	0.03733	PREDICTED: flocculation protein FLO11-like, partial [Hyalella azteca]
LVAN01378	3.363333	10.72333	1.67279	0.039302	estrogen sulfotransferase [Scylla olivacea]

LVAN12401	5.753333	12.47333	1.116377	0.042062	PREDICTED: lymphocyte-specific helicase-like isoform X1 [Lingula anatina]
LVAN21088	1.9	5.25	1.466318	0.042082	PREDICTED: poly [ADP-ribose] polymerase 3-like [Hyalella azteca]
LVAN20302	1.786667	0.44	-2.0217	0.044789	PREDICTED: nose resistant to fluoxetine protein 6-like [Crassostrea gigas]
MSTRG.25077	18.87	7.203333	-1.38936	0.045231	-
LVAN02098	13.38	31.35	1.228387	0.046664	PREDICTED: phosphatidylinositol 3,4,5-trisphosphate 3-phosphatase and dual-specificity protein phosphatase PTEN isoform X1 [Halyomorpha halys]
MSTRG.13068	4.28	9.426667	1.139137	0.046786	uncharacterized protein LOC113809054 [Penaeus vannamei]
MSTRG.23707	3.666667	8.69	1.244887	0.047741	zinc knuckle protein [Penaeus vannamei]
LVAN17717	8.96	20.34667	1.183222	0.047892	PREDICTED: T-complex protein 11-like protein 1 isoform X2 [Athalia rosae]
LVAN07490	11.89333	5.803333	-1.0352	0.047892	PREDICTED: ethanolaminephosphotransferase 1-like [Hyalella azteca]
LVAN23214	22.7	61.88	1.446781	0.048201	serine protease 1 [Litopenaeus vannamei]
LVAN24031	0.446667	3.363333	2.912621	0.048383	prophenoloxidase activating factor 1 [Scylla paramamosain]
LVAN21500	3.693333	1.243333	-1.57071	0.048672	PREDICTED: UDP-glucuronosyltransferase 2C1-like [Hyalella azteca]
LVAN23846	0.053333	0.733333	3.78136	0.048894	heat shock protein 21 [Macrobrachium rosenbergii]
MSTRG.41812	0.04	1.613333	5.333901	0.048894	uncharacterized protein LOC113809676 [Penaeus vannamei]
LVAN06942	4.243333	10.02	1.239613	0.049106	hypothetical protein g.11536 [Clastoptera arizonana]
LVAN01075	10.91333	23.36667	1.09836	0.049639	PREDICTED: triple functional domain protein-like [Hyalella azteca]
MSTRG.26817	2.696667	0.45	-2.58318	0.049723	hypothetical protein C7M84_002796 [Penaeus vannamei]
LVAN22749	5.553333	11.95667	1.106389	0.049879	PREDICTED: LOW QUALITY PROTEIN: extended synaptotagmin-1-like [Hyalella azteca]

Metabolite_ID	Modes	S20507-12h	R20523-12h	Log2(fc)	VIP	Description
M173T281_2	Negative ionization	595950.0243	338696.3	-0.8152	2.997618	Gly-Val
M217T252	Negative ionization	774839.2603	440758.4	-0.81391	3.559047	Ser-Leu
M351T39	Negative ionization	35756.12067	193514.8	2.436182	2.857698	Prostaglandin i2
M457T231	Negative ionization	11392.23071	39849.6	1.806515	1.183441	1-(5z,8z,11z,14z-eicosatetraenoyl)-sn-glycero-3-phosphate

M251T58	Negative ionization	9247.902229	33860.23	1.872394	1.105783	N-2-hydroxyethylpiperazine-n-3-propanesulfonic acid
M216T344	Negative ionization	144235.1347	94312.26	-0.61291	1.257724	Ala-Gln
M487T154	Negative ionization	586916.8368	301340.8	-0.96176	3.624134	Oxyresveratrol
M203T412	Negative ionization	595193.682	426705.6	-0.48012	2.245442	Ala-asp
M429T391	Negative ionization	59152.33249	24701.38	-1.25984	1.072477	Ketoprofen .beta.-d-glucuronide
M568T33	Negative ionization	160292.7664	71989.43	-1.15485	1.807228	1-stearoyl-2-hydroxy-sn-glycero-3-phosphocholine
M300T435	Negative ionization	63709.14261	101052.1	0.665527	1.445349	N-Acetylglucosamine 1-phosphate
M535T208	Negative ionization	284687.8339	162950.1	-0.80495	2.304123	3-deoxy-d-glycero-d-galacto-2-nonulosonic acid
M259T354	Negative ionization	1363323.15	881083.9	-0.62978	4.135585	Glu-Leu
M229T192	Negative ionization	2210865.395	1611104	-0.45656	3.79325	Ile-Val
M145T330_2	Negative ionization	304700.6378	219016.7	-0.47635	1.576185	Gly-Ala
M243T155	Negative ionization	4127695.448	3128241	-0.39998	5.506641	Uridine
M544T34	Negative ionization	104588.3401	44953.68	-1.21821	1.453248	1-o-hexadecyl-2-o-ethyl-sn-glycero-3-phosphocholine
M243T181	Negative ionization	355042.4905	252620.2	-0.49102	1.537782	Ile-Leu
M187T278	Negative ionization	2753901.77	2003104	-0.45924	3.83857	Gly-Leu
M327T131	Negative ionization	167353.1166	53851.05	-1.63585	1.979468	4'-hydroxy-2'-methyl-3,4,5-trimethoxychalcone
M266T104	Negative ionization	100199.0873	239571.7	1.257588	2.56623	Phenol, 4-[[4-(4-morpholinyl)butyl]thio]-
M386T38	Negative ionization	28101.17423	69421.14	1.304745	1.569882	Urapidil
M300T143	Negative ionization	2037594.194	825155.4	-1.30413	6.496261	N-acetyl-d-glucosamine 6-phosphate
M180T142	Negative ionization	207339.6076	613168.8	1.564288	3.897875	Acamprosate
M149T328_2	Negative ionization	520287.3849	408315.6	-0.34962	1.333706	D-lyxose
M129T387_2	Negative ionization	494521.3941	304897.7	-0.69771	1.835584	Citraconic acid
M367T65	Negative ionization	48715.12548	111923.9	1.200076	1.576795	11-dehydrothromboxane b2
M187T249_2	Negative ionization	370836.6896	287725.9	-0.36609	1.091267	Val-Ala
M147T387	Negative ionization	612814.5697	363646.4	-0.75291	2.149757	(S)-2-Hydroxyglutarate
M132T395_2	Negative ionization	1288694.347	1117300	-0.20589	1.208756	D-aspartic acid

M172T188	Negative ionization	16523.96028	45296.72	1.454847	1.232044	Acetyl-dl-leucine
M165T114	Negative ionization	18729.75337	55096.68	1.556633	1.227182	1-methylxanthine
M322T132	Negative ionization	416530.4306	245220.3	-0.76434	2.210292	Anorexigenic peptide
M219T312	Negative ionization	466623.547	341664	-0.44968	1.647919	Thr-Thr
M606T424	Negative ionization	32986.14795	80898.36	1.29425	1.589442	Udp-n-acetylglucosamine
M147T247_2	Negative ionization	484910.7655	454384.9	-0.0938	1.029135	trans-cinnamate
M324T127	Negative ionization	1433889.791	883935.7	-0.69792	4.061953	Cycloxydime
M261T392	Positive ionization	6506320.915	1855364	-1.81014	11.38958	Gly-Gly-Lys
M261T256	Positive ionization	23080.33706	66728.1	1.53163	1.208679	Ropinirole
M147T333	Positive ionization	308913.5202	216147.4	-0.51519	1.592519	Ala-Gly
M323T399	Positive ionization	375534.2165	57934.84	-2.69644	2.910384	Matairesinol
M247T370	Positive ionization	2990275.869	1724026	-0.7945	6.215072	Val-Glu
M140T107	Positive ionization	69465.95847	29405.97	-1.2402	1.050453	N-carboxyethyl-.gamma.-aminobutyric acid
M538T391	Positive ionization	1364203.405	486700	-1.48695	4.804121	4-demethylsimmondsin 2'-ferulate
M243T352	Positive ionization	416902.4288	249796.9	-0.73895	2.10223	Ser-His
M298T420	Positive ionization	205211.9866	143310.9	-0.51797	1.205386	L-cysteine-glutathione disulfide
M205T287	Positive ionization	718875.1699	439680.5	-0.70929	2.719812	Val-Ser
M207T287	Positive ionization	270138.4449	193151.8	-0.48396	1.357992	Gly-Met
M355T532	Positive ionization	112367.8	64640.02	-0.79773	1.080162	Deoxycorticosterone acetate
M645T259	Positive ionization	128461.9788	58960.78	-1.12351	1.414569	8-iso-prostaglandin a2-biotin
M262T411	Positive ionization	1018063.513	720755.6	-0.49825	2.609108	Glu-Asn
M219T256	Positive ionization	2922653.084	1937296	-0.59323	4.889724	Val-Thr
M175T282	Positive ionization	492086.147	288470.4	-0.77049	2.163246	Val-Gly
M279T335	Positive ionization	73345.94782	20850.09	-1.81466	1.181918	Dibutyl phthalate
M516T290	Positive ionization	138206.4691	72869.91	-0.92343	1.34678	Enzastaurin
M258T170	Positive ionization	1185465.689	558267.1	-1.08643	4.154231	2'-o-methylcytidine

M154T255	Positive ionization	28736.98667	68085.98	1.244449	1.118227	Dopamine
M353T481	Positive ionization	187175.7001	94751.01	-0.98218	1.55146	Ajmalicine
M220T361	Positive ionization	164370.6008	110502	-0.57288	1.11631	Ser-Asn
M122T166	Positive ionization	56597.46585	10098.62	-2.48658	1.134512	Phenylethylamine
M170T337_2	Positive ionization	572787.7159	1478137	1.367707	5.133695	Diphenylamine
M229T298	Positive ionization	9488180.437	5521769	-0.781	10.03474	Pro-leu
M219T468	Positive ionization	203514.494	75400.22	-1.43249	1.694538	2-phenylpiperidine-2-acetamide
M482T189	Positive ionization	1159690.745	771514.8	-0.58797	2.968593	2-hexadecanoylthio-1-ethylphosphorylcholine
M260T284	Positive ionization	2307422.617	1567408	-0.5579	3.844301	Leu-Gln
M110T369_2	Positive ionization	7798978.85	6107323	-0.35275	5.759837	N-.alpha.-(tert-butoxycarbonyl)-l-histidine
M262T345_2	Positive ionization	177581.2334	122370.6	-0.53722	1.120059	Tolfenamic acid
M231T371_2	Positive ionization	2221764.875	28327951	3.672448	27.87679	Lys-Trp-Lys
M126T223	Positive ionization	134650.5106	61639.04	-1.1273	1.407751	5-methyl-2'-deoxycytidine
M327T352	Positive ionization	423195.0284	322861.5	-0.39041	1.311513	Bilobalide
M152T258_2	Positive ionization	15032606.95	10003896	-0.58753	11.95445	Guanine
M156T369_2	Positive ionization	11011392.36	8783724	-0.32609	6.338082	Histidine
M284T258	Positive ionization	10607012.57	6888556	-0.62274	10.28762	His-Lys
M133T159	Positive ionization	171356.0076	125214.5	-0.4526	1.093918	D-arabinose
M120T121	Positive ionization	117922.2359	65994.24	-0.83742	1.095147	L-.beta.-homoserine
M143T281_2	Positive ionization	878774.7888	651366.4	-0.43202	2.378098	Glycyl-l-norleucine
M265T353	Positive ionization	175828.6634	127310.3	-0.46582	1.058994	Thiamine
M459T233	Positive ionization	36003.3147	95006.6	1.399898	1.323527	Fumagillin
M187T330	Positive ionization	736216.8484	455894.2	-0.69143	2.48476	Pro-Ala
M277T441	Positive ionization	464234.7023	84485.36	-2.45808	3.063737	L-saccharopine
M325T431	Positive ionization	90948.64594	141754.7	0.640273	1.283797	Uridine 5'-monophosphate
M309T496	Positive ionization	2885044.338	1579904	-0.86876	5.160007	Fructoselysine

M246T300	Positive ionization	2098302.042	1398683	-0.58515	4.103321	Val-Gln
M276T200	Positive ionization	21894.38244	97902.14	2.16078	1.481746	Flumazenil
M258T223	Positive ionization	107656.3089	50588.35	-1.08956	1.211436	5-methylcytidine
M254T153	Positive ionization	279480.0478	154974.2	-0.85072	1.611746	Normorphine
M100T62	Positive ionization	354927.0767	1014580	1.515287	4.544926	2-piperidone
M284T151	Positive ionization	224126.0025	1138163	2.344326	5.11356	Imazapyr
M207T330	Positive ionization	767099.307	547644.5	-0.48617	2.340572	Ser-Thr
M261T356	Positive ionization	1560169.26	1072045	-0.54134	3.584608	Ile-Glu
M201T81_2	Positive ionization	2166533.318	1250653	-0.79271	4.31148	Pro-Glu-Arg
M319T447	Positive ionization	498027.4995	301387.3	-0.72461	1.987296	Asp-Gly-Lys
M511T166	Positive ionization	162402.7883	95473.31	-0.76641	1.255886	Leukotriene d4 methyl ester
M217T409	Positive ionization	686372.7045	535915.1	-0.35699	1.720893	Glu-Glu-Arg
M318T181	Positive ionization	131332.9695	167583.4	0.35165	1.135694	Trp-Ile
M519T158_1	Positive ionization	184814.7185	108734.2	-0.76527	1.429666	D-glucosamine 1-phosphate
M277T37	Positive ionization	285247.1171	147494.3	-0.95155	1.839074	[6]-shogaol
M419T297	Positive ionization	501634.0841	364320.2	-0.46143	1.669884	Leu-Met-Arg
M328T510	Positive ionization	123036.2188	70179.68	-0.80996	1.106392	Piperidine, 4-(5h-dibenzo[a,d]cyclohepten-5-ylidene)-1-[4-(2h-tetrazol-5-yl)butyl]-
M279T361	Positive ionization	453931.0122	353147.6	-0.3622	1.279546	gamma-Glutamyl-L-methionine
M286T162	Positive ionization	148385.6464	81414.86	-0.86599	1.325397	N-acetylcytidine
M113T159_2	Positive ionization	6787434.476	4950155	-0.45539	6.794522	Uracil
M135T258	Positive ionization	366549.5179	259881.7	-0.49615	1.687264	2-dimethylamino-6-hydroxypurine
M446T367	Positive ionization	149325.5468	84786.2	-0.81656	1.154211	Ile-Trp-Lys
M298T195	Positive ionization	285788.3218	187600.4	-0.60728	1.5611	2-o-methylguanosine
M341T36	Positive ionization	172955.7367	372680.3	1.107536	2.602615	4,5-epoxy-7z,10z,13z,16z,19z-docosapentaenoic acid, methyl ester

M191T70	Positive ionization	762531.2289	460302	-0.72822	2.420073	Thr-Ala
M285T406	Positive ionization	2170435.324	1816084	-0.25715	2.345676	His-Glu
M213T369	Positive ionization	1009753.622	830642.6	-0.2817	1.621914	Gly-His
M749T150	Positive ionization	331329.0705	630209	0.927566	3.014627	Phosphatidylethanolamine
M283T146	Positive ionization	293233.6916	164443.7	-0.83446	1.722926	2'-O-methylinosine
M177T342	Positive ionization	105502.5755	21219.09	-2.31384	1.412541	Gly-Thr
M340T372	Positive ionization	112111.5121	51675.65	-1.11738	1.179591	Methylergonovine
M159T120	Positive ionization	163178.6746	80113.83	-1.02633	1.335333	Alitame
M372T388	Positive ionization	47740.92578	151386.4	1.664937	1.727186	Tamoxifen
M317T415	Positive ionization	1882252.972	1464733	-0.36182	2.69121	Ala-Ala-Arg
M147T300	Positive ionization	159490.7185	113130.9	-0.49548	1.006973	.alpha.-pyrrolidinobutiophenone
M234T354	Positive ionization	264953.4606	190780.9	-0.47382	1.266477	Ser-Gln
M333T397_1	Positive ionization	86711.28535	222733.2	1.361025	2.064804	Estrone sulfate
M247T423	Positive ionization	232756.704	115621.2	-1.00942	1.61714	2-cis-4-trans-abscisic acid
M393T328	Positive ionization	110435.0851	64815.63	-0.76879	1.072526	Ala-Phe-Arg
M375T366	Positive ionization	540327.5876	419748.9	-0.36431	1.393302	Thr-Val-Arg
M305T38	Positive ionization	50931.5639	98165.53	0.946656	1.263944	Arachidonic acid (peroxide free)
M290T277	Positive ionization	279627.5757	218895.2	-0.35327	1.111274	Tetrahydropiperine
M209T428	Positive ionization	103232.2453	41599.59	-1.31125	1.065275	Dl-lanthionine
M317T366	Positive ionization	192788.9341	122572.7	-0.65338	1.09218	Leu-Gly-Lys
M217T211	Positive ionization	1527417.682	1080183	-0.49982	2.767502	Val-Val
M170T363	Positive ionization	125057.196	213609.6	0.772389	1.691434	1-methyl-l-histidine
M356T407	Positive ionization	207201.0389	102940.9	-1.00922	1.326918	Rotundine
M233T245	Positive ionization	2291057.513	1564124	-0.55066	3.596955	Ile-Thr
M166T225_2	Positive ionization	66970.35721	152665.9	1.188783	1.523769	7-methylguanine
M253T176	Positive ionization	1260677.127	1571355	0.31781	3.571903	2'-deoxyinosine

M253T69_1	Positive ionization	907700.9408	551759.1	-0.71818	2.772736	Nebularine
M496T219	Positive ionization	198258.6814	124124.7	-0.67559	1.085191	1-palmitoyl-sn-glycero-3-phosphocholine
M364T433_1	Positive ionization	72045.38119	118538.8	0.718382	1.16167	Guanosine 5'-monophosphate
M186T159	Positive ionization	554992.0585	357568.1	-0.63425	2.21223	N-acetyl-d-galactosamine
M279T261	Positive ionization	1392080.668	3568867	1.358223	7.659979	3-amino-1-propanesulfonic acid
M269T365	Positive ionization	366712.8868	263872.2	-0.47481	1.33795	Benzoic acid eugenyl ester
M164T85	Positive ionization	373013.9033	253265.5	-0.55858	1.639929	Etilefrine
M303T427	Positive ionization	743074.5302	615017	-0.27288	1.182683	Arg-Gln
M274T66	Positive ionization	6721887.612	8033958	0.257244	7.689598	Fenpropidin
M217T46	Positive ionization	678239.4611	484287.6	-0.48593	1.807678	N,n-diethyltryptamine
M177T153	Positive ionization	41719.80852	126864.3	1.604482	1.564729	3,4-methylenedioxybenzyl methyl ketoximine
M204T364	Positive ionization	424279.8815	338573.6	-0.32555	1.343989	N,n'-diacetylchitobiose
M425T388	Positive ionization	97586.0999	157818.1	0.693515	1.356853	His-Leu-Arg
M248T337	Positive ionization	522915.4508	407373.2	-0.36023	1.351362	Thr-Gln
M189T330_2	Positive ionization	357026.6577	264843.4	-0.43089	1.451541	Thr-Thr-Arg
M399T463	Positive ionization	526692.9585	362038.6	-0.54082	1.799502	S-adenosylmethionine
M165T49	Positive ionization	633578.6846	324532.8	-0.96516	2.445759	Cis-jasmone
M146T330_2	Positive ionization	1574394.533	2528578	0.683529	5.100343	N-methyl-l-leucine
M175T503	Positive ionization	64131764.97	59804444	-0.10079	5.75842	DL-arginine
M468T191	Positive ionization	1776574.938	1113696	-0.67374	3.994822	1-myristoyl-sn-glycero-3-phosphocholine
M375T157	Positive ionization	249160.4016	175338.7	-0.50693	1.183063	N-succinyl-5-aminoimidazole-4-carboxamide ribose
M221T412	Positive ionization	613673.0441	509239.1	-0.26913	1.304548	Ser-Asp
M345T367	Positive ionization	611912.1178	389268.5	-0.65256	2.198	Gly-Ile-Arg
M233T377	Positive ionization	1425807.225	993219	-0.5216	3.366579	Val-Asp
M170T148	Positive ionization	605897.7941	393677.2	-0.62206	2.259836	Furmecyclox
M146T43	Positive ionization	1017758.936	772516.8	-0.39776	1.847615	Dl-.beta.-homoleucine

M291T213	Positive ionization	6600586.442	4846568	-0.44563	5.184084	Inosine
M144T100	Positive ionization	1002965.938	723429	-0.47135	2.47391	N-ethylmaleimide
M789T46	Positive ionization	133502.0827	170123.3	0.349718	1.222558	1,2-dioleoyl-sn-glycero-3-phospho-l-serine
M511T445	Positive ionization	184462.9052	97564.16	-0.91891	1.261888	Asiatic acid
M430T72_3	Positive ionization	77995.32734	121590.8	0.640575	1.081629	N-oleoyl-phenylalanine
M312T437	Positive ionization	155362.9796	185255.1	0.253871	1.091931	His-Arg
M138T217_3	Positive ionization	6654795.418	12484728	0.907698	13.31018	Methylpicolinate
M203T230_2	Positive ionization	693504.8552	499687.4	-0.47288	1.645894	Ile-Ala
M276T410	Positive ionization	2759173.981	1844600	-0.58093	4.105427	Glu-Gln
M191T421	Positive ionization	191303.5181	145563.9	-0.39421	1.017906	Gly-Asp
M230T324	Positive ionization	88238.99211	26269.81	-1.74801	1.055406	Ergothioneine
M94T217	Positive ionization	396120.3116	748046.6	0.91719	3.118151	4-picoline
M215T284	Positive ionization	2135757.404	1359369	-0.65181	3.803205	Pro-val
M317T36	Positive ionization	289359.0978	604126.2	1.061989	3.243241	8(9)-epoxy-5z,11z,14z-eicosatrienoic acid, methyl ester
M396T78	Positive ionization	136478.3696	207554.6	0.604819	1.389134	N-oleyl-leucine
M249T396	Positive ionization	4136994.307	3222001	-0.36063	4.311108	Thr-Glu
M259T447	Positive ionization	3265262.742	2503225	-0.38341	4.102738	D-pyroglutamic acid
M180T42	Positive ionization	953064.5021	717832.3	-0.40893	2.104472	.gamma.-aminobutyric acid
M420T39	Positive ionization	604492.879	303704	-0.99306	2.192372	Terpestacin
M152T211	Positive ionization	64022.8229	122405.7	0.935013	1.374758	Methyl 2-aminobenzoate
M770T232	Positive ionization	184119.9722	108136.7	-0.76779	1.142982	2-[2-[19-acetamido-6-(3,4-dicarboxybutanoyloxy)-16,18-dihydroxy-5,9-dimethylicosan-7-yl]oxy-2-oxoethyl]butanedioic aci
M166T360	Positive ionization	296448.3586	214116	-0.46939	1.16112	Methionine sulfoxide
M277T447	Positive ionization	2991345.907	2337200	-0.35601	3.775834	Gamma-l-glutamyl-l-glutamic acid
M144T269	Positive ionization	514450.7163	684789.1	0.412627	2.350372	Stachydrine

M288T331	Positive ionization	2400162.876	1963657	-0.28959	2.601324	Ile-Arg
M304T377	Positive ionization	329329.147	257445.5	-0.35526	1.227816	Val-Trp
M218T515	Positive ionization	27568.57984	73508.54	1.414887	1.103607	N-.alpha.-(tert-butoxycarbonyl)-l-valine
M348T398	Positive ionization	201846.497	341839.5	0.760061	2.099464	Adenosine 2'-monophosphate
M174T126	Positive ionization	915097.4147	582347.3	-0.65205	2.364466	Vatalanib
M224T96	Positive ionization	241943.9588	88864.08	-1.445	1.684892	Araguspongin b
M377T39_1	Positive ionization	2671910.153	1740539	-0.61834	4.923332	2-(14,15-epoxyeicosatrienoyl)glycerol
M327T63	Positive ionization	404317.1882	865236.2	1.097606	4.056011	10-hydroxy-4z,7z,11e,13z,16z,19z-docosahexaenoic acid
M530T158	Positive ionization	522114.7989	335013.7	-0.64015	1.880603	(2e,6e,12e)-18-(2,6-dioxopiperidin-4-yl)-9,11-dihydroxy-8-methoxy-10,12,14-trimethyl-15-oxooctadeca-2,6,12-trienoic acid
M303T39	Positive ionization	217905.4747	482073.4	1.14555	3.099295	5s-hydroxy-6e,8z,11z,14z-eicosatetraenoic acid
M302T242	Positive ionization	1155363.74	945899.6	-0.28859	1.532503	Leu-Gly-Leu
M271T409	Positive ionization	478265.9737	393373.9	-0.28191	1.098878	His-Asp
M231T196	Positive ionization	7286623.521	5703652	-0.35336	4.138895	Val-Ile
