# Supplementary material for: Integrated application of transcriptomics and metabolomics provides insights into acute hepatopancreatic necrosis disease resistance of Pacific white shrimp Litopenaeus vannamei
Source: mSystems. 2023 Jun 26;8(4):e00067-23. doi: 10.1128/msystems.00067-23 (PMC10469596; doi:10.1128/msystems.00067-23)
Supplement: TABLE S5 — DEGs and DMs responded to AHPND infection in susceptible family. [file msystems.00067-23-s0009.pdf]

Table S5. DEGs and DMs responded to AHPND infection in susceptible family

Gene_ID	S20507-0h	S20507-12h	log2(fc)	FDR	Description
MSTRG.14534	16.29333	78.20333	2.262948	9.64E-16	zonadhesin-like [Penaeus vannamei]
LVAN01890	4.53	0.08	-5.82337	9.64E-16	200 kDa antigen p200, partial [Babesia bigemina]
LVAN18894	16.41	0.001	-14.0023	7.92E-12	PREDICTED: carboxypeptidase B-like [Hyalella azteca]
LVAN00179	12.95	0.043333	-8.22326	5.39E-11	PREDICTED: FK506-binding protein 4-like [Megachile rotundata]
LVAN22415	2173.21	813.2067	-1.41813	9.39E-11	PREDICTED: adenosylhomocysteinase-like isoform X1 [Hyalella azteca]
LVAN20011	483.3167	174.3033	-1.47137	1.31E-10	-
MSTRG.31743	6.033333	0.001	-12.5587	1.63E-10	neuropilin-1-like isoform X1 [Penaeus vannamei]
LVAN04946	4.606667	0.24	-4.26262	2.02E-09	Chitooligosaccharidolytic beta-N-acetylglucosaminidase [Orchesella cincta]
LVAN03218	898.27	384.6133	-1.22374	2.78E-09	PREDICTED: S-adenosylmethionine synthase-like [Hipposideros armiger]
LVAN09590	3.413333	0.283333	-3.59061	4.18E-09	PREDICTED: protein SpAN-like isoform X2 [Hyalella azteca]
MSTRG.252	3.926667	0.001	-11.9391	5.02E-09	-
LVAN19903	9.616667	0.026667	-8.49436	1.36E-08	PREDICTED: uncharacterized protein LOC108668843, partial [Hyalella azteca]
LVAN05801	0.866667	0.001	-9.75933	3.14E-08	PREDICTED: probable chitinase 3 isoform X1 [Hyalella azteca]
LVAN16507	4.59	0.446667	-3.36122	5.32E-08	PREDICTED: platelet binding protein GspB-like [Xenopus tropicalis]
LVAN25324	29.11333	10.39	-1.48648	7.33E-08	PREDICTED: LOW QUALITY PROTEIN: legumain-like [Saccoglossus kowalevskii]
MSTRG.36759	156.7333	51.87333	-1.59525	8.32E-08	uncharacterized protein LOC113827125 [Penaeus vannamei]
MSTRG.680	5.733333	0.293333	-4.28876	1.19E-07	plectin-like [Penaeus vannamei]
LVAN05368	11.8	0.413333	-4.83534	2.21E-07	PREDICTED: cuticle protein 19-like [Hyalella azteca]
MSTRG.2622	7.283333	0.001	-12.8304	3.94E-07	cylicin-1-like [Penaeus vannamei]
LVAN05507	4.39	0.223333	-4.29695	6.75E-07	-
LVAN02021	5.046667	0.046667	-6.75679	9.02E-07	PREDICTED: uncharacterized protein LOC108672540 isoform X2 [Hyalella azteca]
MSTRG.36758	11.24333	2.356667	-2.25425	1.31E-06	uncharacterized protein LOC113827127 [Penaeus vannamei]
LVAN18451	4.686667	0.001	-12.1943	1.35E-06	glycosyl-phosphatidylinositol-linked carbonic anhydrase [Carcinus maenas]

MSTRG.39855	2.003333	0.001	-10.9682	1.44E-06	uncharacterized protein LOC113829378 [Penaeus vannamei]
MSTRG.802	6.25	0.03	-7.70275	2.41E-06	neuropeptide-like protein 31 [Penaeus vannamei]
MSTRG.25712	1.33	9.326667	2.809935	2.41E-06	-
LVAN21073	7.416667	0.446667	-4.0535	5.16E-06	PREDICTED: agglutinin-like protein ARB_02240 isoform X2 [Hyalella azteca]
LVAN04619	93.38333	44.84	-1.05838	5.22E-06	juvenile hormone esterase-like carboxylesterase 1 [Eriocheir sinensis]
LVAN06157	5.976667	1.213333	-2.30037	9.38E-06	clip domain serine protease [Eriocheir sinensis]
MSTRG.640	8.893333	21.58667	1.279344	1.11E-05	neuroparsin-A-like [Penaeus vannamei]
MSTRG.29950	4.81	0.58	-3.05191	1.15E-05	monocarboxylate transporter 8-like [Penaeus vannamei]
LVAN04920	1.803333	0.136667	-3.72193	1.39E-05	protease [Homarus americanus]
LVAN19029	2.626667	0.001	-11.359	1.72E-05	-
LVAN00078	311.9467	105.5467	-1.56342	3.20E-05	PREDICTED: alkylglycerol monooxygenase-like [Amyelois transitella]
LVAN24337	47.95667	119.9367	1.322469	4.73E-05	phosphoenolpyruvate carboxykinase [Litopenaeus vannamei]
MSTRG.39084	179.0267	70.19	-1.35084	4.88E-05	methyltransferase-like protein 27 isoform X2 [Lingula anatina]
LVAN06396	4.64	0.001	-12.1799	6.20E-05	-
LVAN24338	4.62	12.78333	1.468299	6.92E-05	phosphoenolpyruvate carboxykinase [Litopenaeus vannamei]
LVAN18483	11.52	28.71	1.317413	6.92E-05	PREDICTED: enhancer of split mbeta protein-like [Hyalella azteca]
MSTRG.27945	2.953333	9.656667	1.709181	7.31E-05	Monocarboxylate transporter 12, partial [Armadillidium vulgare]
LVAN09029	0.826667	0.001	-9.69116	8.09E-05	PREDICTED: aminopeptidase N-like [Hyalella azteca]
LVAN19500	2.106667	7.34	1.800818	8.76E-05	Calcium-activated chloride channel regulator 2 [Daphnia magna]
LVAN25519	2.543333	0.001	-11.3125	0.000107	uncharacterized LOC106101566 precursor [Papilio polytes]
MSTRG.33539	14.46333	6.076667	-1.25105	0.000126	hypothetical protein HELRODRAFT_127854, partial [Helobdella robusta]
MSTRG.33067	1.433333	0.001	-10.4852	0.00019	uncharacterized protein LOC113824167 [Penaeus vannamei]
MSTRG.24861	10.47333	3.613333	-1.53532	0.000288	-
MSTRG.30024	2.65	0.03	-6.46489	0.000301	PREDICTED: probable pathogenesis-related protein ARB_02861 [Hyalella azteca]
MSTRG.40881	6.44	0.403333	-3.99702	0.00038	cuticle protein AM/CP1114-like [Penaeus vannamei]
LVAN20738	0.716667	0.163333	-2.13348	0.000446	PREDICTED: fibrocystin-L [Strongylocentrotus purpuratus]

LVAN08663	2.4	0.026667	-6.49185	0.000446	PREDICTED: thread biopolymer filament subunit alpha-like isoform X1 [Hyalella azteca]
LVAN16409	218.4067	451.5633	1.047911	0.000542	Esterase FE4 [Orchesella cincta]
LVAN19167	1.836667	0.056667	-5.01845	0.000561	gastrolith protein [Cherax quadricarinatus]
LVAN14985	69.10667	172.9467	1.32343	0.000578	Golgi-associated plant pathogenesis-related protein 1 [Zootermopsis nevadensis]
LVAN06266	0.55	0.046667	-3.55897	0.000607	PREDICTED: sushi, von Willebrand factor type A, EGF and pentraxin domain-containing protein 1 [Ceratina calcarata]
LVAN08662	0.916667	0.001	-9.84025	0.000607	PREDICTED: agglutinin-like protein ARB_02240 isoform X2 [Hyalella azteca]
LVAN20370	6.373333	1.56	-2.0305	0.000709	-
LVAN20722	1.673333	0.143333	-3.54528	0.000734	PREDICTED: low-density lipoprotein receptor-related protein 2-like, partial [Parasteatoda tepidariorum]
LVAN14978	64.05	175.2733	1.452336	0.000774	Golgi-associated plant pathogenesis-related protein 1 [Zootermopsis nevadensis]
LVAN07013	1.39	0.016667	-6.38198	0.00081	chitin deacetylase 1 [Penaeus monodon]
LVAN01996	0.8	0.01	-6.32193	0.001117	AGAP004124-PA [Anopheles gambiae str. PEST] [Anopheles gambiae]
LVAN08433	0.416667	3.67	3.138814	0.001411	vitellogenin [Metapenaeus ensis]
LVAN06120	22.38	10.29667	-1.12003	0.001434	juvenile hormone esterase-like carboxylesterase 1 [Eriocheir sinensis]
LVAN07199	3.526667	0.043333	-6.34668	0.001788	PREDICTED: CD209 antigen-like [Hyalella azteca]
LVAN01889	1.263333	0.016667	-6.24413	0.001829	-
LVAN00487	1.496667	0.1	-3.90368	0.001844	trypsin [Euphausia superba]
LVAN04947	5.916667	0.89	-2.73291	0.001844	Chitooligosaccharidolytic beta-N-acetylglucosaminidase [Orchesella cincta]
LVAN18893	2.07	0.001	-11.0154	0.001901	-
MSTRG.31849	29.46333	12.25333	-1.26575	0.002586	-
LVAN19227	1.356667	0.3	-2.17703	0.003078	hypothetical protein g.8709 [Clastoptera arizonana]
LVAN08431	1.406667	5.086667	1.85444	0.003454	vitellogenin [Metapenaeus ensis]
LVAN20792	0.406667	1.423333	1.807355	0.003844	PREDICTED: mucin-5AC [Capra hircus]
LVAN06150	0.223333	0.001	-7.80305	0.004356	PREDICTED: uncharacterized protein LOC108678499 [Hyalella azteca]
LVAN24866	1.13	0.001	-10.1421	0.004473	AAEL007579-PA [Aedes aegypti]

LVAN12304	7.993333	2.026667	-1.97969	0.004569	-
LVAN03081	0.496667	0.001	-8.95613	0.004569	-
MSTRG.32955	17.04	7.79	-1.12923	0.004569	glycogenin-1-like [Penaeus vannamei]
MSTRG.18187	22.63	49.73	1.13588	0.004719	LOW QUALITY PROTEIN: transcription factor kayak-like [Penaeus vannamei]
LVAN03673	0.81	0.013333	-5.92481	0.005689	PREDICTED: mucin-5AC-like isoform X1 [Hyalella azteca]
MSTRG.40876	6.1	0.46	-3.7291	0.00575	endocuticle structural glycoprotein SgAbd-1-like [Penaeus vannamei]
LVAN21074	3.693333	0.443333	-3.05846	0.00575	-
LVAN12499	0.456667	0.006667	-6.09803	0.00575	-
MSTRG.2325	1.14	0.001	-10.1548	0.005773	pupal cuticle protein 36-like [Penaeus vannamei]
LVAN21698	40.05667	10.17667	-1.97678	0.005968	PREDICTED: flavin-containing monooxygenase FMO GS-OX4-like [Lingula anatina]
LVAN03951	14.02667	37.83	1.431359	0.00598	NADPH oxidase [Marsupenaeus japonicus]
MSTRG.38511	0.16	0.003333	-5.58496	0.006664	uncharacterized protein LOC113803251 [Penaeus vannamei]
MSTRG.18203	0.96	0.2	-2.26303	0.006825	-
MSTRG.7775	10.07	30.91	1.61801	0.006845	uncharacterized protein LOC113805200 [Penaeus vannamei]
MSTRG.17762	48.86333	134.95	1.465601	0.007217	-
LVAN02078	1.48	0.236667	-2.64467	0.007217	-
LVAN23505	3.113333	13.92333	2.160973	0.00777	crustacyanin subunit A [Fenneropenaeus merguiensis]
LVAN10271	70.74333	193.31	1.45025	0.008459	frazzled protein [Anopheles darlingi]
LVAN08430	0.166667	2.94	4.140779	0.008636	vitellogenin, partial [Penaeus monodon]
LVAN20791	1.306667	2.716667	1.055946	0.009056	PREDICTED: mucin-12-like, partial [Hyalella azteca]
MSTRG.17967	0.79	0.001	-9.62571	0.009121	-
MSTRG.29888	3.616667	7.836667	1.115579	0.009489	-
LVAN25505	0.22	0.001	-7.78136	0.010664	PREDICTED: proteoglycan 4-like [Papilio polytes]
LVAN08568	10.72333	28.53333	1.411895	0.011231	Leukocyte receptor cluster member 9 [Caligus rogercresseyi]
MSTRG.25390	0.673333	0.001	-9.39518	0.012022	-
LVAN11429	43.38	101.6167	1.228035	0.012941	pacifastin heavy chain [Macrobrachium rosenbergii]

MSTRG.2842	12.56667	4.526667	-1.47308	0.014643	-
MSTRG.33658	0.56	0.001	-9.12928	0.015711	Chitin binding domain [Trinorchestia longiramus]
MSTRG.33445	2.48	0.616667	-2.00778	0.015941	Reverse transcriptase domain, partial [Trinorchestia longiramus]
MSTRG.16095	1.173333	0.001	-10.1964	0.015941	probable H/ACA ribonucleoprotein complex subunit 1 [Penaeus vannamei]
LVAN25499	20.95667	8.633333	-1.27942	0.016687	PREDICTED: transmembrane protein 53-like [Hyalella azteca]
MSTRG.35147	14.83333	35.01333	1.239062	0.016687	GRIP and coiled-coil domain-containing protein 2-like [Penaeus vannamei]
MSTRG.31536	0.473333	2.703333	2.513811	0.016863	uncharacterized protein LOC113823067 [Penaeus vannamei]
MSTRG.22242	2.56	5.306667	1.051662	0.018164	uncharacterized protein LOC113819856 isoform X3 [Penaeus vannamei]
MSTRG.30088	4.66	12.18333	1.386507	0.019795	gastrula zinc finger protein XlCGF57.1-like [Danio rerio]
LVAN08436	1.01	4.073333	2.011855	0.019901	vitellogenin [Metapenaeus ensis]
LVAN14229	0.65	0.013333	-5.60733	0.020114	molting fluid carboxypeptidase A precursor [Acyrthosiphon pisum]
MSTRG.23502	0.001	0.59	9.204571	0.020511	-
LVAN09099	11.82333	27.91333	1.239318	0.021481	PREDICTED: transcriptional coactivator YAP1-like isoform X5 [Hyalella azteca]
LVAN06274	1.246667	0.001	-10.2839	0.02258	-
LVAN01901	0.313333	0.013333	-4.55459	0.023183	Chitinase-3-like protein 3 [Zootermopsis nevadensis]
MSTRG.24859	6.953333	3.103333	-1.16389	0.023183	-
MSTRG.5242	1.6	0.08	-4.32193	0.023307	gamma-interferon-inducible lysosomal thiol reductase-like [Penaeus vannamei]
MSTRG.41621	0.816667	0.016667	-5.61471	0.023382	uncharacterized protein LOC113800069 [Penaeus vannamei]
LVAN23498	0.97	6.76	2.800967	0.023779	crustacyanin subunit C [Fenneropenaeus merguiensis]
LVAN04884	0.33	0.006667	-5.62936	0.024343	PREDICTED: LOW QUALITY PROTEIN: mucin-5AC [Drosophila busckii]
LVAN22290	0.001	1.456667	10.50846	0.024617	-
MSTRG.29756	0.63	0.03	-4.39232	0.025306	CUB and sushi domain-containing protein 3-like isoform X1 [Penaeus vannamei]
MSTRG.30533	12.9	4.98	-1.37315	0.025641	keratin, type I cytoskeletal 9-like [Penaeus vannamei]
MSTRG.1424	6.333333	13.67	1.109976	0.025641	zinc finger protein 853-like isoform X3 [Penaeus vannamei]
MSTRG.28617	0.603333	0.001	-9.23681	0.025673	Dynamin [Toxocara canis]
LVAN04890	0.836667	2.74	1.711451	0.026144	PREDICTED: RNA-binding protein MEX3B-like [Hyalella azteca]

LVAN17110	0.053333	1.563333	4.873444	0.029018	-
LVAN03672	0.486667	0.001	-8.92679	0.029018	PREDICTED: mucin-5AC-like isoform X2 [Hyalella azteca]
MSTRG.31336	2.186667	0.166667	-3.7137	0.0303	neurotrophin 1-like [Penaeus vannamei]
MSTRG.31826	0.796667	4.073333	2.354162	0.0303	-
MSTRG.38898	111.46	239.14	1.101329	0.034216	uncharacterized protein LOC113828761 [Penaeus vannamei]
LVAN14808	0.83	0.001	-9.69697	0.036212	-
LVAN01695	70.43333	29.39	-1.26093	0.037048	-
LVAN16442	3.736667	14.29667	1.935855	0.037705	Aminopeptidase YwaD [Orchesella cincta]
LVAN05331	15.58	5.513333	-1.4987	0.03981	PREDICTED: sulfotransferase 1A1-like isoform X1 [Hyalella azteca]
LVAN19259	0.753333	0.001	-9.55714	0.040039	PREDICTED: C-type lectin domain family 3 member A-like isoform X1 [Branchiostoma belcheri]
MSTRG.25378	0.923333	0.001	-9.85071	0.040571	-
MSTRG.41674	254.1933	123.4233	-1.04231	0.040861	neurogenic locus notch homolog protein 1-like isoform X4 [Penaeus vannamei]
LVAN23499	9.95	31.71333	1.672321	0.040861	crustacyanin subunit C [Fenneropenaeus merguiensis]
MSTRG.33343	4.733333	0.526667	-3.16789	0.041305	cuticle protein 18.6-like [Penaeus vannamei]
MSTRG.43263	0.656667	4.166667	2.665661	0.041305	NADPH oxidase [Penaeus vannamei]
MSTRG.23707	7.626667	3.666667	-1.05658	0.041305	zinc knuckle protein [Penaeus vannamei]
MSTRG.24254	3.89	1.173333	-1.72916	0.041305	zinc finger protein OZF-like [Penaeus vannamei]
LVAN23455	51.23333	21.55667	-1.24895	0.041305	thioredoxin 1 [Litopenaeus vannamei]
LVAN04508	0.001	1.126667	10.13785	0.041305	triosephosphate isomerase [Penaeus monodon]
LVAN13453	0.806667	0.001	-9.65583	0.042177	-
LVAN09257	39.03333	79.83333	1.032285	0.044414	PREDICTED: hexokinase type 2-like isoform X2 [Hyalella azteca]
LVAN12472	4.28	1.27	-1.75278	0.045845	juvenile hormone esterase-like carboxylesterase 1 [Eriocheir sinensis]
MSTRG.19941	2.313333	0.596667	-1.95498	0.046132	phosphopantothenoylcysteine decarboxylase subunit VHS3-like [Penaeus vannamei]
LVAN24914	1.843333	5	1.439611	0.049014	Collagen alpha-1(XI) chain [Daphnia magna]

Metabolite_ID	Modes	S20507-0h	S20507-12h	Log2(fc)	VIP	Description
M129T387_2	Negative ionization	189376.575	494521.3941	1.384775	4.692551	Citraconic acid
M147T387	Negative ionization	208409.733	612814.5697	1.556028	5.380642	(S)-2-Hydroxyglutarate
M344T317	Negative ionization	54463.4141	35242.29078	-0.62798	1.181894	Guanosine 3',5'-cyclic monophosphate
M609T414	Negative ionization	34538.1438	18385.50663	-0.90962	1.068998	Cytidine 2',3'-cyclic monophosphoric acid
M173T281_2	Negative ionization	866157.163	595950.0243	-0.53944	4.032236	Gly-Val
M384T461	Negative ionization	78297.202	37945.19626	-1.04504	1.630784	Cytidine 5'-diphosphate
M187T249_2	Negative ionization	539399.127	370836.6896	-0.54057	3.058495	Val-Ala
M401T241	Negative ionization	59449.8536	37520.11464	-0.66401	1.213996	Finasteridecarboxylic acid
M362T434	Negative ionization	55985.6466	27989.4878	-1.00017	1.149646	Guanosine 5'-diphospho-.beta.-l-fucose
M267T291	Negative ionization	128162.825	89096.12075	-0.52454	1.431252	Leu-His
M131T315	Negative ionization	104722.33	81040.64942	-0.36985	1.12099	Gly-Gly
M180T292_2	Negative ionization	2294354	1827854.039	-0.32794	4.917671	N-acetyl-l-tyrosine
M133T402	Negative ionization	67929.7836	153932.1092	1.180178	2.395474	Malate
M385T449	Negative ionization	70275.3197	38066.32765	-0.8845	1.36379	Uridine 5'-diphosphate
M115T399	Negative ionization	71648.8348	101607.7975	0.503996	1.299729	Fumarate
M179T295_2	Negative ionization	380207.267	186747.244	-1.0257	3.171166	D-(+)-mannose
M191T346	Negative ionization	147098.66	124716.3812	-0.23813	1.097268	Ser-Ser
M259T354	Negative ionization	1654485.56	1363323.15	-0.27926	3.974502	Glu-Leu
M180T316	Negative ionization	805145.297	544696.9783	-0.5638	3.063102	Tyrosine
M219T312	Negative ionization	545967.887	466623.547	-0.22656	2.208462	Thr-Thr
M88T336_2	Negative ionization	667422.42	725050.566	0.119481	2.208556	Sarcosine
M276T55	Negative ionization	6150962.73	10369671.42	0.753486	18.04456	Arg-cys
M324T127	Negative ionization	2004191.93	1433889.791	-0.48309	5.939541	Cycloxydime
M195T411	Negative ionization	59331.8326	35712.49113	-0.73238	1.01875	1,2-propanediol, 3-(1,3-benzodioxol-5-yl)-
M556T270	Positive ionization	52701.0412	76519.9922	0.538005	1.171752	.beta.-d-glucopyranosiduronic acid, 5-[3-(1-naphthalenylcarbonyl)-

						1h-indol-1-yl]pentyl
M306T318	Positive ionization	122433.644	80134.35916	-0.61151	1.617568	Cytidine 2',3'-cyclic phosphate
M95T296	Positive ionization	187448.653	165395.0592	-0.18058	1.067125	Phenol
M279T261	Positive ionization	4209011.51	1392080.668	-1.59624	12.14444	3-amino-1-propanesulfonic acid
M177T51	Positive ionization	350717.99	711931.6396	1.021427	4.399719	Dodecanedioic acid
M659T369	Positive ionization	45966.2466	23680.46898	-0.95688	1.077618	Adenosine 3',5'-cyclic monophosphate
M152T258_2	Positive ionization	12425584.8	15032606.95	0.274781	11.57895	Guanine
M361T365	Positive ionization	137034.877	104353.7062	-0.39306	1.275791	Thr-Leu-Lys
M90T339	Positive ionization	317936.938	344042.0871	0.113844	1.099545	Alanine
M346T319_1	Positive ionization	132058.218	91931.50332	-0.52254	1.502486	Cyclic gmp
M327T352	Positive ionization	490167.148	423195.0284	-0.21195	1.760956	Bilobalide
M435T397	Positive ionization	90746.6549	50744.079	-0.8386	1.390487	Glu-Met-Arg
M655T456	Positive ionization	228819.568	90199.36606	-1.34302	2.616363	Coproporphyrin I
M286T162	Positive ionization	91380.5135	148385.6464	0.699393	1.669726	N-acetylcytidine
M126T223	Positive ionization	74714.6101	134650.5106	0.849757	1.720232	5-methyl-2'-deoxycytidine
M264T294	Positive ionization	84701.8705	61483.08104	-0.4622	1.064207	Met-Asn
M187T406	Positive ionization	552625.476	437433.2264	-0.33724	2.300138	Pyroglutamylglycine
M529T379	Positive ionization	86418.4309	52692.61263	-0.71374	1.227045	Chaetoglobosin c
M207T287	Positive ionization	316978.708	270138.4449	-0.23069	1.58495	Gly-Met
M558T469	Positive ionization	66788.664	41354.36309	-0.69156	1.127858	Penitrem a
M258T170	Positive ionization	692089.671	1185465.689	0.776423	4.632753	2'-o-methylcytidine
M116T264	Positive ionization	34484.8182	68098.3125	0.981658	1.376811	3-dehydrocarnitine
M364T433_1	Positive ionization	132592.163	72045.38119	-0.88002	1.598058	Guanosine 5'-monophosphate
M284T258	Positive ionization	8724148.68	10607012.57	0.281932	9.472182	His-Lys
M489T436	Positive ionization	34761.4392	9418.802293	-1.88387	1.048914	Cytidine 5'-diphosphocholine
M363T245	Positive ionization	35963.7854	63211.4969	0.813642	1.143192	Cinobufotalin

M399T494_1	Positive ionization	63571.9885	97615.3072	0.618716	1.226198	S-adenosyl-l-methionine
M248T385	Positive ionization	819256.81	257255.5935	-1.67111	4.564895	Malonyl-l-carnitine
M126T290_2	Positive ionization	6756481.3	8404015.967	0.314807	9.948117	Taurine
M300T40	Positive ionization	1098679.4	1835179.848	0.740151	6.257773	Palmitoyl ethanolamide
M166T225_2	Positive ionization	165422.501	66970.35721	-1.30456	2.002986	7-methylguanine
M190T372	Positive ionization	162809.362	140457.3604	-0.21305	1.089262	Gly-Asn
M182T296_2	Positive ionization	5889569.91	5271743.25	-0.15988	4.570601	DL-tyrosine
M426T170_2	Positive ionization	5128498.23	999793.5406	-2.35883	13.60826	Oleoyl-l-carnitine
M258T223	Positive ionization	62266.042	107656.3089	0.789915	1.458803	5-methylcytidine
M582T391	Positive ionization	172426.853	103014.4436	-0.74314	1.820012	Ergotamine
M136T296_2	Positive ionization	4154588.78	3696992.55	-0.16835	3.900293	Dl-octopamine
M401T38	Positive ionization	123856.162	77453.89159	-0.67726	1.543729	7.alpha.,27-dihydroxycholesterol
M496T455	Positive ionization	105844.398	57000.08163	-0.89291	1.322989	Cytochalasin e
M298T103	Positive ionization	767562.031	1358994.612	0.824184	5.1527	S-methyl-5'-thioadenosine
M175T282	Positive ionization	681024.902	492086.147	-0.4688	3.05464	Val-Gly
M209T428	Positive ionization	39346.1287	103232.2453	1.3916	1.587208	Dl-lanthionine
M404T439	Positive ionization	264153.041	202119.2518	-0.38617	1.64386	Thr-Gln-Arg
M227T416	Positive ionization	294649.765	246817.4121	-0.25556	1.417927	N-(aminothioxomethyl)-5-oxo-1-propyl-2-pyrrolidineacetamide
M704T177	Positive ionization	265094.085	192626.2646	-0.4607	1.840963	Palmitoyl sphingomyelin
M268T167	Positive ionization	961274.739	574687.7704	-0.74217	4.280651	Adenosine
M281T220	Positive ionization	372214.69	291446.1241	-0.35291	2.013434	Val-Tyr
M391T222	Positive ionization	27428.6802	53675.86483	0.968588	1.11601	Prostaglandin g2
M789T46	Positive ionization	164282.526	133502.0827	-0.29932	1.164492	1,2-dioleoyl-sn-glycero-3-phospho-l-serine
M642T174	Positive ionization	45140.4762	75872.09428	0.749148	1.198008	Dodecanamide, n-[(1s,2r,3e)-2-hydroxy-1-(hydroxymethyl)-3-heptadecen-1-yl]-12-[(7-nitro-2,1,3-benzoxadiazol-4-yl)amino]-
M243T352	Positive ionization	522783.941	416902.4288	-0.32651	2.28278	Ser-His

M135T258	Positive ionization	314252.284	366549.5179	0.222085	1.463259	2-dimethylamino-6-hydroxypurine
M285T406	Positive ionization	2350775.06	2170435.324	-0.11515	2.729656	His-Glu
M232T525	Positive ionization	344038.887	111161.1327	-1.62992	3.277061	N-.alpha.-(tert-butoxycarbonyl)-l-isoleucine
M279T361	Positive ionization	521827.132	453931.0122	-0.2011	1.762938	gamma-Glutamyl-L-methionine
M387T295	Positive ionization	208234.154	151401.1808	-0.45983	1.490278	Bufalin
M231T196	Positive ionization	8779196.18	7286623.521	-0.26884	8.322123	Val-Ile
M261T356	Positive ionization	1804943.67	1560169.26	-0.21025	3.463195	Ile-Glu
M118T294	Positive ionization	8890111.93	5743356.497	-0.63031	10.87312	DL-valine
M180T285	Positive ionization	77561.78	114245.0695	0.558714	1.237166	Isoxanthopterin
M325T431	Positive ionization	144747.471	90948.64594	-0.67041	1.396525	Uridine 5'-monophosphate
M232T305	Positive ionization	347712.584	260488.6514	-0.41667	1.916441	Val-Asn
M558T433	Positive ionization	20729.4506	66882.01629	1.689936	1.529622	Hexanamide, n-[(1s,2r,3e)-2-hydroxy-1-(hydroxymethyl)-3-heptadecen-1-yl]-6-[(7-nitro-2,1,3-benzoxadiazol-4-yl)amino]-
M373T230	Positive ionization	96420.7764	74572.85037	-0.37069	1.019876	Ile-Leu-Lys
M237T276	Positive ionization	202679.107	153405.5081	-0.40185	1.49982	Ser-Met
M400T173	Positive ionization	2653136.78	659150.006	-2.00902	8.981846	L-palmitoylcarnitine
M304T438	Positive ionization	3372339.89	2593183.994	-0.37903	5.659888	Arg-glu
M527T417	Positive ionization	122050.882	93243.3915	-0.38841	1.038846	Benzoic acid, 2-[3-[3-[(5-ethyl-4'-fluoro-2-hydroxy[1,1'-biphenyl]-4-yl)oxy]propoxy]-2-propylphenoxy]-
M219T403	Positive ionization	3079415.15	2803312.499	-0.13552	3.916595	5-L-Glutamyl-L-alanine
M298T195	Positive ionization	231384.756	285788.3218	0.304653	1.369454	2-o-methylguanosine
M274T66	Positive ionization	7981843.08	6721887.612	-0.24786	6.878514	Fenpropidin
M293T456	Positive ionization	74765.3039	106286.4623	0.507517	1.182623	Ethylenediaminetetraacetic acid
M423T245	Positive ionization	52507.5585	27160.09174	-0.95104	1.066703	Ginsenoside f1
M204T364	Positive ionization	469480.525	424279.8815	-0.14605	1.507947	N,n'-diacetylchitobiose
M238T172	Positive ionization	27073.9112	57601.11413	1.089193	1.020073	N-(phenylacetyl)-l-phenylalanine

M277T441	Positive ionization	196462.629	464234.7023	1.240599	2.799932	L-saccharopine
M233T56	Positive ionization	2547811.06	1929779.498	-0.40082	4.281597	Thr-Ile
M460T404	Positive ionization	224845.491	193806.7099	-0.21432	1.216593	Trp-Val-Arg
M387T235	Positive ionization	146994.166	96647.23749	-0.60496	1.544097	Val-Ile-Arg
M479T165	Positive ionization	155518.354	202275.7179	0.379238	1.231785	Turkesterone
M284T362	Positive ionization	671359.253	476583.0772	-0.49436	2.90978	His-Gln
M389T467	Positive ionization	119833.444	94318.22651	-0.34542	1.063666	Asn-Gln-Lys
