# Supplementary material for: Integrated application of transcriptomics and metabolomics provides insights into acute hepatopancreatic necrosis disease resistance of Pacific white shrimp Litopenaeus vannamei
Source: mSystems. 2023 Jun 26;8(4):e00067-23. doi: 10.1128/msystems.00067-23 (PMC10469596; doi:10.1128/msystems.00067-23)
Supplement: TABLE S6 — DEGs and DMs responded to AHPND infection in resistant family. [file msystems.00067-23-s0010.pdf]

Table S6. DEGs and DMs responded to AHPND infection in resistant family

Gene_ID	R20523-0h	R20523-12h	log2(fc)	FDR	Description
MSTRG.27945	0.876667	15.67	4.159833	3.90E-50	Monocarboxylate transporter 12, partial [Armadillidium vulgare]
LVAN10749	4.696667	99.59	4.406292	1.72E-41	PREDICTED: cold shock-induced protein TIR1 [Drosophila ficusphila]
LVAN19501	45.52	371.33	3.028129	1.52E-33	Calcium-activated chloride channel regulator 2 [Daphnia magna]
MSTRG.4708	1.426667	19.83667	3.797449	4.05E-33	-
LVAN08146	6.14	93.76	3.932662	9.19E-31	serine proteinase inhibitor [Litopenaeus vannamei]
LVAN08930	4.7	29.3	2.640168	1.18E-30	PREDICTED: serine/threonine-protein kinase DCLK1-like [Hyalella azteca]
LVAN10801	1.823333	33.08667	4.1816	6.37E-29	trehalose-6-phosphate synthase [Fenneropenaeus chinensis]
LVAN09257	21.62	215.23	3.315441	1.18E-27	PREDICTED: hexokinase type 2-like isoform X2 [Hyalella azteca]
MSTRG.3622	5.23	60.07333	3.521842	5.63E-27	uncharacterized protein LOC113826500 [Penaeus vannamei]
MSTRG.35259	4.54	42.45	3.225	1.23E-24	uncharacterized protein LOC113825950 [Penaeus vannamei]
LVAN15821	0.393333	17.15333	5.446593	4.33E-24	PREDICTED: sodium-dependent nutrient amino acid transporter 1-like [Hyalella azteca]
LVAN21984	4.06	61.61667	3.923769	1.94E-23	PREDICTED: peritrophin-1-like [Orussus abietinus]
LVAN14777	25.98333	141.6933	2.447113	2.45E-22	PREDICTED: methionine synthase [Apteryx australis mantelli]
LVAN14747	15.83	212.76	3.748494	8.99E-22	PREDICTED: methylenetetrahydrofolate reductase-like [Hyalella azteca]
LVAN01785	6.09	28.21333	2.211863	9.41E-21	-
LVAN11759	42.76333	479.0633	3.48577	1.58E-20	fructose 1,6-biphosphate-aldolase A [Fenneropenaeus chinensis]
LVAN17732	3.986667	26.91667	2.755245	2.25E-20	-
LVAN10211	18.06	225.95	3.645134	5.29E-20	PREDICTED: lactoylglutathione lyase [Crassostrea gigas]
LVAN03951	6.28	44.73	2.832406	6.26E-20	NADPH oxidase [Marsupenaeus japonicus]
LVAN14985	49.12667	259.18	2.399376	1.22E-19	Golgi-associated plant pathogenesis-related protein 1 [Zootermopsis nevadensis]
LVAN21977	24.22667	363.39	3.906851	5.48E-19	PREDICTED: mucin-5AC-like isoform X2 [Cephus cinctus]
LVAN14978	44.09	224.69	2.349413	7.17E-19	Golgi-associated plant pathogenesis-related protein 1 [Zootermopsis nevadensis]
MSTRG.29408	12.70667	93.67	2.882001	1.38E-18	Protein snakeskin [Amphibalanus amphitrite]

LVAN09524	4.01	30.82667	2.942505	1.60E-18	-
MSTRG.4219	5.883333	30.47667	2.372999	2.05E-18	uncharacterized protein LOC113800256 [Penaeus vannamei]
LVAN22766	838.9167	108.2	-2.95483	4.44E-18	hemocyanin [Fenneropenaeus chinensis]
LVAN22771	107.4167	12.11	-3.14895	4.65E-18	hemocyanin V4 [Litopenaeus vannamei]
LVAN15425	33.06667	122.83	1.893213	5.53E-18	-
LVAN15367	7.41	30.46333	2.039528	7.27E-18	Dystroglycan, partial [Zootermopsis nevadensis]
LVAN19739	6.45	28.75	2.156191	8.62E-18	-
MSTRG.36706	11.78	56.55	2.263187	1.29E-17	uncharacterized protein LOC113827095 [Penaeus vannamei]
LVAN10271	29.15667	353.4267	3.599513	1.68E-17	frazzled protein [Anopheles darlingi]
MSTRG.7775	9.823333	50.61	2.365138	3.33E-17	uncharacterized protein LOC113805200 [Penaeus vannamei]
MSTRG.30327	4.073333	32.49	2.995714	2.15E-16	50 kDa gamma-zein-like [Penaeus vannamei]
LVAN21062	0.606667	9.963333	4.037653	2.54E-16	ADAM metalloprotease, partial [Marsupenaeus japonicus]
MSTRG.31794	0.353333	12.25	5.115608	2.55E-16	phenoloxidase-activating factor 1-like [Penaeus vannamei]
LVAN02714	23.78667	86.77	1.867043	3.85E-16	integrin [Litopenaeus vannamei]
MSTRG.14534	23.45333	235.2167	3.326125	6.38E-16	zonadhesin-like [Penaeus vannamei]
LVAN03710	8.573333	106.33	3.632549	8.96E-16	innexin 7 [Homarus americanus]
MSTRG.30189	2.906667	16.02	2.462437	6.08E-15	unpaired-like protein, partial [Nasonia vitripennis]
LVAN17327	12.24667	54.68667	2.1588	1.39E-14	centaurin-alpha 1-like protein [Marsupenaeus japonicus]
LVAN05134	0.363333	18.38	5.660699	1.53E-14	triacylglycerol lipase [Portunus trituberculatus]
LVAN03709	2.496667	27.28	3.449769	2.36E-14	innexin 7 [Homarus americanus]
LVAN22769	732.98	157.7667	-2.21598	4.48E-14	hemocyanin subunit L2, partial [Litopenaeus vannamei]
LVAN03486	6.883333	54.03	2.972581	5.72E-14	PREDICTED: D-3-phosphoglycerate dehydrogenase-like [Hyalella azteca]
MSTRG.25385	7.543333	56.75667	2.911516	1.04E-13	-
LVAN16442	2.9	44.12667	3.927526	1.50E-13	Aminopeptidase YwaD [Orchesella cincta]
LVAN10731	1651.6	687.7933	-1.26382	1.57E-13	Lutropin-choriogonadotropic hormone receptor, partial [Penaeus monodon]
LVAN24425	6.973333	37.85	2.440373	2.02E-13	PREDICTED: hemocyte protein-glutamine gamma-glutamyltransferase-like [Hyalella

					azteca]
LVAN11163	8.236667	27.74	1.751835	2.04E-13	disintegrin and metalloproteinase domain-containing protein 10 [Eriocheir sinensis]
LVAN12749	12.41333	48.39333	1.962918	2.04E-13	calpain T [Gecarcinus lateralis]
LVAN07461	24.34	95.29667	1.969097	5.88E-13	-
LVAN13159	4.876667	53.81667	3.464086	6.18E-13	anti-lipopolysaccharide factor isoform 6 [Penaeus monodon]
MSTRG.15471	13.74	119.2933	3.11806	6.87E-13	uncharacterized protein LOC113810846 isoform X1 [Penaeus vannamei]
LVAN12627	3.23	19.91667	2.62437	8.16E-13	PREDICTED: rap1 GTPase-activating protein 1 isoform X6 [Tribolium castaneum]
LVAN08919	14.58333	70.33333	2.269888	1.09E-12	flotillin-2 [Litopenaeus vannamei]
LVAN17888	1.466667	11.13333	2.924273	1.97E-12	Nostrin [Zootermopsis nevadensis]
LVAN04355	9.663333	79.86333	3.04694	3.33E-12	sodium-dependent phosphate transporter [Daphnia pulex]
LVAN18759	384.46	168.1233	-1.19331	3.68E-12	PREDICTED: epidermal retinol dehydrogenase 2-like isoform X2 [Lingula anatina]
MSTRG.35258	502.7033	1740.897	1.792051	3.72E-12	uncharacterized protein LOC113825949 [Penaeus vannamei]
LVAN19177	5.566667	37.40667	2.74841	3.88E-12	Protein bric-a-brac 1 [Zootermopsis nevadensis]
LVAN24501	264.8633	124.0067	-1.09483	4.26E-12	PREDICTED: solute carrier family 35 member F6-like isoform X1 [Hyalella azteca]
LVAN22775	745.3867	147.0033	-2.34214	5.86E-12	hemocyanin subunit L2, partial [Litopenaeus vannamei]
LVAN07646	1.466667	17.53	3.579213	7.97E-12	-
LVAN21370	7.5	32.00667	2.09341	9.14E-12	PREDICTED: centaurin-gamma-1A-like isoform X1 [Hyalella azteca]
LVAN08174	4.31	52.15667	3.597092	9.17E-12	transcription factor ATF-b [Litopenaeus vannamei]
MSTRG.5266	0.303333	11.50333	5.245004	9.61E-12	-
LVAN23018	0.076667	4.043333	5.720802	9.80E-12	PREDICTED: tyrosine decarboxylase-like [Limulus polyphemus]
LVAN12797	70.15333	22.47667	-1.64208	1.09E-11	PREDICTED: uncharacterized protein LOC108675418 [Hyalella azteca]
LVAN02098	9.136667	31.35	1.778726	1.27E-11	PREDICTED: phosphatidylinositol 3,4,5-trisphosphate 3-phosphatase and dual-specificity protein phosphatase PTEN isoform X1 [Halyomorpha halys]
LVAN11055	0.956667	11.02667	3.526837	1.56E-11	PREDICTED: solute carrier family 28 member 3-like [Hyalella azteca]
MSTRG.4836	578.85	231.4567	-1.32245	5.60E-11	-
LVAN13444	1.903333	20.24	3.410609	6.13E-11	protein spaetzle, partial [Cherax quadricarinatus]

LVAN10647	71.5	270.4667	1.919436	6.76E-11	PREDICTED: delta-1-pyrroline-5-carboxylate synthase-like [Hyalella azteca]
MSTRG.19782	27.67	95.76333	1.791151	7.48E-11	uncharacterized protein LOC113814162 [Penaeus vannamei]
LVAN23690	7.09	28.15	1.989277	7.86E-11	transcription factor RFX4-like protein 1 [Sarcoptes scabiei]
LVAN01075	5.853333	23.36667	1.997122	7.86E-11	PREDICTED: triple functional domain protein-like [Hyalella azteca]
LVAN07381	0.001	23.83333	14.54069	1.05E-10	-
LVAN11322	2.233333	12.97	2.537908	1.21E-10	PREDICTED: probable G-protein coupled receptor B0563.6 [Hyalella azteca]
LVAN04830	1.77	7.943333	2.165995	1.26E-10	PREDICTED: inverted formin-2-like [Aplysia californica]
LVAN17723	5.333333	45.98	3.107897	1.36E-10	PREDICTED: regulator of G-protein signaling 2-like [Hyalella azteca]
LVAN08053	2.73	9.95	1.865796	1.54E-10	PREDICTED: chloride channel protein 2-like isoform X3 [Hyalella azteca]
MSTRG.14536	280.56	927.66	1.725287	2.00E-10	peritrophin-44-like protein [Penaeus vannamei]
LVAN24132	5.113333	22.52	2.138871	2.79E-10	PREDICTED: mannosylglucosyl-3-phosphoglycerate phosphatase-like isoform X2 [Hyalella azteca]
LVAN25073	57.72333	13.55	-2.09086	2.92E-10	PREDICTED: LOW QUALITY PROTEIN: regucalcin-like [Aethina tumida]
MSTRG.4835	43.58333	14.55667	-1.5821	4.01E-10	uncharacterized protein LOC113803178 [Penaeus vannamei]
MSTRG.38015	1.27	10.21667	3.008024	4.01E-10	organic solute transporter subunit alpha-like [Penaeus vannamei]
LVAN20796	1.686667	9.36	2.472334	4.67E-10	Leucine-rich repeat and immunoglobulin-like domain containing-NOGO receptor-interacting protein 4 [Zootermopsis nevadensis]
MSTRG.39654	49.67333	167.0433	1.749679	4.97E-10	-
MSTRG.25019	10.19667	42.74333	2.067602	5.73E-10	uncharacterized protein LOC113818168 [Penaeus vannamei]
LVAN00031	33.65333	84.48667	1.327974	6.48E-10	NF-kB transcription factor Relish [Penaeus monodon]
LVAN09124	5.16	31.87	2.626756	6.56E-10	La-related protein 6 [Zootermopsis nevadensis]
MSTRG.19013	0.29	21.76333	6.229703	6.89E-10	uncharacterized protein LOC113813516 [Penaeus vannamei]
LVAN18834	13.44667	39.83667	1.566848	6.98E-10	PREDICTED: probable G-protein coupled receptor B0563.6 [Hyalella azteca]
MSTRG.33562	54.65667	126.3733	1.209223	7.33E-10	very low-density lipoprotein receptor-like isoform X1 [Penaeus vannamei]
LVAN08488	26.53333	8.666667	-1.61426	7.44E-10	PREDICTED: alpha-mannosidase 2C1-like [Neolamprologus brichardi]
MSTRG.20369	0.14	6.733333	5.587822	8.57E-10	-

MSTRG.36995	5.91	26.54333	2.16712	1.49E-09	cyclin-dependent kinase inhibitor 1B-like [Penaeus vannamei]
LVAN24337	39.35	150.6433	1.936701	1.67E-09	phosphoenolpyruvate carboxykinase [Litopenaeus vannamei]
LVAN07983	12.69333	67.35667	2.40775	1.67E-09	PREDICTED: probable leucine aminopeptidase MCYG_03459-like [Saccoglossus kowalevskii]
MSTRG.29785	9.8	34.44667	1.813511	2.22E-09	zinc finger X-linked protein ZXDA-like isoform X1 [Penaeus vannamei]
LVAN03711	0.986667	13.69667	3.795118	3.24E-09	innexin 2 [Penaeus monodon]
MSTRG.21983	3.216667	12.26	1.930321	3.24E-09	LOW QUALITY PROTEIN: transcription factor SOX-4-like [Penaeus vannamei]
MSTRG.17762	61.42	265.6867	2.112945	3.24E-09	-
LVAN03978	4.923333	16.94	1.782727	3.68E-09	Tyrosine-protein phosphatase 69D, partial [Trachymyrmex zeteki]
LVAN00201	3.243333	113.91	5.134273	5.74E-09	PDGF/VEGF-related factor 1 [Eriocheir sinensis]
LVAN19593	0.83	6.576667	2.986173	6.77E-09	-
LVAN08957	0.001	4.936667	12.26932	7.02E-09	protein-l-isoaspartate(d-aspartate) o-methyltransferase-like [Stylonychia lemnae]
MSTRG.37067	0.723333	11.91667	4.042176	7.66E-09	ML protein [Penaeus japonicus]
MSTRG.36206	55.62	12.80667	-2.11871	9.87E-09	-
MSTRG.38215	0.96	5.143333	2.421597	1.07E-08	Pectin lyase fold/virulence factor [Trinorchestia longiramus]
LVAN21152	74.35	30.55333	-1.283	1.07E-08	PREDICTED: deoxynucleoside triphosphate triphosphohydrolase SAMHD1-like, partial [Saccoglossus kowalevskii]
LVAN08883	0.723333	4.863333	2.749213	1.33E-08	PREDICTED: LOW QUALITY PROTEIN: kelch repeat-containing protein 2-like [Aethina tumida]
LVAN10543	3.113333	19.92333	2.677927	1.39E-08	-
MSTRG.37081	236.0833	114.92	-1.03867	1.64E-08	hypothetical protein C7M84_009776 [Penaeus vannamei]
LVAN23214	12.34667	61.88	2.325352	1.96E-08	serine protease 1 [Litopenaeus vannamei]
MSTRG.17750	1.886667	8.63	2.193521	1.98E-08	-
LVAN05502	18.13	56.52	1.640383	2.15E-08	PREDICTED: acyl-coenzyme A synthetase ACSM3, mitochondrial-like [Branchiostoma belcheri]
LVAN08873	64.27	24.69	-1.38022	2.32E-08	PREDICTED: 4-coumarate--CoA ligase 1-like isoform X1 [Branchiostoma belcheri]

LVAN04896	1.093333	8.573333	2.971123	3.39E-08	-
MSTRG.31652	0.04	12.18333	8.250693	3.39E-08	-
MSTRG.16167	0.34	5.766667	4.084131	4.02E-08	-
LVAN03507	18.66333	334.6067	4.164188	4.07E-08	PREDICTED: uncharacterized protein LOC108667142 [Hyalella azteca]
LVAN02341	29.17333	145.48	2.318099	4.78E-08	trypsin [Euphausia superba]
LVAN20647	5.63	22.84	2.020356	5.21E-08	PREDICTED: src kinase-associated phosphoprotein 1-like [Hyalella azteca]
MSTRG.31649	0.053333	14.59333	8.096056	5.48E-08	-
LVAN10876	66.80667	22.66	-1.55984	6.10E-08	Beta,beta-carotene 9',10'-oxygenase [Daphnia magna]
LVAN24261	7.1	41.85667	2.559566	6.86E-08	serine proteinase inhibitor B3 [Litopenaeus vannamei]
MSTRG.33552	5.443333	32.73667	2.588345	7.30E-08	glycine N-acyltransferase-like protein 3 isoform X2 [Pelodiscus sinensis]
LVAN17437	38.83333	125	1.686561	8.16E-08	PREDICTED: tubulin-folding cofactor B-like [Hyalella azteca]
LVAN03242	370.57	127.9033	-1.53469	8.34E-08	PREDICTED: gamma-butyrobetaine dioxygenase-like [Saccoglossus kowalevskii]
LVAN02954	8.463333	26.67667	1.656281	8.82E-08	PREDICTED: spermatogenesis-associated protein 13-like [Limulus polyphemus]
MSTRG.36155	0.306667	6.326667	4.366702	9.26E-08	mucin-5AC-like [Penaeus vannamei]
LVAN20413	0.823333	8.13	3.303707	1.03E-07	X-linked interleukin-1 receptor accessory protein-like 1 [Zootermopsis nevadensis]
LVAN00742	0.023333	6.436667	8.107778	1.10E-07	PREDICTED: chitinase-3-like protein 1 [Parasteatoda tepidariorum]
LVAN10511	3.776667	20.98	2.473829	1.19E-07	PREDICTED: probable leucine aminopeptidase MCYG_03459-like [Saccoglossus kowalevskii]
LVAN10207	0.2	6.956667	5.120324	1.19E-07	serpin8 [Litopenaeus vannamei]
MSTRG.36154	0.676667	11.35667	4.06895	1.27E-07	mucin-5AC-like [Penaeus vannamei]
LVAN04839	28.23	7.883333	-1.84035	1.27E-07	PREDICTED: phosphatidylinositol phosphatase PTPRQ-like [Hyalella azteca]
MSTRG.19756	1.516667	5.163333	1.767399	1.27E-07	Krueppel-like factor 7 [Armadillidium vulgare]
MSTRG.1087	0.19	6.58	5.114016	1.42E-07	uncharacterized protein LOC113814559 [Penaeus vannamei]
MSTRG.13219	1.113333	7.763333	2.801791	1.77E-07	PREDICTED: uncharacterized transmembrane protein DDB_G0289901-like [Hyalella azteca]
LVAN05322	9.616667	28.82	1.583462	1.82E-07	GAS2 protein 1 [Daphnia magna]

LVAN13739	1.203333	8.903333	2.887309	1.93E-07	cyclooxygenase [Penaeus monodon]
LVAN23112	0.186667	2.226667	3.576349	1.97E-07	metabotropic glutamate receptor type 3 [Homarus americanus]
LVAN14107	24.25	58.84667	1.278976	2.35E-07	PREDICTED: unconventional myosin-Ie-like [Hyalella azteca]
MSTRG.39653	266.4367	1246.437	2.225945	2.35E-07	-
MSTRG.19114	17.74667	47.07333	1.407362	2.53E-07	-
LVAN25234	1.043333	7.693333	2.882409	2.55E-07	Class b secretin g-protein coupled receptor gprmth4 [Daphnia magna]
MSTRG.16229	16.38667	42.03333	1.359011	2.59E-07	amoeboid myosin I, partial [Penaeus vannamei]
LVAN15826	15.35	44.31333	1.529502	2.61E-07	PREDICTED: plexin A3-like isoform X2 [Hyalella azteca]
LVAN19598	1.026667	6.226667	2.600492	2.79E-07	PREDICTED: microtubule-associated protein futsch-like [Hyalella azteca]
LVAN04844	16.43333	76.22	2.213544	2.81E-07	PREDICTED: metallophosphoesterase domain-containing protein 1-like [Limulus polyphemus]
MSTRG.25558	56.20667	27.9	-1.01048	2.89E-07	-
LVAN01599	31.39667	9.056667	-1.79356	3.25E-07	PREDICTED: gamma-glutamyl hydrolase-like [Galendromus occidentalis]
LVAN22770	1557.017	536.4567	-1.53725	3.26E-07	hemocyanin [Litopenaeus vannamei]
LVAN14742	4.66	19.94667	2.097746	3.43E-07	hypothetical protein [Scylla paramamosain]
LVAN18970	17.84	42.55667	1.25427	3.43E-07	PREDICTED: lethal(2) giant larvae protein homolog 1-like [Hyalella azteca]
LVAN03284	38.65	168.1333	2.121065	3.43E-07	anti-lipopolysaccharide AV-R isoform [Litopenaeus vannamei]
MSTRG.21080	6.01	19.51667	1.69927	3.49E-07	-
LVAN22768	4.043333	0.836667	-2.27282	3.82E-07	hemocyanin subunit L2, partial [Litopenaeus vannamei]
MSTRG.17657	41.55	180.08	2.115718	4.38E-07	cilia- and flagella-associated protein 251-like isoform X1 [Penaeus vannamei]
MSTRG.15566	3.653333	23.02	2.655603	4.98E-07	uncharacterized protein LOC113810921 [Penaeus vannamei]
MSTRG.35598	79.68667	199.9633	1.327325	5.59E-07	protein enabled-like isoform X1 [Penaeus vannamei]
LVAN16256	44.22333	149.1733	1.75411	6.59E-07	PREDICTED: 1-carboxy-3-chloro-3,4-dihydroxycyclo hexa-1,5-diene dehydrogenase-like isoform X2 [Ictalurus punctatus]
LVAN17704	0.001	27.49	14.74662	6.59E-07	triosephosphate isomerase [Penaeus monodon]
LVAN21424	31.30333	13.24667	-1.24069	7.28E-07	PREDICTED: monocarboxylate transporter 13 isoform X1 [Halyomorpha halys]

LVAN05132	77.67333	34.5	-1.17082	7.74E-07	PREDICTED: probable cytochrome P450 49a1 [Hyalella azteca]
LVAN06944	8.806667	25.47333	1.53232	8.09E-07	PREDICTED: IST1 homolog [Agrilus planipennis]
LVAN03811	0.001	5.323333	12.37811	8.09E-07	triosephosphate isomerase [Penaeus monodon]
MSTRG.38197	2.656667	8.276667	1.639433	8.52E-07	protein sprouty-like, partial [Penaeus vannamei]
LVAN13060	46.2	140.7933	1.607614	8.63E-07	lactate dehydrogenase [Litopenaeus vannamei]
LVAN19875	736.34	1920.797	1.383261	8.80E-07	juvenile hormone esterase-like carboxylesterase 1 [Eriocheir sinensis]
LVAN13955	0.273333	2.83	3.372069	9.04E-07	transglutaminase II [Eriocheir sinensis]
LVAN24465	11.65667	168.4433	3.853036	9.97E-07	PREDICTED: titin isoform X1 [Ictalurus punctatus]
LVAN14160	0.77	5.273333	2.775785	1.00E-06	-
LVAN17702	0.001	39.17667	15.25771	1.05E-06	triosephosphate isomerase [Penaeus monodon]
LVAN20940	2.586667	11.34	2.132255	1.20E-06	integrin alpha 4, partial [Fenneropenaeus chinensis]
MSTRG.18966	0.593333	12.17	4.358343	1.23E-06	uncharacterized protein LOC113813488 [Penaeus vannamei]
LVAN11016	22.16333	9.48	-1.22522	1.23E-06	PREDICTED: ATP-binding cassette sub-family B member 6, mitochondrial [Anoplophora glabripennis]
LVAN08393	4607.873	2141.887	-1.10522	1.25E-06	PREDICTED: S-adenosylmethionine synthase-like [Hipposideros armiger]
LVAN03661	63.32	158.2767	1.321715	1.30E-06	PREDICTED: choline transporter-like protein 2 [Hyalella azteca]
LVAN02986	4.513333	16.71	1.888446	1.33E-06	serine proteinase inhibitor-3 [Eriocheir sinensis]
MSTRG.36209	203.4967	63.27667	-1.68526	1.53E-06	-
MSTRG.9343	15.97333	56.00333	1.809847	1.72E-06	LOW QUALITY PROTEIN: kunitz-type serine protease inhibitor HCRG1-like [Penaeus vannamei]
LVAN16195	8.59	25.75	1.583842	1.94E-06	PREDICTED: glycerol-3-phosphate dehydrogenase, mitochondrial-like [Hyalella azteca]
LVAN15388	0.95	9.926667	3.38531	1.95E-06	PREDICTED: uncharacterized protein LOC108679670 [Hyalella azteca]
MSTRG.22497	3.45	12.04333	1.803566	2.18E-06	GAS2-like protein 1, partial [Penaeus vannamei]
MSTRG.29349	45.29333	19.72333	-1.1994	2.18E-06	Concanavalin A-like lectin/glucanase domain [Trinorchestia longiramus]
LVAN15461	39.84	104.12	1.385958	2.30E-06	flotillin-1 [Scylla paramamosain]
LVAN05814	4.296667	11.91667	1.471691	2.31E-06	PREDICTED: glycerol-3-phosphate dehydrogenase, mitochondrial-like isoform X1

					[Aethina tumida]
MSTRG.28977	76.65333	196.3233	1.356811	2.35E-06	-
LVAN11056	16.86667	46.07667	1.449861	2.39E-06	PREDICTED: solute carrier family 28 member 3-like [Hyalella azteca]
MSTRG.35748	17.03667	38.35333	1.170709	2.39E-06	substance-K receptor-like isoform X1 [Penaeus vannamei]
MSTRG.32308	1314.337	161.0467	-3.02878	2.45E-06	hemocyanin C chain-like [Penaeus vannamei]
LVAN02632	2.39	61.45667	4.684487	2.54E-06	prophenoloxidase-activating enzyme 2a [Penaeus monodon]
LVAN09833	12.18	31.56667	1.373888	2.70E-06	PREDICTED: NLR family CARD domain-containing protein 4-like [Hyalella azteca]
LVAN13120	1483.41	3763.027	1.342976	3.01E-06	PREDICTED: low-density lipoprotein receptor-related protein 2-like [Hyalella azteca]
LVAN24174	0.2	3.493333	4.126532	3.40E-06	PREDICTED: 1-aminocyclopropane-1-carboxylate synthase-like protein 1 isoform X2 [Lingula anatina]
LVAN04840	37.47667	12.26667	-1.61125	3.59E-06	PREDICTED: serine/threonine-protein phosphatase 6 regulatory ankyrin repeat subunit B-like [Strongylocentrotus purpuratus]
LVAN05106	4.803333	26.46333	2.461887	3.74E-06	PREDICTED: EF-hand domain-containing protein D2 homolog isoform X2 [Hyalella azteca]
LVAN00603	11.02333	2.12	-2.37842	4.08E-06	Trimethyllysine dioxygenase, mitochondrial [Daphnia magna]
LVAN14860	195.5267	413.2567	1.079673	4.13E-06	Selenocysteine lyase [Crassostrea gigas]
LVAN06425	0.586667	12.33667	4.394268	4.83E-06	-
LVAN19799	82.6	36.27667	-1.1871	5.33E-06	PREDICTED: vacuolar protein sorting-associated protein 4B-like [Branchiostoma belcheri]
LVAN04660	0.001	2.49	11.28193	5.54E-06	Chitinase Idgf1-like protein [Daphnia magna]
MSTRG.13209	2.013333	6.403333	1.669237	5.74E-06	E3 ubiquitin-protein ligase SIAH1B-like [Penaeus vannamei]
MSTRG.36571	72.48667	20.20667	-1.84288	5.76E-06	regucalcin-like [Penaeus vannamei]
LVAN03858	41.26	20.38667	-1.01712	5.76E-06	PREDICTED: cytochrome c oxidase assembly protein COX11, mitochondrial [Bactrocera cucurbitae]
MSTRG.14731	42.29	148.29	1.810033	5.77E-06	uncharacterized protein LOC113810182 [Penaeus vannamei]
MSTRG.11151	0.001	4.223333	12.04417	5.89E-06	phosphoglucomutase [Penaeus vannamei]

LVAN19880	9.143333	29.82333	1.705649	6.00E-06	juvenile hormone esterase-like carboxylesterase 1 [Eriocheir sinensis]
MSTRG.28134	5.183333	18.95667	1.870753	6.00E-06	integrin alpha-PS3-like [Penaeus vannamei]
MSTRG.1596	1.116667	5.696667	2.350919	6.08E-06	-
LVAN02876	0.403333	3.63	3.169925	6.09E-06	-
LVAN22127	12.44667	40.73667	1.710569	6.09E-06	Kruppel-like protein, partial [Scylla paramamosain]
LVAN05704	15.36333	56.31333	1.873985	6.37E-06	-
LVAN09952	14.37667	5.166667	-1.47642	7.14E-06	PREDICTED: D-beta-hydroxybutyrate dehydrogenase, mitochondrial-like [Haplochromis burtoni]
MSTRG.1424	4.79	24.1	2.330936	7.17E-06	zinc finger protein 853-like isoform X3 [Penaeus vannamei]
MSTRG.38195	12.91	4.79	-1.43039	7.35E-06	galactoside 3(4)-L-fucosyltransferase-like, partial [Penaeus vannamei]
LVAN18110	15.36667	56.47	1.87768	7.45E-06	PREDICTED: phosphoserine phosphatase [Cimex lectularius]
LVAN06272	28.48	69.94333	1.296237	7.47E-06	PREDICTED: dystonin-like isoform X2 [Hyalella azteca]
LVAN17717	5.386667	20.34667	1.917328	7.92E-06	PREDICTED: T-complex protein 11-like protein 1 isoform X2 [Athalia rosae]
MSTRG.24739	0.001	3.906667	11.93172	8.02E-06	uncharacterized protein LOC113817936 [Penaeus vannamei]
MSTRG.266	36.09667	96.57	1.419709	8.06E-06	-
LVAN11407	0.81	6.536667	3.012561	8.44E-06	-
LVAN19519	58.60667	173.85	1.568706	8.66E-06	GDP-mannose 4,6 dehydratase [Orchesella cincta]
LVAN03638	13.28667	34.38	1.37159	9.12E-06	PREDICTED: ATP-binding cassette sub-family A member 1-like [Hyalella azteca]
MSTRG.12727	0.573333	5.4	3.235513	9.28E-06	CREB-binding protein-like isoform X1 [Penaeus vannamei]
LVAN15057	3.073333	12.08667	1.97554	9.64E-06	PREDICTED: endothelin-converting enzyme 1 [Halyomorpha halys]
MSTRG.37061	3.663333	10.7	1.546382	1.09E-05	actin cytoskeleton-regulatory complex protein pan-1-like [Penaeus vannamei]
LVAN04954	67.35333	162.44	1.270086	1.15E-05	PREDICTED: serine/threonine-protein kinase pim-3-like [Limulus polyphemus]
LVAN19589	3.733333	17.83667	2.25631	1.20E-05	G protein-coupled receptor [Procambarus clarkii]
LVAN08157	2.94	10.85	1.883807	1.21E-05	alpha-amylase, partial [Litopenaeus vannamei]
MSTRG.796	0.553333	8.263333	3.900503	1.27E-05	uncharacterized protein LOC113805115 [Penaeus vannamei]
LVAN18386	4.546667	11.59667	1.350829	1.27E-05	-

LVAN15955	0.42	10.29333	4.615177	1.29E-05	-
LVAN03302	0.553333	5.563333	3.329729	1.31E-05	serine proteinase inhibitor-2 [Eriocheir sinensis]
LVAN14614	22.67333	54.53667	1.26623	1.36E-05	PREDICTED: F-box only protein 6-like [Crassostrea gigas]
LVAN09483	72.39	167.4233	1.209638	1.44E-05	PREDICTED: alpha-tocopherol transfer protein-like [Hyalella azteca]
LVAN12117	11.99667	79.77	2.733213	1.72E-05	anti-lipopolysaccharide factor [Litopenaeus stylirostris]
LVAN16257	46.32333	143.0767	1.626977	1.72E-05	4,5-dihydroxyphthalate dehydrogenase [Salpingoeca rosetta]
MSTRG.35846	12.78667	3.186667	-2.00452	1.72E-05	-
LVAN17329	75.72333	36.49667	-1.05297	1.85E-05	cysteine dioxygenase [Litopenaeus vannamei]
LVAN06562	5.003333	19.11333	1.933618	1.87E-05	PREDICTED: ETS-related transcription factor Elf-4-like [Hyalella azteca]
LVAN14267	0.001	3.063333	11.58089	1.88E-05	hypothetical protein, partial [Scylla paramamosain]
MSTRG.6170	9.34	34.41667	1.881613	1.91E-05	carbonic anhydrase 1-like [Penaeus vannamei]
LVAN11664	0.001	16.52	14.01193	1.93E-05	PREDICTED: ETS-related transcription factor Elf-4-like [Hyalella azteca]
MSTRG.35341	3.773333	0.386667	-3.28668	1.94E-05	-
LVAN07382	4.686667	14.84333	1.663181	1.98E-05	PREDICTED: probable phosphoserine aminotransferase [Drosophila elegans]
LVAN03003	32.62333	88.75333	1.443897	2.12E-05	Glutamate synthase [NADH], amyloplastic [Zootermopsis nevadensis]
LVAN09041	0.001	3.096667	11.5965	2.41E-05	PREDICTED: ETS-related transcription factor Elf-4-like [Hyalella azteca]
LVAN15530	15.96667	50.24	1.653773	2.41E-05	-
LVAN21321	74.49	186.5567	1.324495	2.64E-05	-
LVAN21083	5.833333	19.07333	1.709165	2.80E-05	PREDICTED: integrin alpha-PS4-like isoform X1 [Microplitis demolitor]
LVAN22290	0.06	3.536667	5.881284	3.03E-05	-
LVAN10230	3.09	10.78333	1.803124	3.21E-05	PREDICTED: UDP-glucuronosyltransferase 2B14-like [Hyalella azteca]
LVAN24486	183.01	528.3133	1.529471	3.40E-05	PREDICTED: bifunctional glutamate/proline--tRNA ligase [Agrilus planipennis]
LVAN25570	7.873333	25.46333	1.693375	3.57E-05	PREDICTED: TBC1 domain family member 10A-like [Hyalella azteca]
MSTRG.36763	53.69333	22.91667	-1.22835	3.65E-05	uncharacterized protein LOC113827124 [Penaeus vannamei]
MSTRG.30299	9.17	32.69667	1.83415	3.82E-05	solute carrier family 15 member 2-like isoform X3 [Penaeus vannamei]
LVAN06563	0.433333	5.336667	3.62239	3.82E-05	PREDICTED: flocculation protein FLO11-like, partial [Hyalella azteca]

LVAN03473	0.23	13.13667	5.835822	3.93E-05	PREDICTED: uncharacterized protein LOC108669104 [Hyalella azteca]
LVAN05074	4.96	24.62667	2.311809	4.00E-05	cysteine sulfinic acid decarboxylase [Litopenaeus vannamei]
LVAN18265	3.71	15.05	2.020272	4.00E-05	PREDICTED: organic solute transporter subunit alpha-like [Hyalella azteca]
LVAN04508	0.076667	37.93333	8.950651	4.08E-05	triosephosphate isomerase [Penaeus monodon]
LVAN12625	0.001	2.873333	11.48851	4.12E-05	AGAP005712-PC [Anopheles gambiae str. PEST] [Anopheles gambiae]
LVAN04071	0.076667	1.893333	4.626185	4.16E-05	heat shock protein 21 [Macrobrachium rosenbergii]
LVAN19662	21.87	56.40333	1.366827	4.19E-05	PREDICTED: ATP-binding cassette sub-family A member 1-like [Hyalella azteca]
MSTRG.4655	1.3	8.406667	2.693022	4.23E-05	-
LVAN11153	18.96667	44.49333	1.230123	4.29E-05	PREDICTED: carboxypeptidase B-like [Hyalella azteca]
LVAN10648	0.393333	3.723333	3.24277	4.53E-05	PREDICTED: peroxisomal N(1)-acetyl-spermine/spermidine oxidase [Camponotus floridanus]
LVAN18242	0.113333	4.75	5.389283	4.70E-05	Apolipoprotein D [Acromyrmex echinatior]
LVAN24199	6.063333	25.59333	2.077585	4.99E-05	-
LVAN09052	8.416667	30.22333	1.844342	5.26E-05	PREDICTED: vascular endothelial growth factor A-like [Limulus polyphemus]
LVAN16713	0.001	5.843333	12.51258	5.70E-05	-
LVAN05544	0.973333	8.35	3.10077	5.71E-05	PREDICTED: uncharacterized protein LOC108670255 [Hyalella azteca]
LVAN14661	3.066667	7.096667	1.21047	6.09E-05	Triple functional domain protein-like Protein [Tribolium castaneum]
MSTRG.31787	9.893333	21.81	1.140461	6.24E-05	MICAL-like protein 1 [Penaeus vannamei]
LVAN07421	30.30333	10.76333	-1.49335	6.27E-05	vitelline membrane outer layer protein I-like protein [Pacifastacus leniusculus]
LVAN01076	38.27333	83.90667	1.132446	6.37E-05	Triple functional domain protein [Orchesella cincta]
MSTRG.797	60.35667	545.6333	3.176347	6.47E-05	uncharacterized protein LOC113805115 [Penaeus vannamei]
MSTRG.32324	34.45	94.56667	1.456828	6.62E-05	-
LVAN20788	44.01333	127.62	1.535842	7.00E-05	PREDICTED: matrix metalloproteinase-14-like isoform X1 [Hyalella azteca]
MSTRG.35340	2.613333	0.376667	-2.79453	7.42E-05	-
MSTRG.27803	4.206667	19.73667	2.230129	7.72E-05	waprin-Phi3-like [Penaeus vannamei]
LVAN24127	68.16333	154.4267	1.179854	8.03E-05	PREDICTED: alpha-tocopherol transfer protein-like isoform X5 [Hyalella azteca]

MSTRG.35599	100.9867	251.5633	1.316757	8.20E-05	-
MSTRG.33037	0.096667	4.23	5.451495	8.38E-05	uncharacterized protein LOC113824148 isoform X2 [Penaeus vannamei]
LVAN17866	34.33667	17.14	-1.00238	8.41E-05	PREDICTED: pteridine reductase 1-like [Branchiostoma belcheri]
LVAN04841	4.426667	1.486667	-1.57414	8.41E-05	PREDICTED: phosphatidylinositol phosphatase PTPRQ-like [Hyalella azteca]
LVAN05790	0.36	3.593333	3.319254	9.57E-05	innexin 7 [Homarus americanus]
LVAN09046	0.556667	6.823333	3.615591	9.64E-05	PREDICTED: uncharacterized protein LOC108674387 [Hyalella azteca]
LVAN10069	0.043333	0.996667	4.523562	0.000106	PREDICTED: fibrocystin-L-like [Saccoglossus kowalevskii]
MSTRG.36148	0.983333	7.656667	2.960964	0.000106	-
MSTRG.35456	11.55	31.76333	1.459469	0.00011	LOW QUALITY PROTEIN: UNC93-like protein, partial [Penaeus vannamei]
LVAN19677	63.21667	221.5333	1.809147	0.000112	PREDICTED: fatty-acid amide hydrolase 2-like [Parasteatoda tepidariorum]
LVAN14784	0.43	1.71	1.991588	0.000112	sodium leak channel non-selective [Cancer borealis]
LVAN21094	17.51333	39.85	1.186126	0.000112	PREDICTED: tyrosine-protein phosphatase non-receptor type 13-like isoform X3 [Hyalella azteca]
MSTRG.11233	131.0833	286.6533	1.128823	0.000112	leucine-rich repeat extensin-like protein 3 [Penaeus vannamei]
MSTRG.32691	8.596667	4	-1.10378	0.000115	partitioning defective 3 homolog B-like isoform X2 [Gadus morhua]
LVAN21505	72.42667	178.9367	1.304856	0.000116	PREDICTED: protein OS-9-like [Hyalella azteca]
MSTRG.32641	25.89667	10.83	-1.25773	0.000119	TRPM8 channel-associated factor 3-like [Penaeus vannamei]
MSTRG.131	8.303333	46.16	2.474881	0.00012	anti-lipopolysaccharide factor-like, partial [Penaeus vannamei]
LVAN24338	4.4	12.18	1.468939	0.000123	phosphoenolpyruvate carboxykinase [Litopenaeus vannamei]
LVAN07490	15.21333	5.803333	-1.39038	0.000125	PREDICTED: ethanolaminephosphotransferase 1-like [Hyalella azteca]
LVAN13516	2.89	10.50333	1.861706	0.000128	Retrovirus-related Pol polyprotein from transposon [Daphnia magna]
LVAN14104	6.826667	19.67	1.526744	0.000128	PREDICTED: unconventional myosin-Ie-like [Parasteatoda tepidariorum]
LVAN18391	36.81	82.49333	1.16418	0.000131	Galactosyl T domain containing protein [Trichuris trichiura]
LVAN08098	47.76667	97.53	1.029842	0.000133	vascular endothelial growth factor receptor precursor [Litopenaeus vannamei]
MSTRG.36992	9.98	4.163333	-1.2613	0.000135	solute carrier family 22 member 6-A-like [Penaeus vannamei]
LVAN10286	1.31	5.066667	1.95147	0.000136	PREDICTED: phosphoribosylformylglycinamidine synthase [Trichogramma pretiosum]

LVAN05330	0.626667	3.456667	2.463611	0.000138	PREDICTED: sodium-coupled monocarboxylate transporter 1-like [Hyalella azteca]
LVAN04363	4.9	12.53333	1.354917	0.000145	PREDICTED: prominin-1-like [Hyalella azteca]
LVAN11904	14.26667	44.39333	1.637695	0.000161	PREDICTED: 2-hydroxyacylsphingosine 1-beta-galactosyltransferase-like [Hyalella azteca]
MSTRG.22281	2.036667	10.25333	2.331811	0.000165	T-complex protein 11-like protein 1 isoform X1 [Penaeus vannamei]
LVAN22244	8.673333	18.00333	1.053606	0.000175	PREDICTED: serine/threonine-protein kinase Genghis Khan-like, partial [Hyalella azteca]
MSTRG.8295	0.69	5.206667	2.915692	0.000178	-
MSTRG.15608	15.16	33.64	1.149908	0.000196	-
MSTRG.35147	11.83333	38.80333	1.713324	0.000196	GRIP and coiled-coil domain-containing protein 2-like [Penaeus vannamei]
LVAN19319	0.92	5.393333	2.551471	0.000208	PREDICTED: uncharacterized protein LOC108670006 isoform X2 [Hyalella azteca]
LVAN05181	73.64	31.25333	-1.23648	0.000209	glycosyl-phosphatidylinositol-linked carbonic anhydrase [Litopenaeus vannamei]
LVAN08077	12.05333	31.41667	1.382098	0.000218	-
MSTRG.35256	47.89667	120.9133	1.335976	0.00022	uncharacterized protein LOC113825948 [Penaeus vannamei]
LVAN01963	3.683333	15.50667	2.073805	0.000221	PREDICTED: protein THEM6-like [Hyalella azteca]
LVAN19811	13.57	30.37333	1.162385	0.000227	PREDICTED: catenin alpha-like isoform X1 [Hyalella azteca]
LVAN11202	0.696667	3.53	2.341128	0.000237	PREDICTED: inositol hexakisphosphate and diphosphoinositol-pentakisphosphate kinase-like [Limulus polyphemus]
MSTRG.28257	42.99667	150.67	1.809095	0.000238	uncharacterized protein LOC113820575 [Penaeus vannamei]
LVAN23474	15.99667	35.92	1.167016	0.000243	PREDICTED: protein bark beetle-like [Hyalella azteca]
LVAN10004	0.926667	7.036667	2.92477	0.000266	PREDICTED: glucose-induced degradation protein 4 homolog [Parasteatoda tepidariorum]
LVAN15289	11.83333	5.476667	-1.11149	0.000269	Carbohydrate sulfotransferase 11, partial [Zootermopsis nevadensis]
LVAN18400	32.52333	14.27333	-1.18815	0.000269	PREDICTED: retinol dehydrogenase 12-like [Hyalella azteca]
LVAN00996	125.2233	280.2	1.161954	0.000272	beta-thymosin 3 [Pacifastacus leniusculus]
LVAN03636	17.76	44.67	1.330675	0.000274	PREDICTED: ATP-binding cassette sub-family A member 1-like [Hyalella azteca]
LVAN06273	32.01333	73.54	1.199856	0.000284	PREDICTED: dystonin-like isoform X2 [Hyalella azteca]

LVAN10542	22.24667	52.01667	1.225385	0.000288	PREDICTED: la-related protein 6-like [Hyalella azteca]
LVAN04066	9.55	22.53667	1.238702	0.000303	PREDICTED: ubiquitin thioesterase otulin-like [Saccoglossus kowalevskii]
LVAN17703	0.001	2.416667	11.2388	0.000322	triosephosphate isomerase [Penaeus monodon]
LVAN00448	0.663333	6.436667	3.278508	0.000322	PREDICTED: uncharacterized protein LOC108677823 isoform X2 [Hyalella azteca]
LVAN18667	53.26667	138.5533	1.379137	0.000346	AAEL011242-PA [Aedes aegypti]
LVAN13894	2.716667	7.926667	1.544877	0.000351	PREDICTED: plexin A3-like isoform X2 [Hyalella azteca]
MSTRG.5922	75.96	27.60333	-1.4604	0.000368	-
LVAN21082	7.73	25.91	1.744969	0.000368	PREDICTED: integrin alpha-PS5-like [Cephus cinctus]
LVAN16696	24.66	61.46	1.317475	0.000375	PREDICTED: phospholipid scramblase 1 isoform X1 [Bactrocera oleae]
LVAN17745	11.46333	5.3	-1.11296	0.000392	PREDICTED: solute carrier organic anion transporter family member 1C1-like [Hyalella azteca]
MSTRG.10970	7.51	21.49333	1.517004	0.000392	cinnamoyl-CoA reductase 2 [Parasteatoda tepidariorum]
LVAN16792	29.17667	9.45	-1.62643	0.000393	RFS2 protein [Daphnia magna]
LVAN06634	14.82333	64.68667	2.125598	0.000397	-
MSTRG.12363	0.001	17.98	14.13411	0.000402	-
LVAN07038	25.24333	12.48667	-1.01551	0.000402	GM17735 [Drosophila sechellia]
LVAN22614	0.393333	8.79	4.482039	0.00041	crustacean hyperglycemic hormone [Litopenaeus vannamei]
MSTRG.35769	3.21	8.63	1.426787	0.000417	exonuclease mut-7 homolog [Penaeus vannamei]
LVAN10725	19.04	6.14	-1.63272	0.000432	PREDICTED: sialin [Fopius arisanus]
LVAN17128	29.47	10.01333	-1.55732	0.000432	PREDICTED: carotenoid isomerooxygenase-like [Hyalella azteca]
LVAN07258	10.51333	25.14	1.257764	0.000434	-
LVAN05326	7.733333	30.75	1.991424	0.000435	PREDICTED: probable E3 ubiquitin-protein ligase DTX2 [Eurypyga helias]
LVAN21551	26.11333	57.9	1.148777	0.000437	PREDICTED: uncharacterized protein LOC108682637 [Hyalella azteca]
LVAN06014	7.58	20.33	1.42334	0.00046	Phospholipid-transporting ATPase IC [Daphnia magna]
LVAN23615	2.956667	8.976667	1.602208	0.000461	PREDICTED: E3 ubiquitin-protein ligase TRIM9 isoform X2 [Bemisia tabaci]
LVAN13669	136.23	274.5833	1.0112	0.000479	PREDICTED: innexin inx2-like isoform X2 [Hyalella azteca]

MSTRG.36758	15.23667	5.266667	-1.53259	0.0005	uncharacterized protein LOC113827127 [Penaeus vannamei]
LVAN22918	81.02333	35.75	-1.18039	0.000506	PREDICTED: TRPM8 channel-associated factor 2-like [Pelodiscus sinensis]
LVAN12902	9.796667	24.44	1.318881	0.000508	PREDICTED: tyrosine-protein phosphatase non-receptor type 14 [Galendromus occidentalis]
LVAN22777	2770.923	409.4733	-2.75853	0.000512	hemocyanin subunit L2, partial [Litopenaeus vannamei]
MSTRG.4837	51.59333	23.71	-1.12169	0.000521	-
LVAN00639	12.32333	157.8767	3.679334	0.000523	PREDICTED: sodium-dependent nutrient amino acid transporter 1-like [Hyalella azteca]
MSTRG.30547	0.746667	8.87	3.570398	0.000534	-
LVAN23225	26.33	52.75	1.002464	0.000537	PREDICTED: septin-7-like isoform X3 [Hyalella azteca]
MSTRG.6507	1.15	103.7533	6.49538	0.000541	caspase [Penaeus vannamei]
LVAN06612	0.186667	1.343333	2.847281	0.000585	PREDICTED: uncharacterized protein LOC108671097 [Hyalella azteca]
LVAN21713	14.71333	53.63333	1.866006	0.000596	PREDICTED: bifunctional glutamate/proline--tRNA ligase-like [Hyalella azteca]
LVAN06114	4.273333	9.74	1.18856	0.000596	domeless [Litopenaeus vannamei]
LVAN05927	18.24667	41.70333	1.19253	0.000597	PREDICTED: protein phosphatase 1E [Gekko japonicus]
MSTRG.31727	153.5633	371.5067	1.274554	0.000604	interferon regulatory factor [Penaeus vannamei]
LVAN19661	27.28667	57.68667	1.080042	0.000609	PREDICTED: multiple epidermal growth factor-like domains protein 10 [Polistes canadensis]
LVAN11952	0.24	3.33	3.794416	0.000647	-
LVAN17770	0.283333	1.37	2.273604	0.000672	AGAP006052-PA [Anopheles gambiae str. PEST] [Anopheles gambiae]
LVAN22660	3.823333	9.923333	1.375994	0.000694	Low-density lipoprotein receptor-related protein 2 [Daphnia magna]
LVAN01799	39.65333	99.93	1.333476	0.000712	-
MSTRG.34031	2.216667	6.3	1.50696	0.000719	-
LVAN05324	99.99	49.17333	-1.02391	0.000719	4-coumarate--CoA ligase [Daphnia magna]
LVAN01695	67.12667	30.88	-1.12021	0.000755	-
LVAN10712	6.533333	13.31	1.026619	0.000757	PREDICTED: arf-GAP with SH3 domain, ANK repeat and PH domain-containing protein 2-like isoform X2 [Hyalella azteca]

LVAN11905	12.46667	41.07	1.720009	0.000767	PREDICTED: 2-hydroxyacylsphingosine 1-beta-galactosyltransferase-like [Hyalella azteca]
LVAN13473	1391.233	193.09	-2.84902	0.000781	hemocyanin subunit L2, partial [Litopenaeus vannamei]
LVAN06015	9.343333	23.27333	1.316669	0.000793	Phospholipid-transporting ATPase [Daphnia magna]
LVAN09949	19.76333	8.103333	-1.28624	0.000794	PREDICTED: proton-coupled folate transporter-like [Agrilus planipennis]
LVAN08646	1.236667	38.24	4.950554	0.000805	caspase [Eriocheir sinensis]
MSTRG.43454	15.71333	40.13333	1.352812	0.000841	-
LVAN10133	0.001	0.81	9.661778	0.000848	PREDICTED: butyrophilin subfamily 1 member A1-like isoform X3 [Stegastes partitus]
LVAN10126	3.493333	8.113333	1.21569	0.000856	AGAP004186-PA [Anopheles gambiae str. PEST] [Anopheles gambiae]
LVAN02946	0.36	3.766667	3.38722	0.000873	antilipopolysaccharide factor isoform 5 [Fenneropenaeus chinensis]
LVAN09419	14.24	6.576667	-1.11452	0.000875	PREDICTED: 2-acylglycerol O-acyltransferase 1-like [Hyalella azteca]
LVAN10922	1.486667	6.273333	2.077151	0.000895	Integrin alpha-6 [Daphnia magna]
LVAN08175	0.001	1.596667	10.64085	0.000951	transcription factor ATF-b [Litopenaeus vannamei]
LVAN22923	23.55	10.79333	-1.12559	0.000974	PREDICTED: protein FAM115-like [Oryzias latipes]
LVAN08647	1.193333	44.11667	5.208255	0.001	caspase [Eriocheir sinensis]
LVAN19962	5.186667	13.22333	1.350206	0.001004	integrin beta subunit [Litopenaeus vannamei]
LVAN22776	3012.777	475.6033	-2.66326	0.001042	hemocyanin V4 [Litopenaeus vannamei]
LVAN07579	2.643333	7.006667	1.40637	0.001057	Serine/threonine-protein kinase unc-51 [Zootermopsis nevadensis]
MSTRG.34662	28.27	56.96667	1.010846	0.001091	PREDICTED: G-protein coupled receptor Mth2-like [Hyalella azteca]
LVAN02518	4.446667	10.69333	1.265915	0.001094	PREDICTED: atypical protein kinase C-like isoform X5 [Hyalella azteca]
LVAN00449	344.1533	146.81	-1.2291	0.001101	PREDICTED: very low-density lipoprotein receptor isoform X2 [Microplitis demolitor]
MSTRG.264	28.04667	71.88	1.357761	0.00112	Enterin neuropeptide, partial [Heterocephalus glaber]
LVAN08651	1.506667	62.81333	5.381639	0.001138	caspase [Eriocheir sinensis]
LVAN12667	22.84333	10.23	-1.15897	0.001138	PREDICTED: flavin-containing monooxygenase FMO GS-OX-like 4 isoform X2 [Hyalella azteca]
LVAN22774	1110.9	235.5467	-2.23764	0.001143	hemocyanin subunit L2, partial [Litopenaeus vannamei]

LVAN15835	3.13	7.516667	1.26393	0.001143	PREDICTED: rho GTPase-activating protein 7-like [Hyalella azteca]
LVAN21385	2.933333	7.43	1.340821	0.001175	PREDICTED: tyrosine-protein phosphatase 99A-like isoform X2 [Hyalella azteca]
LVAN05145	39.69333	19.35667	-1.03607	0.001181	PREDICTED: UDP-glucuronosyltransferase 2B19-like isoform X1 [Hyalella azteca]
LVAN21736	48.31	110.58	1.194697	0.001202	PREDICTED: sequestosome-1 [Neodiprion lecontei]
MSTRG.41759	0.373333	2.953333	2.983808	0.001205	-
MSTRG.34661	11.66333	29.42	1.334817	0.001228	uncharacterized protein LOC113825457 [Penaeus vannamei]
LVAN02913	0.58	2.666667	2.200913	0.001263	PREDICTED: epithelial chloride channel protein-like [Limulus polyphemus]
LVAN20620	0.093333	4.183333	5.486117	0.001324	-
LVAN21550	21.51	61.57	1.51722	0.001328	PREDICTED: branched-chain-amino-acid aminotransferase, cytosolic-like [Hyalella azteca]
MSTRG.32250	12.03	26.70333	1.150383	0.00138	extended synaptotagmin-2-like [Penaeus vannamei]
MSTRG.22148	0.53	3.016667	2.508891	0.001402	single VWC domain protein 4 [Penaeus japonicus]
LVAN12926	0.216667	2.84	3.712342	0.001406	PREDICTED: cell wall protein DAN4-like, partial [Bactrocera oleae]
LVAN23848	0.001	0.953333	9.896837	0.00142	heat shock protein 21 [Macrobrachium rosenbergii]
LVAN21819	13.32667	4.86	-1.45529	0.001423	PREDICTED: peroxisomal sarcosine oxidase isoform X2 [Crassostrea gigas]
LVAN22782	25.47333	60.91	1.257691	0.001462	cactus protein [Litopenaeus vannamei]
LVAN05534	0.536667	3.366667	2.649223	0.00147	X-linked interleukin-1 receptor accessory protein-like 1 [Zootermopsis nevadensis]
LVAN10217	28.31667	61.58667	1.120967	0.001507	PREDICTED: GTP-binding protein 2-like [Hyalella azteca]
MSTRG.5921	173.6833	65.25667	-1.41226	0.001507	-
LVAN02406	11.58667	4.43	-1.38709	0.001515	PREDICTED: feline leukemia virus subgroup C receptor-related protein 2 [Zonotrichia albicollis]
LVAN09539	9.08	20.72333	1.190492	0.001569	PREDICTED: RING finger protein nhl-1-like [Limulus polyphemus]
LVAN00869	14.29	6.1	-1.22812	0.001575	PREDICTED: solute carrier family 22 member 3-like [Hyalella azteca]
LVAN15180	2.31	5.436667	1.23483	0.001592	PREDICTED: ADAMTS-like protein 1 [Hyalella azteca]
MSTRG.35928	0.04	1.37	5.098032	0.001608	Ankyrin-3 [Amphibalanus amphitrite]
MSTRG.33563	9.166667	22.60333	1.302066	0.001631	-

LVAN05662	0.053333	11.42333	7.74273	0.001636	Low choriolytic enzyme [Daphnia magna]
MSTRG.18187	21.73	51.43333	1.243015	0.001703	LOW QUALITY PROTEIN: transcription factor kayak-like [Penaeus vannamei]
MSTRG.20197	953.11	400.7	-1.25012	0.001705	-
LVAN19203	47.09667	19.19	-1.29527	0.001738	PREDICTED: phosphotriesterase-related protein-like [Hyalella azteca]
LVAN09040	0.001	1.223333	10.2566	0.001777	PREDICTED: flocculation protein FLO11-like, partial [Hyalella azteca]
LVAN18669	2244.083	1064.453	-1.07601	0.001789	PREDICTED: betaine--homocysteine S-methyltransferase 1-like [Hyalella azteca]
LVAN10131	0.12	1.373333	3.516576	0.001856	PREDICTED: uncharacterized protein LOC108682106 [Hyalella azteca]
LVAN25426	8.3	18.37667	1.146692	0.001866	PREDICTED: cAMP-specific 3',5'-cyclic phosphodiesterase 4C-like isoform X5 [Hyalella azteca]
LVAN24049	12.21	29.34333	1.26497	0.00189	PREDICTED: sialin-like isoform X3 [Hyalella azteca]
LVAN10355	2.343333	9.073333	1.95307	0.001992	PREDICTED: GTP cyclohydrolase 1-like [Hyalella azteca]
LVAN19595	0.746667	4.353333	2.543584	0.002024	PREDICTED: microtubule-associated protein futsch-like [Hyalella azteca]
LVAN16227	2.763333	7	1.340945	0.002041	PREDICTED: FERM domain-containing protein 4A-like isoform X2 [Lingula anatina]
LVAN06016	9.403333	24.17333	1.362172	0.002052	PREDICTED: probable phospholipid-transporting ATPase IF isoform X2 [Bactrocera cucurbitae]
MSTRG.1898	1.24	3.126667	1.334285	0.002068	-
LVAN22263	0.123333	2.006667	4.024166	0.002068	serine proteinase 1 [Portunus trituberculatus]
LVAN04834	76.08333	180.8433	1.249088	0.002164	-
MSTRG.32720	2.73	7.613333	1.479627	0.002164	chloride intracellular channel exl-1-like [Penaeus vannamei]
MSTRG.35344	3.226667	0.52	-2.63346	0.002166	-
LVAN06426	0.15	4.66	4.957296	0.002263	-
LVAN23875	0.001	0.413333	8.691162	0.002263	heat shock protein 21 [Macrobrachium rosenbergii]
MSTRG.35343	2.236667	0.283333	-2.98078	0.00231	-
LVAN17991	14.43333	37.86667	1.391524	0.002314	PREDICTED: uncharacterized protein LOC108682462 [Hyalella azteca]
LVAN00558	22.09	10.11333	-1.12713	0.002343	PREDICTED: xaa-Pro aminopeptidase 1-like [Hyalella azteca]
MSTRG.20198	703.09	283.8467	-1.3086	0.002368	-

LVAN04381	0.233333	1.906667	3.030588	0.002398	heat shock protein [Cherax destructor]
LVAN01773	6.91	14.14667	1.033705	0.002485	Dorsal [Marsupenaeus japonicus]
LVAN09530	11.82	3.906667	-1.59722	0.002495	PREDICTED: facilitated trehalose transporter Tret1 isoform X3 [Diachasma alloeum]
LVAN13031	0.72	5.706667	2.986579	0.002537	-
LVAN08650	0.473333	15.58667	5.041312	0.002608	caspase [Eriocheir sinensis]
LVAN11936	2.706667	14.46	2.417478	0.002705	crustacean hematopoietic factor-like protein [Litopenaeus vannamei]
LVAN22362	75.46667	36.14333	-1.06211	0.002705	PREDICTED: probable 4-coumarate--CoA ligase 3 [Hyalella azteca]
LVAN15818	1.263333	4.226667	1.742285	0.002709	PREDICTED: sterol O-acyltransferase 1-like [Hyalella azteca]
MSTRG.32741	0.25	5.233333	4.38773	0.002716	aromatic-L-amino-acid decarboxylase-like [Penaeus vannamei]
LVAN04905	40.43	16.56667	-1.28714	0.002718	Ectonucleotide pyrophosphatase/phosphodiesterase family member [Daphnia magna]
MSTRG.35844	339.6367	161.8033	-1.06975	0.002763	zinc proteinase [Astacus astacus]
LVAN15951	1.67	5.25	1.652469	0.002775	Rho GTPase-activating protein 20 [Zootermopsis nevadensis]
MSTRG.42024	9.103333	2.92	-1.64043	0.002814	-
LVAN05590	4.756667	14.38667	1.59671	0.00286	PREDICTED: uncharacterized protein LOC106460216 [Limulus polyphemus]
LVAN00089	126.6467	254.0267	1.004171	0.00286	Sodium/glucose cotransporter [Daphnia magna]
MSTRG.21060	9.143333	21.80333	1.253757	0.00302	-
LVAN04091	10.28667	26.96333	1.390223	0.003123	PREDICTED: N-acetylglucosamine-6-phosphate deacetylase [Cyphomyrmex costatus]
LVAN15255	0.536667	1.753333	1.708002	0.003183	PREDICTED: titin isoform X2 [Eufriesea mexicana]
LVAN23293	4.253333	10.35667	1.283894	0.003219	PREDICTED: transient receptor potential cation channel subfamily A member 1-like [Hyalella azteca]
LVAN14947	6.42	14.45333	1.170757	0.003227	PREDICTED: copine-8-like [Hyalella azteca]
LVAN09681	8.383333	17.11	1.029244	0.003232	PREDICTED: probable serine hydrolase [Hyalella azteca]
LVAN24209	16.42333	34.75333	1.081404	0.003246	PREDICTED: GRB2-associated-binding protein 2-like [Hyalella azteca]
LVAN21081	3.823333	10.27	1.425533	0.003285	PREDICTED: poly [ADP-ribose] polymerase 3-like [Hyalella azteca]
LVAN04845	2.256667	5.69	1.334235	0.003288	Helicase POLQ-like [Zootermopsis nevadensis]
LVAN06380	15.85667	31.92	1.009371	0.003288	cryptochrome [Euphausia superba]

LVAN15267	0.983333	2.88	1.550316	0.00329	lipoprotein receptor [Callinectes sapidus]
LVAN04024	0.773333	3.59	2.214822	0.00329	PREDICTED: tissue factor pathway inhibitor isoform X2 [Columba livia]
LVAN13110	33.48333	14.59	-1.19846	0.003306	C-type lectin [Penaeus monodon]
LVAN11053	2.75	61.50333	4.483161	0.003344	caspase [Eriocheir sinensis]
LVAN18585	11.16	4.686667	-1.2517	0.003369	PREDICTED: proton-coupled folate transporter-like, partial [Limulus polyphemus]
MSTRG.12364	0.053333	4.033333	6.240791	0.003467	-
LVAN21420	12.15	26.30667	1.114472	0.003479	PREDICTED: proton-coupled amino acid transporter 2-like [Hyalella azteca]
MSTRG.43249	4.156667	12.27	1.561636	0.003508	-
MSTRG.30304	40.3	125.0367	1.633499	0.003508	solute carrier family 15 member 2-like isoform X4 [Penaeus vannamei]
LVAN15196	0.076667	1.196667	3.964278	0.003523	PREDICTED: AT-rich interactive domain-containing protein 3C isoform X5 [Microplitis demolitor]
LVAN13412	239.2667	499.91	1.063049	0.003549	PREDICTED: leucine-rich repeat neuronal protein 1-like [Hyalella azteca]
LVAN23871	0.013333	1.04	6.285402	0.003558	heat shock protein 21 [Macrobrachium rosenbergii]
LVAN05137	153.0967	466.0333	1.60599	0.003587	-
LVAN12309	4.98	10.62667	1.093471	0.003724	CLUMA_CG018239, isoform A [Clunio marinus]
LVAN20818	28.94	70.00667	1.274427	0.003731	PREDICTED: CCAAT/enhancer-binding protein epsilon-like [Hyalella azteca]
MSTRG.31536	0.836667	3.73	2.156451	0.003739	uncharacterized protein LOC113823067 [Penaeus vannamei]
LVAN11900	16.95333	37.14333	1.131534	0.00379	PREDICTED: C-Maf-inducing protein-like [Lingula anatina]
LVAN04486	5.013333	10.99	1.132349	0.003839	PREDICTED: rap guanine nucleotide exchange factor 2-like [Hyalella azteca]
MSTRG.36990	1.696667	0.626667	-1.43693	0.003849	solute carrier family 22 member 6-A-like [Penaeus vannamei]
LVAN24649	0.543333	2.056667	1.920399	0.003998	G-protein coupled receptor 64 [Zootermopsis nevadensis]
LVAN19591	0.25	1.466667	2.552541	0.004131	PREDICTED: microtubule-associated protein futsch-like [Hyalella azteca]
MSTRG.1238	3.83	10.68	1.479495	0.004166	-
LVAN18819	0.513333	3.586667	2.804676	0.00424	serine protease [Scylla paramamosain]
LVAN06799	1.223333	4.05	1.727104	0.004289	PREDICTED: uncharacterized protein LOC108676555 [Hyalella azteca]
MSTRG.17690	9.11	20.06667	1.139278	0.004302	-

LVAN09185	1.496667	6.126667	2.033349	0.004376	PREDICTED: multidrug resistance-associated protein 1 isoform X7 [Trichogramma pretiosum]
MSTRG.33550	5.14	15.20667	1.564864	0.004481	glycine N-acyltransferase-like protein 3 isoform X2 [Pelodiscus sinensis]
LVAN04130	7.143333	17.77	1.314774	0.004554	acetyl-CoA carboxylase [Macrobrachium nipponense]
LVAN24260	2.193333	11.92667	2.442994	0.004554	serine proteinase inhibitor B3 [Penaeus monodon]
MSTRG.22705	14.23333	29.41333	1.047197	0.004554	transient receptor potential cation channel subfamily A member 1 homolog, partial [Penaeus vannamei]
LVAN21954	146.8867	456.8133	1.636901	0.004562	PREDICTED: solute carrier family 15 member 2-like isoform X3 [Hyalella azteca]
LVAN01305	1.546667	4.086667	1.401762	0.004594	PREDICTED: uncharacterized protein LOC108674544 [Hyalella azteca]
LVAN08515	2.51	7.3	1.540209	0.004634	RecName: Full=Crustacean calcium-binding protein 23; Short=CCBP-23
MSTRG.32396	56.19333	129.5567	1.205112	0.004634	-
MSTRG.32534	0.47	2.79	2.569532	0.004636	uncharacterized protein LOC113823796 [Penaeus vannamei]
LVAN24191	2.39	0.213333	-3.48583	0.004636	PREDICTED: gamma-butyrobetaine dioxygenase-like isoform X2 [Lingula anatina]
LVAN17668	16.16667	34.48	1.092738	0.004671	Le1-cadherin [Ligia exotica]
LVAN19831	1.92	4.663333	1.280255	0.004894	PREDICTED: SLIT-ROBO Rho GTPase-activating protein 1-like [Hyalella azteca]
LVAN23334	8.383333	22.36667	1.415754	0.004894	PREDICTED: beta-ureidopropionase-like [Priapulus caudatus]
LVAN09475	0.673333	1.876667	1.47878	0.004908	PREDICTED: myosin-I heavy chain-like isoform X2 [Bombus impatiens]
LVAN14367	0.001	2.116667	11.04758	0.004954	-
LVAN09584	744.77	1635.277	1.134668	0.004956	C-type lectin [Litopenaeus vannamei]
LVAN25399	317.5733	26.17333	-3.60092	0.005106	PREDICTED: gamma-butyrobetaine dioxygenase-like [Hyalella azteca]
LVAN13136	2.396667	8.04	1.746166	0.005112	PREDICTED: uncharacterized protein LOC108674705 [Hyalella azteca]
LVAN25356	20.18333	7.46	-1.43592	0.005183	hemocyanin subunit 1 [Procambarus clarkii]
LVAN10231	4.163333	15.5	1.896457	0.005183	PREDICTED: UDP-glucuronosyltransferase 2B14-like [Hyalella azteca]
LVAN24197	0.926667	4.153333	2.164147	0.005183	-
LVAN11363	19.90667	42.53333	1.095342	0.005243	PREDICTED: cytohesin-1-like isoform X3 [Hyalella azteca]
MSTRG.25451	7.726667	2.65	-1.54385	0.005243	uncharacterized protein LOC113818508 [Penaeus vannamei]

LVAN06271	3.003333	9.983333	1.732957	0.00531	PREDICTED: plectin-like [Papilio polytes]
MSTRG.14924	3.293333	8.96	1.44395	0.005317	epsin-2-like isoform X2 [Penaeus vannamei]
LVAN22058	2.723333	8.403333	1.625588	0.005378	PREDICTED: hexosaminidase D-like [Hyalella azteca]
LVAN10356	1.206667	5.046667	2.064304	0.005465	PREDICTED: GTP cyclohydrolase 1-like [Hyalella azteca]
MSTRG.12365	0.203333	12.77	5.972768	0.005468	-
MSTRG.13652	2.036667	0.856667	-1.2494	0.005549	tetraspanin-2A-like isoform X1 [Penaeus vannamei]
MSTRG.10312	0.25	3.86	3.948601	0.005549	uncharacterized protein LOC113807085 [Penaeus vannamei]
MSTRG.20212	37.33667	82.37333	1.141584	0.005549	-
LVAN15253	1.506667	3.65	1.276536	0.005767	PREDICTED: titin isoform X2 [Eufriesea mexicana]
LVAN22073	1.383333	4.673333	1.756303	0.005784	-
LVAN18057	0.976667	3.646667	1.90064	0.005817	PREDICTED: protein ariadne-1 isoform X2 [Cimex lectularius]
LVAN21173	1.59	5.45	1.777229	0.005888	Group 3 secretory phospholipase A2 [Daphnia magna]
LVAN12011	4.503333	1.966667	-1.19524	0.006036	scavenger receptor class B, croquemort type [Marsupenaeus japonicus]
LVAN12738	9.98	3.63	-1.45907	0.006052	fibrinogen-like protein [Fenneropenaeus merguiensis]
LVAN01421	0.24	2.226667	3.213779	0.00607	serine proteinase [Litopenaeus vannamei]
LVAN05432	11.94667	30.68	1.36069	0.00608	Lactosylceramide [Daphnia magna]
MSTRG.193	2.97	6.55	1.141032	0.00608	Vps52 [Trinorchestia longiramus]
LVAN23105	5.743333	14.83667	1.369207	0.006148	PREDICTED: innexin inx2-like [Hyalella azteca]
LVAN18521	38.02	15.30667	-1.3126	0.00617	chitinase 3 precursor, partial [Penaeus monodon]
LVAN08679	0.106667	0.956667	3.164907	0.006219	PREDICTED: neurotrypsin, partial [Tinamus guttatus]
LVAN01206	0.173333	13.13333	6.24354	0.006226	prophenoloxidase activating enzyme 2 [Litopenaeus vannamei]
LVAN20650	7.556667	3.5	-1.1104	0.006226	Galactoside 2-alpha-L-fucosyltransferase 2 [Daphnia magna]
LVAN19878	0.263333	1.79	2.764998	0.006374	juvenile hormone esterase-like carboxylesterase 1 [Eriocheir sinensis]
LVAN16375	0.066667	6.1	6.5157	0.006374	PREDICTED: methyltransferase-like protein 24 [Biomphalaria glabrata]
LVAN22149	0.346667	1.713333	2.305185	0.006457	PREDICTED: protein unc-13 homolog D isoform X1 [Cimex lectularius]
LVAN21560	5.08	11.64	1.196191	0.006521	PREDICTED: serine/threonine-protein kinase PLK1-like [Hyalella azteca]

MSTRG.28462	8.336667	1.9	-2.13347	0.006521	Ectoine hydroxylase, partial [Penaeus vannamei]
LVAN24930	10.42	25.88667	1.312854	0.006521	prophenoloxidase-1 [Litopenaeus vannamei]
LVAN09516	13.24	29.85667	1.17315	0.006521	PREDICTED: probable cytochrome P450 49a1 [Hyalella azteca]
MSTRG.32325	0.126667	1.55	3.613159	0.006521	uncharacterized protein LOC113823637 [Penaeus vannamei]
LVAN05329	1.616667	6.67	2.044665	0.006593	PREDICTED: sodium-coupled monocarboxylate transporter 1-like [Hyalella azteca]
LVAN15924	2	6.543333	1.710026	0.006664	PREDICTED: poly [ADP-ribose] polymerase 3 [Apteryx australis mantelli]
LVAN20480	3.573333	1.37	-1.38309	0.006664	PREDICTED: GRAM domain-containing protein 1B-like isoform X1 [Acromyrmex echinatior]
MSTRG.27179	0.086667	2.586667	4.899473	0.006927	-
LVAN05215	5.853333	13.57667	1.213799	0.00693	PREDICTED: titin-like [Nicrophorus vespilloides]
MSTRG.8094	0.51	2.886667	2.500835	0.006961	uncharacterized protein LOC113824484 [Penaeus vannamei]
LVAN17949	0.153333	5.85	5.253693	0.006971	PREDICTED: serine/threonine-protein kinase SBK1-like [Hyalella azteca]
LVAN21088	1.776667	5.25	1.563144	0.007167	PREDICTED: poly [ADP-ribose] polymerase 3-like [Hyalella azteca]
LVAN03448	19.84333	40.34667	1.023795	0.007181	PREDICTED: dehydrodolichyl diphosphate synthase complex subunit DHDDS-like [Hyalella azteca]
LVAN07945	30.70333	63.11333	1.03955	0.007635	Ras superfamily small GTPase Rab8 [Nilaparvata lugens]
LVAN16084	1.856667	17.13	3.205738	0.007672	antimicrobial peptide type 1 precursor Ic [Pandalopsis japonica]
LVAN24131	2.803333	10.16333	1.858158	0.007696	PREDICTED: mannosylglucosyl-3-phosphoglycerate phosphatase-like isoform X2 [Hyalella azteca]
LVAN03812	0.001	8.61	13.0718	0.007755	triosephosphate isomerase [Penaeus monodon]
LVAN11635	2.596667	0.56	-2.21316	0.007864	PREDICTED: chlorophyllase-1, chloroplastic-like [Hyalella azteca]
LVAN08211	5.343333	11.37667	1.090266	0.007896	PREDICTED: formin-like protein CG32138 isoform X1 [Trachymyrmex cornetzi]
LVAN00622	7.036667	16.25667	1.208067	0.008116	PREDICTED: multifunctional protein ADE2 [Crassostrea gigas]
LVAN07315	7.383333	3.26	-1.1794	0.008197	PREDICTED: heparan-alpha-glucosaminide N-acetyltransferase-like [Hyalella azteca]
LVAN14730	9.653333	4.366667	-1.14449	0.008274	PREDICTED: organic cation transporter-like protein isoform X2 [Hyalella azteca]
MSTRG.34666	0.23	2.126667	3.208888	0.008274	probable G-protein coupled receptor Mth-like 1 [Penaeus vannamei]

LVAN22789	5.943333	14.41	1.277726	0.008277	PREDICTED: serine/threonine-protein phosphatase 6 regulatory ankyrin repeat subunit A-like, partial [Amphimedon queenslandica]
MSTRG.27738	4.883333	13.65	1.482963	0.008548	Histone deacetylase 4 [Melipona quadrifasciata]
MSTRG.37549	1.166667	3.893333	1.738613	0.00864	protein bark beetle-like isoform X1 [Penaeus vannamei]
LVAN03810	0.16	12.05	6.234817	0.008699	triosephosphate isomerase [Penaeus monodon]
LVAN24489	1.25	4.306667	1.784644	0.009005	caspase 4 [Litopenaeus vannamei]
LVAN03817	0.001	5.153333	12.33129	0.009137	triosephosphate isomerase [Penaeus monodon]
MSTRG.28446	6.4	16.67333	1.381399	0.009145	poly [ADP-ribose] polymerase 3-like [Penaeus vannamei]
LVAN03231	0.066667	2.84	5.412782	0.009159	PREDICTED: G-protein coupled receptor Mth2 [Acyrthosiphon pisum]
LVAN15599	8.396667	3.693333	-1.18489	0.009166	PREDICTED: protein AMN1 homolog [Crassostrea gigas]
MSTRG.28647	7.116667	18.87667	1.40733	0.009249	group 3 secretory phospholipase A2-like [Penaeus vannamei]
LVAN03729	6.723333	15.03333	1.160916	0.009338	PREDICTED: wee1-like protein kinase isoform X1 [Bombus terrestris]
LVAN14424	0.43	1.98	2.203092	0.009363	PREDICTED: protein trapped in endoderm-1-like isoform X2 [Hyalella azteca]
LVAN04829	4.703333	12.18333	1.373153	0.009382	PREDICTED: bcl-2-like protein 1 [Hyalella azteca]
MSTRG.28893	2.356667	6.69	1.505258	0.009405	hypothetical protein Avbf_06200 [Armadillidium vulgare]
MSTRG.32090	5.553333	11.55667	1.057299	0.009492	Transcription initiation factor TFIID subunit 4 [Penaeus vannamei]
LVAN10880	0.83	2.833333	1.771317	0.009595	Retrovirus-related Pol polyprotein from transposon 412-like Protein [Tribolium castaneum]
LVAN00452	5.69	2.383333	-1.25545	0.009655	-
LVAN08648	0.026667	4.013333	7.23362	0.009655	caspase [Eriocheir sinensis]
LVAN23488	2.09	13.42	2.68281	0.009707	crustacyanin A, partial [Penaeus monodon]
MSTRG.19665	1.793333	6.93	1.950212	0.009726	dual specificity protein phosphatase 10-like [Penaeus vannamei]
LVAN15469	0.323333	3.756667	3.538359	0.00976	-
LVAN16090	14.42667	6.293333	-1.19684	0.009938	Nose resistant to fluoxetine protein [Daphnia magna]
LVAN03480	0.163333	1.396667	3.096097	0.010057	PREDICTED: mucin-5AC [Fukomys damarensis]
LVAN11428	22.24667	55.59	1.321236	0.010109	pacifastin heavy chain precursor [Pacifastacus leniusculus]

LVAN02959	0.963333	2.453333	1.348636	0.010212	PREDICTED: ankyrin repeat and fibronectin type-III domain-containing protein 1-like isoform X2 [Hyalella azteca]
LVAN20947	3.016667	22.15333	2.876497	0.010219	PREDICTED: keratin, type I cytoskeletal 9-like [Hyalella azteca]
MSTRG.31370	0.576667	1.983333	1.782118	0.010373	kielin/chordin-like protein isoform X1 [Limulus polyphemus]
MSTRG.36710	0.69	4.856667	2.815298	0.010613	uncharacterized protein LOC113827091 [Penaeus vannamei]
LVAN02566	3.26	9.033333	1.470386	0.010613	PREDICTED: innexin inx2-like [Hyalella azteca]
LVAN23845	0.056667	1.016667	4.165203	0.010613	heat shock protein 21 [Macrobrachium rosenbergii]
MSTRG.22628	3.43	8.19	1.255655	0.010654	ctenidin-3-like [Penaeus vannamei]
LVAN06305	8.07	19.51667	1.274066	0.010805	cytochrome P450 CYP2 [Eriocheir sinensis]
LVAN11181	0.583333	0.08	-2.86625	0.010818	PREDICTED: alpha-mannosidase 2C1-like [Hyalella azteca]
LVAN03353	19.54667	7.72	-1.34025	0.0109	Glucose-6-phosphate translocase, partial [Stegodyphus mimosarum]
MSTRG.21114	0.316667	1.373333	2.116645	0.010937	-
LVAN16857	3.7	10.35	1.484034	0.011142	sodium-calcium exchanger 1 [Cherax cainii]
LVAN15807	0.71	0.26	-1.44931	0.011166	PREDICTED: flocculation protein FLO11-like [Hyalella azteca]
LVAN04380	0.001	0.483333	8.916875	0.011313	heat shock protein 70C1 [Thysanoessa inermis]
LVAN04715	24.07667	65.48	1.443419	0.011518	PREDICTED: transcriptional regulator ERG isoform X5 [Bos taurus]
LVAN17699	1.2	0.17	-2.81943	0.011568	PREDICTED: arf-GAP with SH3 domain, ANK repeat and PH domain-containing protein 1 isoform X4 [Cerapachys biroi]
MSTRG.20057	11.82333	34.61333	1.549691	0.011681	hypothetical protein C7M84_011991 [Penaeus vannamei]
LVAN16507	0.673333	2.613333	1.956498	0.011847	PREDICTED: platelet binding protein GspB-like [Xenopus tropicalis]
LVAN09250	9.47	23.45667	1.308562	0.01203	Abhydrolase domain-containing protein 10, mitochondrial [Lepeophtheirus salmonis]
LVAN23771	47.93333	127.3733	1.409962	0.01203	PREDICTED: 6-phosphofructo-2-kinase/fructose-2,6-bisphosphatase isoform X2 [Bemisia tabaci]
LVAN00436	4.83	1.28	-1.91588	0.012079	PREDICTED: proton-coupled folate transporter-like [Hyalella azteca]
LVAN00920	8.78	21.62667	1.300518	0.012115	PREDICTED: glucose-6-phosphate 1-dehydrogenase-like isoform X1 [Hyalella azteca]
LVAN10453	0.19	3.263333	4.102275	0.012169	-

LVAN02871	12.71	5.463333	-1.21811	0.012169	PREDICTED: organic cation transporter protein-like [Limulus polyphemus]
LVAN15854	7.436667	17.86	1.264004	0.012169	PREDICTED: epsin-1-like isoform X4 [Aedes albopictus]
MSTRG.92	0.35	2.64	2.915111	0.012356	-
LVAN22814	21.50667	46.50333	1.11255	0.012356	Putative proline-rich protein 21 [Myotis davidii]
MSTRG.35455	3.933333	10.56	1.424785	0.012544	-
MSTRG.15279	0.153333	1.303333	3.087463	0.013034	-
LVAN12708	11.21667	44.39	1.984591	0.013034	-
LVAN08569	0.84	3.003333	1.838103	0.013041	PREDICTED: speedy protein C [Xenopus tropicalis]
LVAN04306	19.03667	40.45	1.087359	0.013064	PREDICTED: bifunctional purine biosynthesis protein PURH [Oryctolagus cuniculus]
LVAN10787	6.7	3.086667	-1.11811	0.013064	PREDICTED: short-chain dehydrogenase/reductase family 16C member 6 [Solenopsis invicta]
MSTRG.27811	0.306667	2.386667	2.960254	0.013163	-
MSTRG.21921	0.526667	4.583333	3.121435	0.013276	H/ACA ribonucleoprotein complex subunit gar1-like [Penaeus vannamei]
LVAN06009	2.87	7.96	1.471718	0.013357	PREDICTED: WD repeat-containing protein 47 isoform X3 [Agrilus planipennis]
LVAN05504	12.08333	24.45667	1.017209	0.013433	PREDICTED: uncharacterized protein LOC658443 isoform X2 [Tribolium castaneum]
LVAN10953	0.076667	0.756667	3.302987	0.013581	PREDICTED: protein Wnt-8b-like [Limulus polyphemus]
MSTRG.20199	77	34.52	-1.15743	0.013608	-
LVAN23388	15.44667	31.39333	1.023163	0.014073	PREDICTED: ets DNA-binding protein pokkuri isoform X4 [Polistes dominula]
LVAN10611	8.35	24.71667	1.565636	0.014073	PREDICTED: LOW QUALITY PROTEIN: CD109 antigen-like [Galendromus occidentalis]
LVAN06584	23.45333	47.04	1.004095	0.014287	PREDICTED: serine-rich adhesin for platelets-like [Hyalella azteca]
LVAN23843	0.001	0.426667	8.736966	0.01444	heat shock protein 21 [Macrobrachium rosenbergii]
MSTRG.16661	2.123333	4.67	1.137092	0.014556	zinc finger SWIM domain-containing protein 5-like [Penaeus vannamei]
LVAN07297	6.143333	2.503333	-1.29517	0.014572	PREDICTED: glycine N-acyltransferase-like protein 3 isoform X2 [Cyprinodon variegatus]
LVAN14946	8.916667	19.27333	1.11203	0.014572	PREDICTED: copine-8-like [Hyalella azteca]

LVAN06850	1.35	4.403333	1.705637	0.014682	-
LVAN10070	0.073333	0.89	3.601264	0.014798	PREDICTED: fibrocystin-L-like [Xenopus laevis]
MSTRG.35981	3.363333	1.363333	-1.30275	0.014829	keratin, type I cytoskeletal 9-like [Penaeus vannamei]
LVAN11316	1.566667	4.69	1.58189	0.015159	PREDICTED: mediator of RNA polymerase II transcription subunit 15-like isoform X3 [Hyalella azteca]
LVAN16892	4.023333	8.673333	1.108195	0.015185	PREDICTED: exportin-4-like [Hyalella azteca]
LVAN09991	76.63	157.0267	1.035028	0.015247	heat shock protein 90 [Palaemon carinicauda]
LVAN18385	2.333333	5.48	1.231783	0.015401	-
MSTRG.41129	0.306667	2.973333	3.277338	0.016849	hypothetical protein C7M84_023505 [Penaeus vannamei]
LVAN24031	0.363333	3.363333	3.210526	0.016849	prophenoloxidase activating factor 1 [Scylla paramamosain]
MSTRG.28608	2.476667	5.623333	1.183026	0.016908	probable magnesium transporter NIPA1 [Penaeus vannamei]
MSTRG.10323	10.64333	5.16	-1.04451	0.017027	LOW QUALITY PROTEIN: pyridine nucleotide-disulfide oxidoreductase domain-containing protein 2-like [Penaeus vannamei]
LVAN11696	29.18	13.28333	-1.13536	0.017101	PREDICTED: betaine--homocysteine S-methyltransferase 1-like [Branchiostoma belcheri]
LVAN16399	1.33	3.993333	1.586167	0.017244	PREDICTED: uncharacterized protein LOC106470123 [Limulus polyphemus]
LVAN17854	0.253333	2.456667	3.277593	0.017306	PREDICTED: pentraxin-related protein PTX3-like [Hyalella azteca]
LVAN18583	3.56	1.476667	-1.26953	0.017306	PREDICTED: proton-coupled folate transporter-like [Cimex lectularius]
LVAN13647	9.766667	21.42333	1.133245	0.017341	-
LVAN16089	3.026667	1.266667	-1.25669	0.017356	GM20552 [Drosophila sechellia]
LVAN11581	0.006667	0.31	5.539159	0.017356	hypothetical protein CAOG_00428 [Capsaspora owczarzaki ATCC 30864]
MSTRG.29990	0.243333	2.823333	3.536394	0.017523	venom protease-like, partial [Penaeus vannamei]
MSTRG.41074	0.083333	2.473333	4.891419	0.017557	uncharacterized protein LOC113830405 [Penaeus vannamei]
MSTRG.9281	0.123333	37.02667	8.229858	0.017974	-
LVAN19336	1.766667	4.083333	1.208717	0.018271	Inactive serine/threonine-protein kinase TEX14 [Daphnia magna]
LVAN22245	1.476667	5.896667	1.997555	0.018419	PREDICTED: serine/threonine-protein kinase Genghis Khan-like [Hyalella azteca]
LVAN17322	4.26	13.12667	1.623575	0.018574	C-type lectin domain-containing protein [Fenneropenaeus chinensis]

LVAN20504	2.036667	5.153333	1.339296	0.018655	PREDICTED: sodium/glucose cotransporter 5-like [Limulus polyphemus]
LVAN15990	4.976667	2.243333	-1.14954	0.01871	GH10994 [Drosophila grimshawi]
LVAN03667	4.2	10.23333	1.284815	0.01885	PREDICTED: tubulin alpha-8 chain-like [Hyalella azteca]
LVAN13474	0.883333	4.25	2.266433	0.018866	-
LVAN08067	22.79	52.10333	1.192975	0.018909	PREDICTED: myotubularin-related protein DDB_G0290005-like isoform X4 [Hyalella azteca]
LVAN04272	2.906667	1.02	-1.5108	0.019075	PREDICTED: uncharacterized protein LOC108676894 [Hyalella azteca]
LVAN10047	0.12	1.086667	3.178803	0.019075	PREDICTED: serine-rich adhesin for platelets-like [Hyalella azteca]
LVAN09762	1.306667	2.823333	1.111508	0.01975	Iporin [Papilio xuthus]
LVAN11052	0.083333	6.586667	6.304511	0.020211	caspase [Eriocheir sinensis]
LVAN11692	8.16	16.60667	1.025121	0.020674	PREDICTED: run domain Beclin-1 interacting and cysteine-rich containing protein isoform X1 [Orussus abietinus]
LVAN13526	4.293333	9.713333	1.177868	0.020674	PREDICTED: long chain acyl-CoA synthetase 9, chloroplastic-like [Hyalella azteca]
MSTRG.1915	0.04	1.613333	5.333901	0.020744	-
LVAN22749	4.693333	11.95667	1.34913	0.020744	PREDICTED: LOW QUALITY PROTEIN: extended synaptotagmin-1-like [Hyalella azteca]
MSTRG.11670	0.001	3.25	11.66622	0.020749	uncharacterized protein LOC113808073 isoform X1 [Penaeus vannamei]
LVAN01048	4.313333	9.546667	1.146194	0.021186	AAEL008679-PB [Aedes aegypti]
LVAN15577	0.56	2.7	2.269461	0.021584	XK-related protein [Daphnia magna]
LVAN09558	0.056667	0.776667	3.776723	0.02168	PREDICTED: phospholipase D alpha 1-like [Hyalella azteca]
LVAN22748	2.746667	5.98	1.122464	0.021728	gag-pol polyprotein [Clonorchis sinensis]
LVAN14285	10.44667	21.67333	1.052878	0.021864	PREDICTED: ceramide glucosyltransferase-like [Limulus polyphemus]
LVAN19613	0.786667	3.42	2.120172	0.0221	PREDICTED: roquin-1 isoform X2 [Harpegnathos saltator]
LVAN21973	0.04	1.226667	4.938599	0.022169	PREDICTED: chondroitin proteoglycan-2-like [Neodiprion lecontei]
LVAN11204	1.243333	5.166667	2.055021	0.022211	PREDICTED: b(0,+)-type amino acid transporter 1-like, partial [Hyalella azteca]
LVAN20356	0.626667	0.2	-1.6477	0.022213	TPA_exp: pol polyprotein [Schistosoma mansoni]

LVAN03171	0.066667	1.12	4.070389	0.022771	Parp12a protein [Danio rerio]
LVAN14731	16.44	7.636667	-1.1062	0.023243	2-aminoethylphosphonate--pyruvate transaminase [Exaiptasia pallida]
LVAN12922	0.876667	3.84	2.131006	0.023268	PREDICTED: mucin-12-like [Hyalella azteca]
LVAN10240	0.173333	2.01	3.535574	0.023356	Carboxyl-ester lipase [Daphnia magna]
LVAN15259	2.27	6.513333	1.520704	0.023536	alpha-2-macroglobulin [Pacifastacus leniusculus]
MSTRG.22102	0.06	0.743333	3.630975	0.023706	-
LVAN09534	3.293333	13.45667	2.030701	0.023923	PREDICTED: uncharacterized protein LOC106655919 isoform X2 [Trichogramma pretiosum]
LVAN14568	0.25	1.106667	2.146221	0.024015	Carboxyl-ester lipase [Daphnia magna]
MSTRG.9114	2.493333	5.82	1.222943	0.024389	Eukaryotic translation initiation factor 3 subunit A [Collichthys lucidus]
LVAN08958	0.103333	0.516667	2.321928	0.024397	PREDICTED: plexin A3-like isoform X1 [Hyalella azteca]
MSTRG.11232	0.033333	0.693333	4.378512	0.024468	hypothetical protein FJT64_008618 [Amphibalanus amphitrite]
MSTRG.1500	3.11	0.423333	-2.87705	0.024592	CTLU [Penaeus vannamei]
MSTRG.13838	0.001	0.43	8.748193	0.024601	-
LVAN10328	0.69	2.17	1.653027	0.024658	Schwannomin-interacting protein 1 [Zootermopsis nevadensis]
MSTRG.40883	0.043333	2.403333	5.793416	0.025146	cuticle protein AMP1A-like isoform X1 [Penaeus vannamei]
MSTRG.24210	0.14	0.001	-7.12928	0.025149	-
LVAN16133	2.163333	5.046667	1.222075	0.025543	PREDICTED: histone-lysine N-methyltransferase 2D-like [Hyalella azteca]
MSTRG.23106	0.183333	1.963333	3.420764	0.025632	uncharacterized protein LOC113816771 [Penaeus vannamei]
MSTRG.35260	0.416667	2.33	2.483364	0.026093	-
LVAN21716	0.22	1.323333	2.588601	0.026096	PREDICTED: monocarboxylate transporter 12 [Nicrophorus vespilloides]
LVAN22772	828.4933	122.4033	-2.75885	0.026404	hemocyanin V4 [Litopenaeus vannamei]
LVAN10879	1.526667	4.156667	1.445042	0.027132	Retrovirus-related Pol polyprotein from transposon [Daphnia magna]
LVAN24928	0.001	0.286667	8.16323	0.027343	PREDICTED: glutamate-gated chloride channel-like [Hyalella azteca]
MSTRG.7738	5.286667	12.52667	1.244572	0.027408	PREDICTED: brain protein I3 [Papilio xuthus]
LVAN05801	0.213333	0.026667	-3	0.027951	PREDICTED: probable chitinase 3 isoform X1 [Hyalella azteca]

LVAN09990	89.17	181.31	1.023828	0.02814	heat shock protein HSP 90-alpha [Camelus ferus]
MSTRG.28912	1.966667	4.453333	1.179133	0.028767	uncharacterized protein LOC113821073 [Penaeus vannamei]
MSTRG.802	1.443333	0.133333	-3.4363	0.029209	neuropeptide-like protein 31 [Penaeus vannamei]
MSTRG.11496	51.48	108.8967	1.080876	0.029662	-
LVAN23769	10.16667	20.54333	1.014824	0.029992	PREDICTED: serine-rich adhesin for platelets-like [Hyalella azteca]
LVAN25487	1.056667	3.33	1.656002	0.030075	PREDICTED: excitatory amino acid transporter isoform X1 [Neodiprion lecontei]
LVAN12396	2.56	1.02	-1.32757	0.030376	PREDICTED: protein zntD-like [Hyalella azteca]
LVAN17778	0.09	1.39	3.949016	0.030815	cytochrome P450 CYP2 [Eriocheir sinensis]
LVAN22813	6.473333	16.25	1.327859	0.03093	-
LVAN21205	6.106667	16.23667	1.410798	0.031178	PREDICTED: serine/arginine-rich splicing factor 2-like [Hyalella azteca]
LVAN08939	0.546667	2.096667	1.939364	0.03148	PREDICTED: protein amalgam-like isoform X2 [Hyalella azteca]
LVAN24198	1.42	6.313333	2.152511	0.031543	-
LVAN17538	4.933333	10.81667	1.132621	0.031973	juvenile hormone esterase-like carboxylesterase 1 [Eriocheir sinensis]
LVAN19033	4.976667	2.453333	-1.02044	0.032262	PREDICTED: organic cation transporter protein-like [Limulus polyphemus]
LVAN21481	3.953333	8.07	1.029499	0.032359	PREDICTED: Ca(2+)/calmodulin-responsive adenylate cyclase isoform X4 [Bemisia tabaci]
LVAN03042	5.603333	2.236667	-1.32494	0.032383	Juvenile hormone-inducible protein [Anopheles darlingi]
LVAN25475	5.103333	1.233333	-2.04888	0.03241	fushi tarazu-factor 1 [Metapenaeus ensis]
LVAN03225	1.43	4.486667	1.649629	0.03241	PREDICTED: serine/threonine-protein kinase par-1-like isoform X4 [Megachile rotundata]
LVAN10557	1.243333	3.556667	1.516313	0.032645	PREDICTED: inter-alpha-trypsin inhibitor heavy chain H3-like isoform X2 [Lingula anatina]
LVAN03028	18.21667	48.89333	1.424379	0.032645	peritrophic membrane chitin binding protein [Culex quinquefasciatus]
LVAN19357	5.31	11.97333	1.173041	0.033352	PREDICTED: multifunctional protein ADE2 [Crassostrea gigas]
LVAN20755	3.52	10.34333	1.555054	0.033352	PREDICTED: membrane progestin receptor gamma-B-like [Hyalella azteca]
LVAN09310	0.896667	3.596667	2.004017	0.033495	PREDICTED: transforming growth factor-beta-induced protein ig-h3-like isoform X3

					[Hyalella azteca]
LVAN14859	0.3	2.393333	2.995987	0.033927	Star [Daphnia magna]
LVAN15851	4.266667	8.6	1.011227	0.033927	PREDICTED: phosphorylated CTD-interacting factor 1-like [Hyalella azteca]
LVAN12927	0.001	1.186667	10.2127	0.033958	peritrophic membrane protein 3, partial [Holotrichia oblita]
LVAN16916	13.76	32.34	1.232839	0.033958	actin 1 [Penaeus monodon]
MSTRG.37714	0.033333	1.24	5.217231	0.034133	uncharacterized protein LOC113827878 isoform X1 [Penaeus vannamei]
MSTRG.1595	0.56	1.72	1.61891	0.03443	cysteine and glycine-rich protein 1-like [Penaeus vannamei]
LVAN09308	0.596667	2.08	1.801586	0.034835	PREDICTED: uncharacterized protein LOC108677189 isoform X1 [Hyalella azteca]
MSTRG.33523	0.001	1.48	10.53138	0.034848	neuralized-like protein 4 [Penaeus vannamei]
LVAN10605	21.06667	42.37667	1.008308	0.034965	elongation of very long chain fatty acids-like protein [Marsupenaeus japonicus]
LVAN20461	2.756667	5.773333	1.06648	0.035592	Bromodomain and WD repeat-containing protein 3, partial [Stegodyphus mimosarum]
MSTRG.5291	1.736667	6.903333	1.990972	0.035592	-
LVAN09535	2.676667	6.313333	1.237964	0.035848	PREDICTED: armadillo repeat-containing protein 6-like [Hyalella azteca]
LVAN16504	6.8	17.64333	1.375517	0.035912	PREDICTED: receptor-type tyrosine-protein phosphatase alpha-like [Hyalella azteca]
LVAN12349	0.023333	0.713333	4.934112	0.035931	serine proteinase 1 [Portunus trituberculatus]
MSTRG.11174	5.566667	16.43333	1.56174	0.036144	lethal(2) giant larvae protein homolog 2 isoform X1 [Gadus morhua]
LVAN09345	0.193333	1.82	3.234776	0.036144	PREDICTED: G-protein coupled receptor Mth2-like [Hyalella azteca]
LVAN12727	0.001	0.633333	9.306821	0.036144	-
MSTRG.3021	4.136667	1.446667	-1.51574	0.036172	rho GTPase-activating protein gacU-like [Penaeus vannamei]
MSTRG.2842	6.09	13.64	1.16333	0.036932	-
LVAN07289	2.456667	8.81	1.84244	0.037667	PREDICTED: uncharacterized protein LOC100745028 [Bombus impatiens]
LVAN11986	0.716667	4.416667	2.623584	0.037705	Matrix metalloproteinase-24 [Zootermopsis nevadensis]
LVAN19925	0.976667	1.96	1.004915	0.037747	PREDICTED: rootletin-like isoform X4 [Lingula anatina]
LVAN16917	8.9	18.77	1.076551	0.037813	actin 1 [Penaeus monodon]
LVAN08158	44.41667	109.84	1.30623	0.037947	amylase I, partial [Litopenaeus vannamei]
LVAN20582	0.15	1.353333	3.173483	0.038406	PREDICTED: annexin-B12-like [Hyalella azteca]

LVAN19750	18.06667	38.93667	1.107799	0.038424	-
LVAN09431	0.283333	1.95	2.782902	0.039077	PREDICTED: histamine H2 receptor-like isoform X2 [Limulus polyphemus]
LVAN23494	1.61	6.51	2.015597	0.039453	crustacyanin C1, partial [Penaeus monodon]
LVAN08244	2.496667	5.176667	1.05202	0.040343	PREDICTED: protein sprint-like [Hyalella azteca]
LVAN12633	8.25	4.086667	-1.01347	0.040622	Proline dehydrogenase 2 [Daphnia magna]
MSTRG.9466	6.796667	1.686667	-2.01065	0.041149	filamin-A-like [Penaeus vannamei]
LVAN20732	5.863333	11.95667	1.028022	0.041424	Insulin-like peptide receptor [Orchesella cincta]
MSTRG.23502	0.07	0.793333	3.5025	0.041953	-
LVAN20275	1.156667	2.506667	1.115797	0.0425	PREDICTED: LOW QUALITY PROTEIN: titin-like [Agrilus planipennis]
MSTRG.35346	1.693333	0.373333	-2.18133	0.0425	-
MSTRG.29018	11.24	5.573333	-1.01203	0.0425	sarcosine dehydrogenase, mitochondrial-like, partial [Penaeus vannamei]
LVAN24548	0.04	0.426667	3.415037	0.042701	PREDICTED: serine/threonine-protein phosphatase 6 regulatory ankyrin repeat subunit B isoform X3 [Ceratitis capitata]
LVAN11200	2.633333	1.006667	-1.3873	0.042701	-
LVAN17724	0.336667	1.396667	2.052595	0.042701	PREDICTED: phospholipase DDHD1-like [Lingula anatina]
LVAN14672	0.396667	2.11	2.411244	0.04278	heat shock protein 21 [Macrobrachium rosenbergii]
LVAN15041	5.89	2.59	-1.18532	0.043168	AAEL014436-PA, partial [Aedes aegypti]
MSTRG.10644	0.91	4.33	2.250429	0.043186	-
MSTRG.6125	0.903333	2.416667	1.419688	0.04346	LOW QUALITY PROTEIN: SH3 domain-containing protein C23A1.17-like [Penaeus vannamei]
LVAN11429	45.71667	95.08	1.056422	0.044054	pacifastin heavy chain [Macrobrachium rosenbergii]
LVAN23513	0.001	1.156667	10.17576	0.044146	crustacyanin subunit C [Fenneropenaeus merguiensis]
LVAN04067	0.163333	1.176667	2.848815	0.044545	-
MSTRG.34652	19.26	39.22333	1.026104	0.044833	Eukaryotic translation initiation factor 4 gamma, partial [Procambarus clarkii]
LVAN15312	3.113333	8.313333	1.416967	0.044833	juvenile hormone esterase-like carboxylesterase 1 [Eriocheir sinensis]
LVAN03219	9.503333	20.94	1.139756	0.045566	PREDICTED: protein pinocchio [Eufriesea mexicana]

MSTRG.31158	0.82	2.466667	1.588867	0.045671	zinc finger protein 501-like [Penaeus vannamei]
LVAN01754	0.93	2.943333	1.662148	0.045699	PREDICTED: flocculation protein FLO11-like, partial [Hyalella azteca]
LVAN13471	0.473333	1.95	2.042546	0.046595	PREDICTED: serine/threonine-protein kinase PKH3-like [Hyalella azteca]
LVAN13969	2.486667	6.976667	1.488325	0.046725	PREDICTED: protein lifeguard 1-like isoform X2 [Hyalella azteca]
MSTRG.32531	5.473333	12.87	1.23352	0.047428	hypoxia inducible factor 1 alpha [Penaeus japonicus]
LVAN15667	1301.24	422.42	-1.62314	0.048514	beta-1,3-glucan-binding protein precursor [Astacus astacus]
LVAN18228	1.456667	4.083333	1.487077	0.049683	PREDICTED: tubulin alpha-3 chain-like [Hyalella azteca]
LVAN23840	0.001	0.596667	9.220781	0.049871	heat shock protein 21 [Macrobrachium rosenbergii]

**Metabolite_ID**	**Modes**	**R20523-0h**	**R20523-12h**	**Log2(fc)**	**VIP**	**Description**
M242T76	Negative ionization	15327.0448	136000.68	3.149462	2.783997	Dimethachlor cga369873
M339T27	Negative ionization	31133.4743	564382.54	4.180135	4.999438	Glyceraldehyde, 3-(dihydrogen phosphate)
M540T192_2	Negative ionization	444815.367	207098.31	-1.10289	2.471787	Phosphatidylcholine lyso alkyl 16:0
M88T336_2	Negative ionization	665236.04	765023.55	0.201638	3.404697	Sarcosine
M251T173	Negative ionization	1052315.26	1225296.2	0.219564	4.361574	Deoxyinosine
M147T387	Negative ionization	193344.876	363646.44	0.91136	3.43514	(S)-2-Hydroxyglutarate
M129T387_2	Negative ionization	169954.828	304897.71	0.843174	3.012932	Citraconic acid
M243T155	Negative ionization	4151921.35	3128241.5	-0.40843	3.421876	Uridine
M535T208	Negative ionization	260014.815	162950.14	-0.67416	1.584689	3-deoxy-d-glycero-d-galacto-2-nonulosonic acid
M243T200	Negative ionization	547955.25	325023.8	-0.75351	2.391126	Pseudouridine
M114T302	Negative ionization	1728096.93	2055186.7	0.250085	6.209826	Proline
M274T449	Negative ionization	57250.3658	82339.764	0.524304	1.333446	Glu-Lys
M253T44	Negative ionization	77628.2961	172293.68	1.150215	2.186294	Cis-9-palmitoleic acid
M225T352	Negative ionization	93244.9885	123196.93	0.401868	1.546357	L-Carnosine
M131T134	Negative ionization	42740.6997	153262.25	1.84232	2.501979	Hydroxyisocaproic acid
M487T154	Negative ionization	680270.578	301340.79	-1.17471	2.741631	Oxyresveratrol

M89T223	Negative ionization	59311.1951	122828.76	1.050272	2.076892	Dl-lactate
M208T86	Negative ionization	22702.8645	215586.31	3.247319	3.036153	4-morpholinopropanesulfonic acid
M257T140	Negative ionization	312927.838	890358.75	1.508557	6.254572	His-Cys
M180T142	Negative ionization	251726.439	613168.77	1.284427	3.750859	Acamprosate
M267T291	Negative ionization	59036.4545	84351.396	0.514806	1.387431	Leu-His
M172T188	Negative ionization	17060.1806	45296.719	1.408774	1.305147	Acetyl-dl-leucine
M203T412	Negative ionization	534833.056	426705.56	-0.32585	1.063229	Ala-asp
M318T181	Positive ionization	115768.052	167583.38	0.533642	1.469589	Trp-Ile
M291T460	Positive ionization	273971.666	660103.55	1.268666	3.522444	Argininosuccinic acid
M261T456	Positive ionization	219600.351	279569.73	0.348328	1.679328	Lys-Asn
M348T398	Positive ionization	145916.987	341839.53	1.228171	2.612111	Adenosine 2'-monophosphate
M244T74	Positive ionization	21301.4275	108653.33	2.35071	1.715822	N-trifluoroacetyl-l-phenylalanine methyl ester
M290T476	Positive ionization	249048.602	158969.91	-0.64767	1.4345	Asp-Arg
M645T259	Positive ionization	103147.881	58960.776	-0.80689	1.189141	8-iso-prostaglandin a2-biotin
M355T394	Positive ionization	131747.395	70932.432	-0.89326	1.301994	Vincamine
M752T214	Positive ionization	71075.7988	34389.983	-1.04737	1.048334	Germinaline
M232T255	Positive ionization	895618.506	2527366.3	1.496679	7.725978	Isobutyryl-l-carnitine
M419T297	Positive ionization	580421.955	364320.17	-0.6719	2.384541	Leu-Met-Arg
M498T447	Positive ionization	51439.8924	82901.081	0.688503	1.096201	Microcystin lr
M304T438	Positive ionization	1554305.78	2659982.3	0.775146	5.184947	Arg-glu
M156T363	Positive ionization	66554.7931	96117.825	0.530261	1.121341	Swainsonine
M312T437	Positive ionization	143292.13	185255.14	0.370554	1.351633	His-Arg
M376T439	Positive ionization	39242.1306	72052.042	0.876636	1.114029	Asn-Ser-Arg
M246T410	Positive ionization	1970481.35	2767221.7	0.48989	5.488895	Arg-Ala
M369T453	Positive ionization	333551.676	773540.57	1.213567	3.458136	Arg-Pro-Pro
M463T440	Positive ionization	304487.998	184004.2	-0.72665	2.004127	1-piperazinecarboxylic acid, 4-[(3-phenoxyphenyl)methyl]-, 2,2,2-

						trifluoro-1-(trifluoromethyl)ethyl ester
M487T440	Positive ionization	201650.529	123244.97	-0.71033	1.529865	4-androsten-17.beta.-ol-3-one glucosiduronate
M245T183	Positive ionization	752612.262	1025163.5	0.445875	3.373797	Leucylleucine
M260T366	Positive ionization	920699.707	1452772.1	0.658006	4.583618	Lys-Ile
M772T394	Positive ionization	39028.2827	77244.339	0.984909	1.095922	Venturicidin a
M482T197_3	Positive ionization	24161104.7	13888965	-0.79875	15.45203	1-hexadecyl-sn-glycero-3-phosphocholine
M402T395	Positive ionization	160207.089	293021.23	0.871067	1.94845	Val-Gln-Arg
M274T66	Positive ionization	6173012.06	8033957.7	0.380136	7.95157	Fenpropidin
M83T336	Positive ionization	151198.887	231596.16	0.615164	1.434192	4-methyl-1h-pyrazole
M430T444	Positive ionization	62674.2556	105554.67	0.752046	1.109467	Trp-Pro-Lys
M742T413	Positive ionization	137188.886	58906.041	-1.21968	1.429783	1-palmitoyl-2-(4-ketododec-3-enedioyl)phosphatidylcholine
M591T421	Positive ionization	119559.338	76037.462	-0.65294	1.063658	Urobilin
M491T450	Positive ionization	155955.95	109239.73	-0.51364	1.214627	5.alpha.-androstan-3.alpha.,17.beta.-diol-o-3-.beta.-glucuronic acid
M352T541	Positive ionization	117685.818	172967.41	0.55556	1.604896	Dipivefrine
M519T158_1	Positive ionization	186435.173	108734.19	-0.77787	1.69175	D-glucosamine 1-phosphate
M322T335	Positive ionization	538366.601	390710.15	-0.46249	2.158791	Arg-phe
M511T445	Positive ionization	178278.976	97564.157	-0.86971	1.341691	Asiatic acid
M531T295	Positive ionization	117454.074	72430.241	-0.69743	1.161791	Proscillaridin a
M129T336_2	Positive ionization	867413.427	1305476.9	0.589785	3.296556	4-ketopimelic acid
M516T290	Positive ionization	117457.469	72869.912	-0.68874	1.020495	Enzastaurin
M367T252	Positive ionization	112868.288	138471.81	0.294952	1.008682	Hirsuteine
M111T74	Positive ionization	643836.805	1044030.9	0.697397	2.724248	D-erythro-imidazolylglycerol phosphate
M496T197	Positive ionization	440587.533	202404.33	-1.12219	1.905348	1-o-hexadecyl-2-o-methyl-sn-glyceryl-3-phosphorylcholine
M342T367	Positive ionization	261376.238	175326.54	-0.57608	1.524739	7-acetyllycopsamine
M230T362	Positive ionization	183229.914	270314.94	0.560986	1.897851	Pro-asn
M253T176	Positive ionization	1175488.12	1571354.5	0.418749	4.47194	2'-deoxyinosine

M259T447	Positive ionization	3420963.27	2503225.3	-0.45061	5.391572	D-pyroglutamic acid
M158T255	Positive ionization	83959.8272	135102.23	0.68628	1.356174	Trans-4-(aminomethyl)cyclohexanecarboxylic acid
M134T395	Positive ionization	1029141.66	875436.84	-0.23337	2.038426	L-aspartic acid
M234T447	Positive ionization	125402.735	164440.1	0.390993	1.242127	Methylphenidate
M258T223	Positive ionization	92206.193	50588.354	-0.86606	1.148034	5-methylcytidine
M369T541_7	Positive ionization	9863306.7	15183403	0.622352	15.3746	Perindopril
M387T425	Positive ionization	80351.1266	108661.91	0.435456	1.07062	Asp-Pro-Arg
M311T361	Positive ionization	112080.569	147007.35	0.391352	1.256485	Tyr-Glu
M667T444	Positive ionization	31089.9883	145588.18	2.227371	1.980621	Prostaglandin a2-biotin
M235T140	Positive ionization	97853.0309	147960.75	0.596526	1.252285	Zolpidem
M248T430	Positive ionization	547556.461	924787.8	0.756115	3.78083	Lys-Thr
M926T394	Positive ionization	136132.912	75316.222	-0.85398	1.333113	Hoiamide a
M289T421	Positive ionization	232408.829	300568.84	0.371031	1.601593	Arg-Asn
M246T237	Positive ionization	335357.006	1137002.7	1.761466	5.292631	2-methylbutyryl-l-carnitine
M354T299	Positive ionization	1451007.07	1173859.9	-0.30579	2.50636	Proadifen
M310T294	Positive ionization	122551.875	160676.45	0.390766	1.303793	N-acetylneuraminate
M340T328	Positive ionization	895793.68	671647.35	-0.41546	2.627972	Propoxyphene
M161T525	Positive ionization	168364.791	195757.7	0.217479	1.027501	N.epsilon.-methyl-l-lysine
M337T36	Positive ionization	529731.824	924393.23	0.803245	4.028644	1-monolinoleoyl-rac-glycerol
M180T42	Positive ionization	1052225.67	717832.26	-0.55173	3.259322	.gamma.-aminobutyric acid
M136T159	Positive ionization	103574.887	191300.9	0.885169	1.80302	Adenine
M329T427	Positive ionization	113181.054	42713.065	-1.40588	1.122368	Pro-Gly-Arg
M705T259	Positive ionization	79802.9298	46047.151	-0.79333	1.02546	Voacamine
M244T359	Positive ionization	444906.179	619390.2	0.477347	2.797416	Pro-gln
M170T363	Positive ionization	136100.933	213609.63	0.6503	1.518289	1-methyl-l-histidine
M584T162	Positive ionization	133264.584	82331.674	-0.69477	1.230915	Dihydroergotamine

M126T223	Positive ionization	109128.891	61639.037	-0.82412	1.227639	5-methyl-2'-deoxycytidine
M218T429	Positive ionization	1314525.41	1698212.3	0.369475	3.474884	Ala-Lys
M388T278	Positive ionization	379362.484	288934.48	-0.39283	1.478089	Guan-fu base y
M480T196	Positive ionization	3471784.36	2038467.3	-0.76819	6.548999	1-(1z-hexadecenyl)-sn-glycero-3-phosphocholine
M369T394	Positive ionization	444819.312	321327.02	-0.46918	1.877622	Hirsutine
M317T366	Positive ionization	90686.7418	122572.72	0.434674	1.100577	Leu-Gly-Lys
M177T153	Positive ionization	44412.1919	126864.31	1.514259	1.551389	3,4-methylenedioxybenzyl methyl ketoximine
M143T281_2	Positive ionization	838575.209	651366.42	-0.36447	2.081893	Glycyl-l-norleucine
M284T156	Positive ionization	101238.494	32861.983	-1.62327	1.416154	Oxymorphone
M277T447	Positive ionization	3054730.53	2337200	-0.38626	4.861264	Gamma-l-glutamyl-l-glutamic acid
M227T337	Positive ionization	282598.224	446812.61	0.660918	2.197357	L-carnosine
M282T132	Positive ionization	87405.7226	169043.25	0.951593	1.557043	1-methyladenosine
M294T326	Positive ionization	163248.349	204312.64	0.32371	1.186383	Phe-lys
M293T38_2	Positive ionization	104739.661	139970.19	0.418312	1.21353	Cis-13-eicosenoic acid
M313T159	Positive ionization	2184047.75	1562160.1	-0.48346	3.93767	Phe-phe
M132T339	Positive ionization	66379.0506	113491.55	0.773785	1.096235	Hydroxyproline
M284T362	Positive ionization	284448.208	406930.67	0.516617	1.900082	His-Gln
M249T396	Positive ionization	4120796.66	3222000.6	-0.35497	4.783394	Thr-Glu
M144T300	Positive ionization	162133.398	228788.2	0.496831	1.306693	L-homoarginine
M399T463	Positive ionization	538704.944	362038.56	-0.57335	1.197932	S-adenosylmethionine
M219T113	Positive ionization	70214.6191	116586.99	0.731563	1.154317	Eseroline
M144T269	Positive ionization	542303.128	684789.06	0.33656	2.140833	Stachydrine
M120T251_2	Positive ionization	26560713.3	25039362	-0.0851	4.955817	2-amino-1-phenylethanol
M150T235	Positive ionization	38802.1782	101550.81	1.387992	1.470273	7-methyladenine
M150T101	Positive ionization	244886.206	155759.69	-0.65279	1.454266	Pyridoxal
M393T328	Positive ionization	112283.529	64815.627	-0.79273	1.159685	Ala-Phe-Arg

M186T159	Positive ionization	491312.77	357568.08	-0.45842	2.064809	N-acetyl-d-galactosamine
M245T408	Positive ionization	1610040.46	1918695.5	0.253029	3.578267	Pro-Glu
M191T421	Positive ionization	192105.961	145563.9	-0.40025	1.216065	Gly-Asp
M232T525	Positive ionization	443444.115	108824.03	-2.02676	2.855123	N-.alpha.-(tert-butoxycarbonyl)-l-isoleucine
M503T407	Positive ionization	91920.1871	176622.12	0.942212	1.686251	Cyasterone
M147T333	Positive ionization	264811.118	216147.39	-0.29295	1.035362	Ala-Gly
M261T436	Positive ionization	755445.513	888585.21	0.234182	2.33398	Asn-Lys
M559T415	Positive ionization	223533.556	157426.17	-0.50582	1.414851	Karbutilate
M508T194	Positive ionization	3265909.5	1986915.2	-0.71695	4.42768	1-(1z-octadecenyl)-sn-glycero-3-phosphocholine
M346T442	Positive ionization	104624.214	137860.72	0.397995	1.196847	Gly-Asn-Arg
M268T167	Positive ionization	550432.837	901593.04	0.71191	3.651737	Adenosine
M504T407	Positive ionization	76209.3661	142026.14	0.898116	1.005163	Glycocholic acid
M343T36	Positive ionization	61602.9262	105344.42	0.774043	1.258129	Docosahexaenoic acid methyl ester
M505T385_2	Positive ionization	128343.605	92032.489	-0.4798	1.072306	Betamethasone dipropionate
M293T456	Positive ionization	116515.019	73069.237	-0.67318	1.039094	Ethylenediaminetetraacetic acid
M511T166	Positive ionization	142305.722	95473.308	-0.57582	1.130685	Leukotriene d4 methyl ester
M258T170	Positive ionization	944937.859	558267.11	-0.75926	3.201507	2'-o-methylcytidine
M333T452	Positive ionization	145540.295	265355.93	0.86651	1.690808	Glu-Gly-Lys
M333T76	Positive ionization	653153.427	426107.8	-0.6162	2.549359	Trp-Gln
M203T254_2	Positive ionization	588432.389	750942.26	0.351825	2.334413	L-alanyl-l-norleucine
M305T38	Positive ionization	60633.0098	98165.529	0.695113	1.165968	Arachidonic acid (peroxide free)
M263T39	Positive ionization	644446.908	837433.73	0.377914	2.943449	2-linoleoylglycerol
M204T450_2	Positive ionization	1378634.9	1531115.4	0.151343	2.767646	Gly-Lys
M391T222	Positive ionization	99502.6031	59710.185	-0.73676	1.060751	Prostaglandin g2
M86T231	Positive ionization	149104.526	182666.03	0.292884	1.233279	1,5-pentanediamine
M337T191	Positive ionization	151267.072	216220.78	0.515407	1.42565	1-palmitoyl-2-linoleoyl-rac-glycerol

M170T106	Positive ionization	147791.811	198796.4	0.427725	1.07545	4-chloroamphetamine
